# Progress in Strain Engineering of 2D‐Integrated Heterostructures for Ultrasensitive Sensors

**DOI:** 10.1002/advs.75506

**Published:** 2026-05-04

**Authors:** That Buu Ton, Tuan Sang Tran, Duc Khanh Tran, Van Thanh Dau, Dzung Viet Dao

**Affiliations:** ^1^ School of Engineering and Built Environment Griffith University Brisbane Queensland Australia; ^2^ Queensland Quantum and Advanced Technologies Research Institute Griffith University Brisbane Queensland Australia

**Keywords:** 2D integration, 2D materials, sensing applications, strain engineering, vdW heterostructures

## Abstract

Two‐dimensional (2D) integrated heterostructures have emerged as a cornerstone in the advancement of next‐generation sensor technologies. These heterostructures, which combine materials with different dimensionalities, have led to significant breakthroughs in sensing performance and device integration. Owing to their tunable bandgaps and exceptional mechanical flexibility, the application of strain engineering in 2D‐integrated heterostructures represents a dynamic and rapidly evolving research frontier. This review presents a comprehensive assessment of the current progress in strain engineering applied to 2D‐integrated heterostructures. The review outlines the fundamental principles and distinctive properties exhibited in 2D‐integrated heterostructures under applied strain. State‐of‐the‐art 2D integration strategies and strain‐induction techniques are then examined, followed by an in‐depth analysis of key sensing mechanisms enabled by strain modulation. Recent advances in ultrasensitive sensing applications are highlighted, demonstrating the immense potential of strain‐engineered 2D‐integrated heterostructures. Finally, current challenges and outstanding bottlenecks are assessed, and future research directions are proposed, highlighting opportunities that may redefine the role of 2D‐integrated heterostructures in next‐generation sensing technologies.

## Graphical abstract

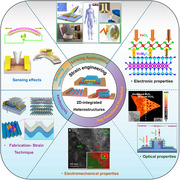

## 1 Introduction

Strain engineering has been extensively recognised as a powerful approach for tailoring the physical and functional properties of materials. By applying external mechanical forces, it is possible to systematically alter intrinsic atomic configuration parameters, such as lattice constants, bond angles, and atomic positions, thereby inducing modifications in the atomic structure at the quantum level [1, 2]. This alteration can disrupt the inherent compactness and organised structure of the crystal lattice, thereby generating novel attributes primarily linked with physical phenomena, encompassing electrical, optical, magnetic, thermal, and valleytronic properties [3]. The application of strain engineering in materials structures has recently gained prominence across various fields, including optoelectronics, thermionics, spintronics, and quantum emitters [4, 5].

Two‐dimensional (2D) materials, a category of atomically thin crystals distinguished by their exceptional physicochemical and optoelectronic properties, demonstrate considerable sensitivity to strain engineering, largely attributable to their significant anisotropic nature [6]. Previous studies have shown that modifications to lattice structures, the incorporation of defects, and surface functionalisation can substantially influence their electrical and physical properties [7, 8, 9]. The engineering of 2D materials is significantly advanced through the construction of Van der Waals (vdW) heterostructures. This concept was initially proposed by Koma's vdW epitaxy and subsequently developed by Geim and Grigorieva [10, 11]. These vdW heterostructures, which are formed by vertical stacking diverse 2D materials, facilitate the design of tailored 2D‐integrated heterojunctions, each exhibiting distinct characteristics. Furthermore, vdW heterostructures enable the integration of materials across varying dimensionalities (e.g., 0D/2D, 1D/2D, and 2D/3D), utilising precisely controlled stacking sequences to achieve novel and emergent properties [12, 13, 14, 15].

Strain engineering has become a significant approach for modifying the physical characteristics of materials, thereby facilitating improved performance across numerous applications [16, 17]. Traditionally, investigations in this area have concentrated on bulk (three‐dimensional) systems, particularly concerning strain relaxation processes and their influence on material properties [17, 18, 19, 20, 21]. Recent developments have redirected focus toward 2D and mixed‐dimensional 2D‐integrated systems, motivated by the distinctive mechanical and optoelectronic attributes of atomically thin materials [22, 23]. Consequently, this has presented new avenues for performance enhancement and compatibility, which are still largely unexamined. Although considerable investigation has been devoted to the impact of strain on monolayer 2D materials, a significant deficit in understanding exists concerning the applications of strain in 2D‐integrated heterostructures. These structures possess considerable promise for surpassing conventional 2D materials across diverse applications, including optoelectronics and sensing [24, 25]. While several reviews have explored 2D vdW heterostructures and their application in sensing and optoelectronics [3, 12, 26, 27], systematic and thorough analyses that concentrate on strain engineering as a method for modulating and controlling the critical properties of 2D‐integrated heterostructures for ultrasensitive sensing are regrettably quite limited.

**FIGURE 1:**
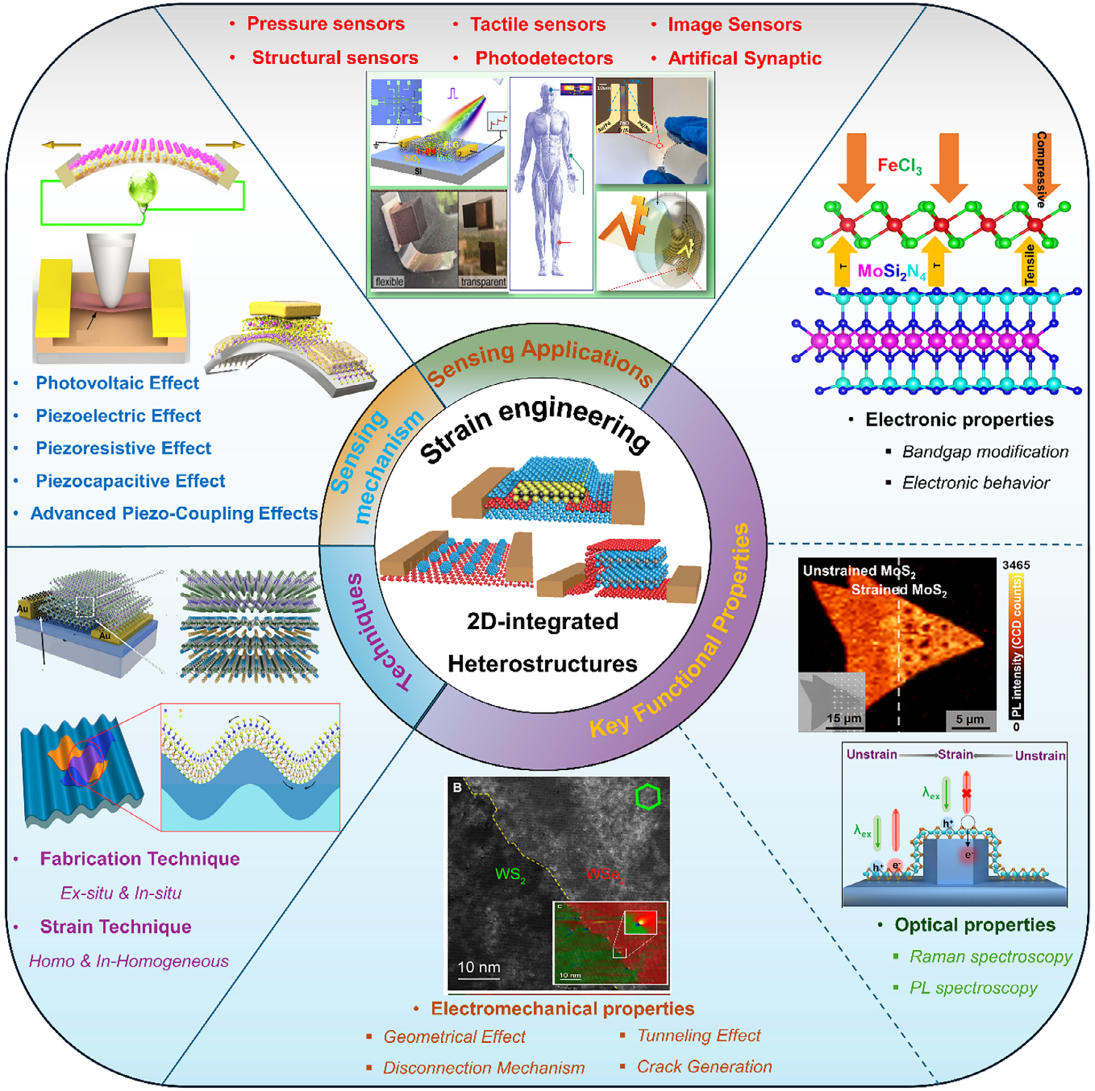
Summary diagram of the review.

While several recent reviews have provided comprehensive overviews of strain engineering in nanomaterials [2, 27, 28, 29], these efforts only focused on isolated or individual 2D materials. In contrast, the present review is distinctively dedicated to the emerging and fundamentally more complex domain of 2D vdW heterostructures. The discussion systematically explores the unique strain‐induced functional properties, such as bandgap modulation and distinct electronic, optical, and electromechanical behaviors. In addition, key sensing mechanisms are comprehensively discussed and the most advanced applications in the field are highlighted. Given that strain engineering in 2D heterostructures remains at an early stage, with significant bottlenecks in deterministic fabrication and advanced nanoscale characterisation, this review aims to bridge the gap between current limitations and future potentials, providing a timely, novel, and critical roadmap for the next generation of strain‐engineered devices (Figure 1).

## 2 Fundamentals of Strain Engineering in 2D‐Integrated Heterostructures

### 2.1 2D Materials

**FIGURE 2:**
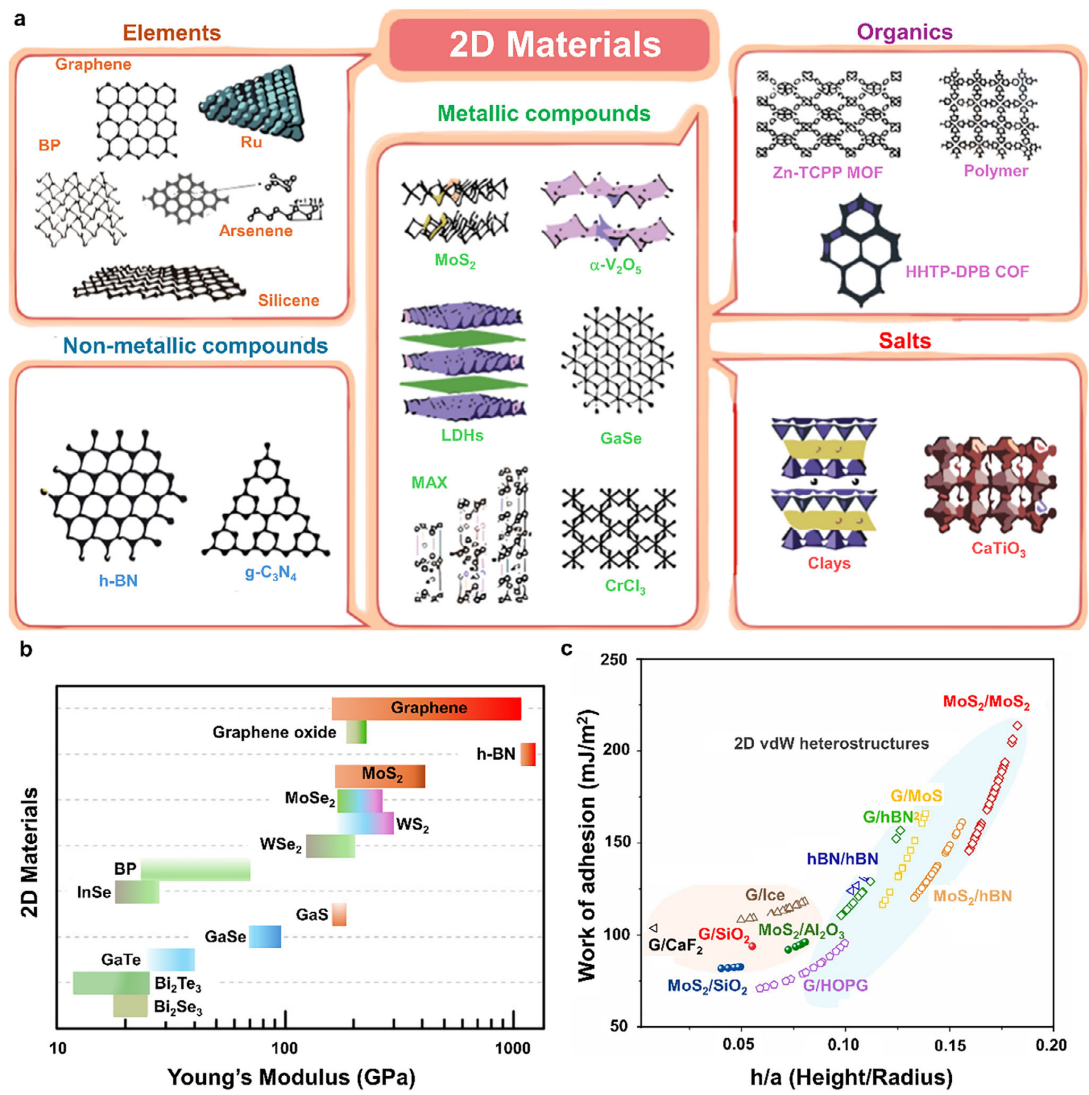
a) Structure and classification of different 2D materials. Reproduced with Creative Commons CC BY license [39]. Copyright 2022, Wiley. (b) The Young's modulus of 2D materials. Reproduced with permission [3]. Copyright 2020, Wiley. (c) The adhesion values of various 2D materials interfaces were estimated according to blister profiles, including many interfaces found in 2D heterostructures. Reproduced with permission [44]. 2018, PNAS.

Since the isolation of graphene in 2004, 2D materials, ranging in thickness from a single atomic layer to several nanometers, have undergone significant advancements in materials research [30, 31, 32]. Graphene has served as the foundation for the fabrication and advancement of other 2D materials, including transitional metal dichalcogenides (TMDs), hexagonal boron nitride (h‐BN), black phosphorus (BP), and phosphorene [33, 34, 35, 36, 37]. The classification of 2D materials is based on the systematic arrangement of elements, encompassing metallic compounds, non‐metallic compounds, organic substances, and salts [30, 38]. Figure 2a illustrates representative members in the 2D material family, with these materials can be built in layers, which makes them useful in many applications, including mechanical, electrical, and optical domains [39].

The excellent mechanical properties of 2D materials make them very useful for flexible sensing devices. The Young's modulus and breaking strain limit of 2D semiconductors are greatly affected by the types and strength of the bonds, as well as the structure of the crystal lattice [12]. Figure 2b presents the Young's modulus values associated with a range of 2D materials [3]. Graphene, specifically, exhibits exceptional mechanical properties; it possesses a Young's modulus of 1 TPa and can withstand mechanical strain surpassing 25% without structural failure [40, 41]. Furthermore, monolayer TMDs, such as MoS_2_ and WS_2_, demonstrate a Young's modulus of approximately 270 GPa, thereby establishing them as promising candidates for advanced mechanical sensors, particularly in flexible, wearable, and miniaturised applications [27].

In the domain of strain sensing utilising 2D‐integrated heterostructures, a critical parameter for assessing out‐of‐plane mechanical effects is the measurement of adhesion between 2D material and its integrated components [42]. Recent investigations have explored the role of surface features, such as wrinkle curvature and bubble formation in estimating adhesion energy between 2D materials and their substrates [27, 43]. The aspect ratio (h/a), with h and a corresponding to the centre height and radius of the blister, respectively, demonstrates its relevance to the work of adhesion of heterostructures [27, 44]. These approaches provide valuable insights into interfacial mechanics, which are critical for optimising the transfer of strain and device performance (Figure 2c). Understanding the adhesion energy at the interfaces between 2D‐integrated materials is particularly advantageous for the design of strain‐engineered devices that leverage out‐of‐plane deformation in 2D materials and 2D‐integrated heterostructures [27, 44].

### 2.2 2D‐Material Heterostructures

#### 2.2.1 Forming Mechanisms of 2D‐Integrated Heterostructures

**FIGURE 3:**
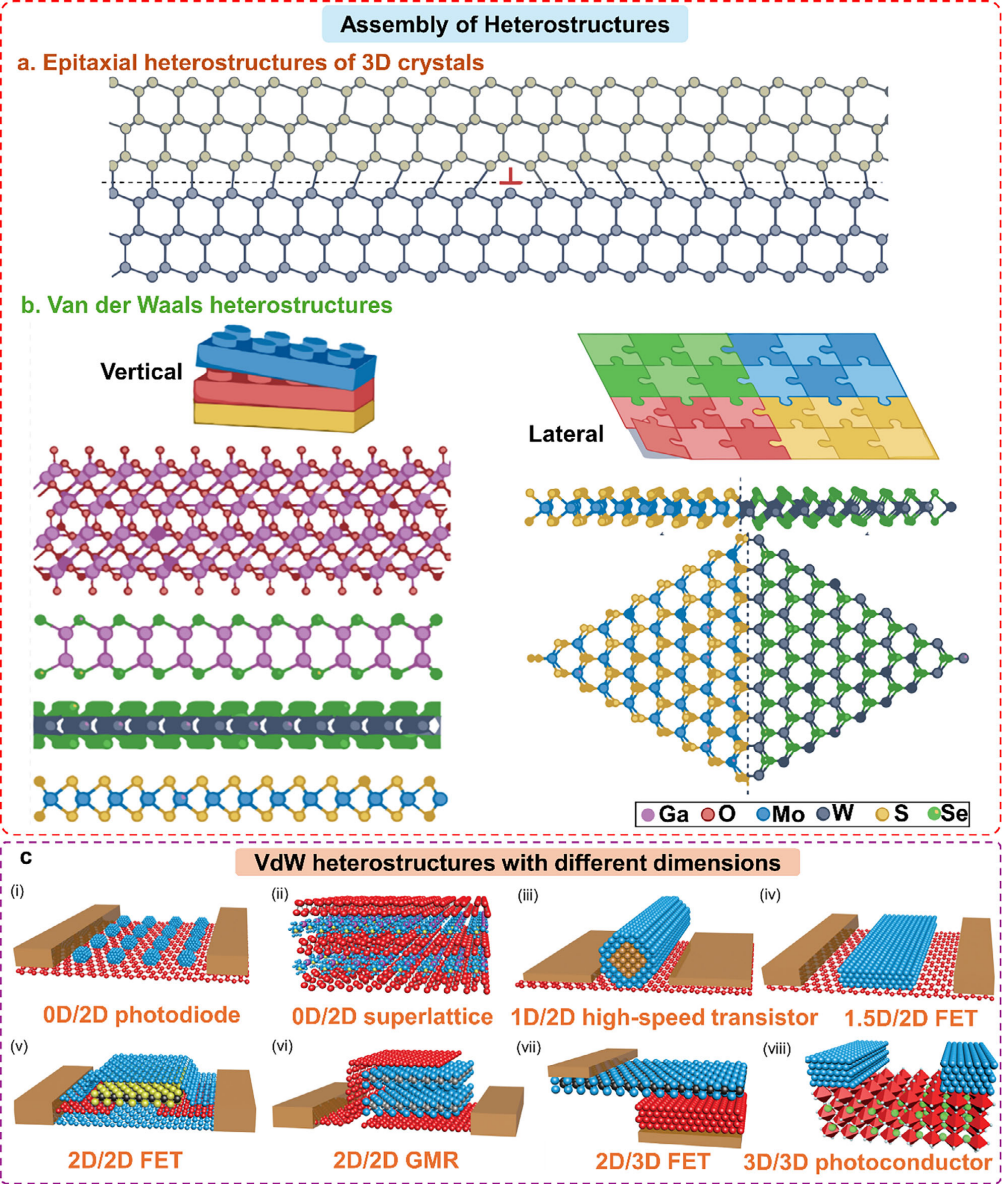
Assembly of heterostructures with two main approaches. (a) Epitaxial heterostructures with the integration of 3D crystals require strict control of lattice mismatch, (b) Van de Waals heterostructures with vertical heterostructures involve the stacking of layers held together by vdW forces, which provides the highest flexibility in integrating 2D and 3D substrates. Lateral heterostructures require a low‐dimensional equivalent. Reproduced with permission [11]. Copyright 2022, Springer Nature. (c) Van der Waals heterostructure devices formed with different dimensions: The devices are arranged in order of increasing dimensions: (i) 0D/2D photodiode created by vdW integration of quantum dots plasmonic nanoparticle (blues) on top of graphene (red), (ii) 0D/2D high‐order superlattice formed by intercalalting 0D molecules (blue) into a 2D phosphorene (red), (iii) 1D/2D high‐speed transistor created by vdW integration of 1D coreshell nanowire (blue) with 2D graphene (red), (iv) 1.5D/2D field‐effect transistor (FET) created by 1.5D Al_2_O_3_ nanoribbon (blue) on top of 2D graphene (red), (v) 2D/2D FET with 2D MoS_2_ as the channel (yellow and black), 2D dielectric BN (blue) as the encapsulation layer and 2D graphene (red) as the contact electrodes, (vi) 2D/2D giant magnetoresistance (GMR) device with 2D CrI_3_ (blue) and 2D graphene (red), (vii) 2D/3D tunneling FET created by 2D MoS_2_ (blue and black) and 3D Ge (red), and (viii) 3D/3D photoconductor with the vdW integration of damage‐free metal contacts (blue) on perovskite (red). Reproduced with permission [15]. Copyright 2019, Nature.

Van der Waals (vdW) force is a weak intermolecular attraction between atoms or molecules. VdW integration, achieved by stacking two or more layered materials, provides a flexible method for constructing layered systems. This method allows for the assembly of diverse materials, from 2D crystals to well‐terminated bulk (3D) substrates, which are maintained by vdW interfacial interactions [45]. This approach preserves the inherent characteristics of each individual layer, while simultaneously enabling the development of novel functionalities, specifically through the formation of heterojunctions, which are not possible in isolated materials [46]. In contrast, traditional epitaxy techniques are limited in their ability to construct such heterostructures due to the stringent requirements for lattice and symmetry compatibility among dissimilar 2D‐3D crystals (Figure 3a) [11]. This usually results in structural defects when there is a mismatch in lattice parameters or crystal symmetry between different materials. However, vdW assembly circumvents these limitations entirely. This technique enables the integration of materials with diverse crystal structures, symmetries, and lattice properties while allowing precise control over the stacking sequence, orientation, and twist angle [47]. Such flexibility supports the construction of both vertical and horizontal heterostructures (Figure 3b) [11, 48]. Stacks may incorporate both 2D and surface‐terminated 3D materials, provided that the interconnections remain exclusively vdW bonds [48]. The structural freedom afforded by vdW assembly facilitates the engineering of heterostructures with customised electronic properties at their interfaces, including the formation of quantum wells and superlattices. These capabilities have led to significant technological advancements, particularly in the development of broadband photodetectors and flexible systems [49, 50].

Two‐dimensional materials have become a promising area for developing new optical and electronic devices. A crucial element of these advancements lies in the ability to fabricate high‐quality heterostructures, which are assembled from disparate materials and held together by vdW forces [14]. Although early investigations predominantly centred on 2D–2D heterostructures, the ubiquitous character of vdW forces has facilitated the incorporation of 2D materials with components exhibiting varying dimensionalities [51, 52]. These vdW interactions allow for the assembly of 2D layers on the surfaces of 3D bulk materials (3DBMs) without the constraints of lattice matching, thus facilitating the design of high‐performance vdW‐based electronic architectures [53]. This method relaxes the stringent chemical and physical compatibility requirements of traditional growth method [54], allowing for a wide range of tunability in the optical and electronic properties of the resulting heterostructures [55]. Initial demonstrations of vdW integration focused on the combination of arbitrary 0D, 1D, 2D, and 3D materials, which can be achieved in a dry, sacrificial‐layer‐free, and scalable manner [56, 57, 58]. This mixed‐dimensional integration paradigm has opened new avenues for material integration at the nanoscale, leading to the realisation of advanced devices, including broadband photodetectors with ultrahigh speed and atomically thin transistors with unprecedented speed and flexibility (Figure 3c) [15, 23, 59]. Currently, the integration of multidimensional components (e.g., 0D/2D, 1D/2D, 2D/2D or 2D/3D) creates unique models and distinct mechanical challenges compared to traditional homogeneous systems. A critical factor determining device performance is the strain transfer ability, which varies considerably depending on the interface dimension. At a typical 2D/2D vdW interface, proper contact between regions allows for relatively uniform strain transfer, primarily governed by van der Waals shear forces between layers [60]. However, a major challenge arises from slip between layers; when applied macroscopic strain exceeds the critical interface shear strength, the 2D layers slip against each other, resulting in severe strain transfer degradation and significant sensing hysteresis [27]. In contrast, the integration of 1D structures (e.g., nanowires or nanotubes) with 2D films creates line‐to‐region contact. The 1D component acts as a local stressor at the nanoscale level, forcing the superimposed 2D film to take on a “tent” morphology [10]. This configuration results in highly inhomogeneous strain transfer, characterised by a huge local strain gradient. While this structural mismatch poses significant challenges in maintaining global device homogeneity, it offers unique opportunities for designing local quantum emission sites and exciton funnels [1, 61]. Furthermore, in practical integrated systems, interface defects such as atomic vacancies, structural wrinkles, or polymer residues from transfer processes will disrupt strain homogeneity [62, 63]. These defects act as rigid pinning centres, causing local stress concentration in the continuous strain field. From a device performance perspective, this inhomogeneous stress distribution is often detrimental. It accelerates mechanical fatigue, causing premature fracture under cyclic loading and resulting in unpredictable carrier scattering that degrades overall carrier mobility [64, 65]. However, these highly strained defect sites exhibit high local surface energy. This mechanical strain makes them hyperreactive sites, offering a unique opportunity to significantly enhance the chemical reactivity and sensitivity of heterostructures for advanced gas and electrochemical sensing applications [66, 67].

#### 2.2.2 Mechanical Strength of 2D‐Heterostructuresss

The mechanical behavior of 2D heterostructures under applied strain differs significantly from that of conventional bulk materials, primarily due to the prominence of surface and interfacial phenomena. The weak vdW interactions between 2D layers enable individual deformation responses while maintaining structural integrity. This characteristic enables innovative approaches to strain engineering that are not feasible in bulk systems [68]. Understanding the unique behavior of 2D‐integrated heterostructures requires a deep dive into the interlayer shear coupling and adhesion mechanisms, which ultimately govern how efficiently strain is transferred and how stable the structure remains under mechanical load [69].

**FIGURE 4:**
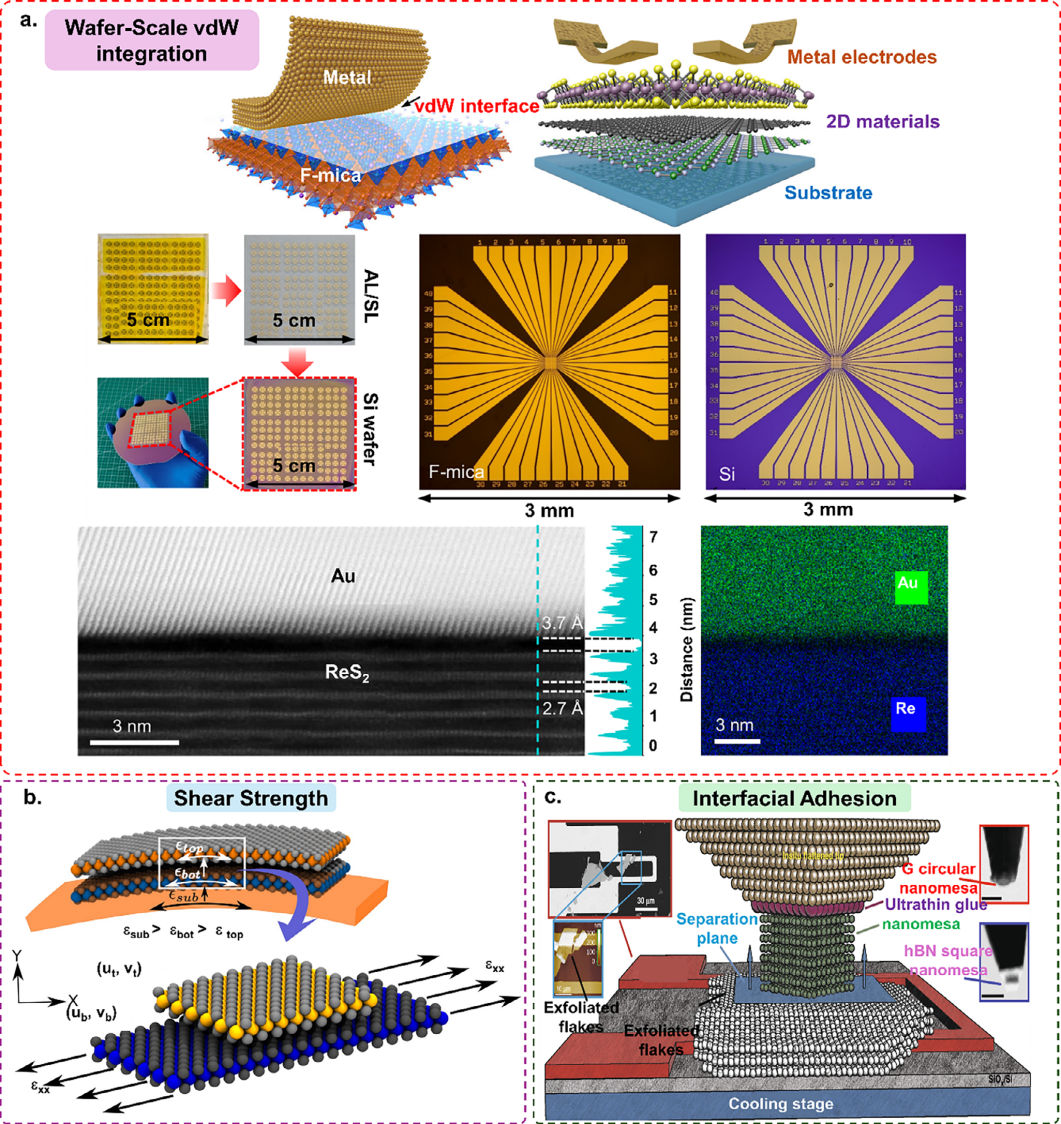
a) Highly reproducible vdW integration of 2D electronic devices on the wafer scale. Reproduced with permission [80]. Copyright 2024, Springer Nature. (b) Schematic of the 2D shear‐lag model in flexible 2D vdW heterostructures. Reproduced with permission [71]. Copyright 2016, Springer Nature. The top figure demonstrates the setup experiment, in which the flexible substrate (orange color) is used to make deformation. The bottom figure illustrates the 2D heterostructure when external stresses are applied (followed by the direction of the arrows). (c) Schematic of the interfacial adhesion measurement using an AFM tip. Reproduced with permission [40]. Copyright 2020, Springer Nature. The methods employ nano‐sized 2D crystals with precisely specific geometric shapes at the AFM tips, which facilitate accurate measurement of the contact area and interfacial adhesion energy at 2D vdW interfaces.

In this section, we will discuss the mechanisms involved in creating 2D‐integrated heterostructures, followed by their structural limitations under applied strain. From a structural design perspective, the construction of 2D‐integrated heterostructures using lateral, vertical, and heterogeneous configurations offers new insights and expands the potential of 2D materials for advanced sensing applications. Each design strategy provides unique pathways to tailor interfacial properties and device performance [12]. Specifically, shear strength and adhesion are two crucial mechanical properties that dictate strain transfer efficiency and structural integrity. Shear strength refers to the maximum in‐plane stress an interface can withstand prior to relative sliding between adjacent layers, whereas adhesion energy measures the energy required to separate bonded layers (Figure 4b,c) [40, 41, 70, 71]. These properties are particularly significant in 2D heterostructures because of their exceptionally high surface‐to‐volume ratio, which amplifies adhesive forces in numerous processes associated with device fabrication and operation [40].

In 2D materials, shear strength values exhibit considerable variation, spanning from 40 kPa in bilayer graphene to 8.35 MPa in MoS_2_. These disparities arise from surface roughness, chemical modifications, and prevailing environmental factors. For example, in MoS_2_/WS_2_ heterostructures, this early‐onset slippage restricts the transfer of strain between adjacent layers to approximately 1–2% [72]. This fundamental mechanical bottleneck is governed by the non‐covalent nature of the interface. According to the shear‐lag model, the distribution of strain across 2D nanosheets is not uniform. In theoretical calculation, interfacial shear stress builds up from the edges and reaches a maximum toward the centre [73]. Consequently, the edges of the 2D material experience the highest stress gradients and are particularly prone to partial debonding. Interfacial slippage occurs when the applied strain surpasses the critical interfacial static friction, which is determined by the vdW adhesion energy [73]. This slippage serves as a physical limit, limiting the highest attainable strain and severely reducing strain transfer efficiency at higher loading levels. The adhesion energy in 2D heterostructures is typically quantified in mJ/m^2^. As an illustration, MoS_2_/MoS_2_ exhibits a value of 174 mJ/m^2^, whereas graphene/SiO_2_ displays a value of 93 mJ/m^2^ [40].

Alongside inherent mechanical constraints, external environmental factors significantly influence interfacial degradation in practical devices. Environmental influences, such as airborne contaminants and humidity, can significantly diminish adhesion, potentially leading to a decrease of up to 50% compared to optimal conditions [57, 74]. Specifically, ambient water molecules can intercalate into the vdW gap, acting as an interfacial lubricant that drastically lowers adhesion energy and promotes early slippage [75]. Furthermore, variations in operating temperature cause out‐of‐plane deformations, such as rippling or wrinkling, due to the mismatch in the Coefficient of Thermal Expansion (CTE) between the 2D material and the flexible substrate, thereby physically disconnecting the interface [40]. Over time, repeated dynamic loading cycles lead to structural fatigue and the accumulation of microscopic interfacial defects [15]. Ultimately, these interfacial mechanics directly impact long‐term sensor reliability. From a device engineering perspective, stress‐induced slippage and environmental degradation result in significant mechanical hysteresis, degraded sensitivity (lower total gauge factor), and notable baseline drift during operation [76]. To mitigate these issues, specialised structural designs, such as utilising metal contact electrodes for mechanical “edge‐clamping” to pin the 2D layer [77], or employing robust encapsulation layers (e.g., hBN, Al_2_O_3_) to isolate the interface from ambient moisture, are essential for ensuring stable, long‐term performance [78, 79]

### 2.3 Strain‐Induced Modification of Key Functional Properties

A significant advancement in the fabrication of strain‐engineered heterostructures has recently emerged in the field of 2D vdW materials [77]. Monolayer transition metal dichalcogenides (TMDs), such as MoS_2_, MoSe_2_, and WS_2_, are highly regarded materials because of their direct bandgaps, significant effective carrier masses, strong excitonic effects, and unique spin‐valley interactions [81]. Unlike traditional epitaxial heterostructures, 2D vdW heterostructures are distinguished by their atomically thin layers, sharp, clean interfaces, no dangling bonds, and a considerable tolerance to lattice mismatches [82]. These inherent characteristics not only contribute to enhanced structural integrity but also facilitate precise strain control. Furthermore, they provide remarkable tunability and multifunctionality, which are essential for realising advanced ultrasensitive sensing capabilities. The expanding array of 2D materials broadens the potential for constructing vdW heterostructures with tailored properties [12]. Recent theoretical investigations have demonstrated the tunability of 2D vdW heterostructures’ properties through external stimuli. These stimuli encompass electric fields, mechanical strains, interlayer spacing, and twist angles [83, 84]. Currently, DFT is considered an extremely powerful tool for predicting the profound effects of strain on 2D heterostructures [83]. However, it is crucial to explicitly distinguish these idealised theoretical boundaries from experimentally validated phenomena. The achievement of theoretically predicted strain limits for ultrasensitive sensors is mostly hindered by intrinsic material discrepancies. Computational simulations typically model pristine, infinite crystalline lattices where transition metal dichalcogenides (TMDCs) like MoS_2_ are predicted to sustain extraordinary critical fracture strains exceeding 10 – 15% [84]. In reality, 2D layers synthesised via chemical vapour deposition (CVD) or mechanical exfoliation inherently contain point defects, such as chalcogen vacancies, and grain boundaries. These imperfections act as severe stress concentrators, drastically reducing the experimental fracture toughness and causing premature mechanical failure at much lower strain thresholds, typically around 2 – 4% [85, 86]. Moreover, while DFT can impose arbitrary strain profiles to exploit phenomena, creating these precise fields experimentally is daunting. Current techniques, such as draping 2D heterostructures over nanopillars or employing AFM indentation, frequently introduce uncontrolled out‐of‐plane deformations like wrinkling or crumpling. Consequently, isolating pure, highly uniform in‐plane strain gradients without disrupting the active heterostructure interface remains a formidable fabrication challenge [87]. In this section, we provide comprehensive reviews detailing the controlled band alignment and the modulation of electronic, optical, and electromechanical properties, especially under strain, supported by the most recent and state‐of‐the‐art computational and experimental studies.

#### 2.3.1 Strain‐Induced Bandgap Modification

**FIGURE 5:**
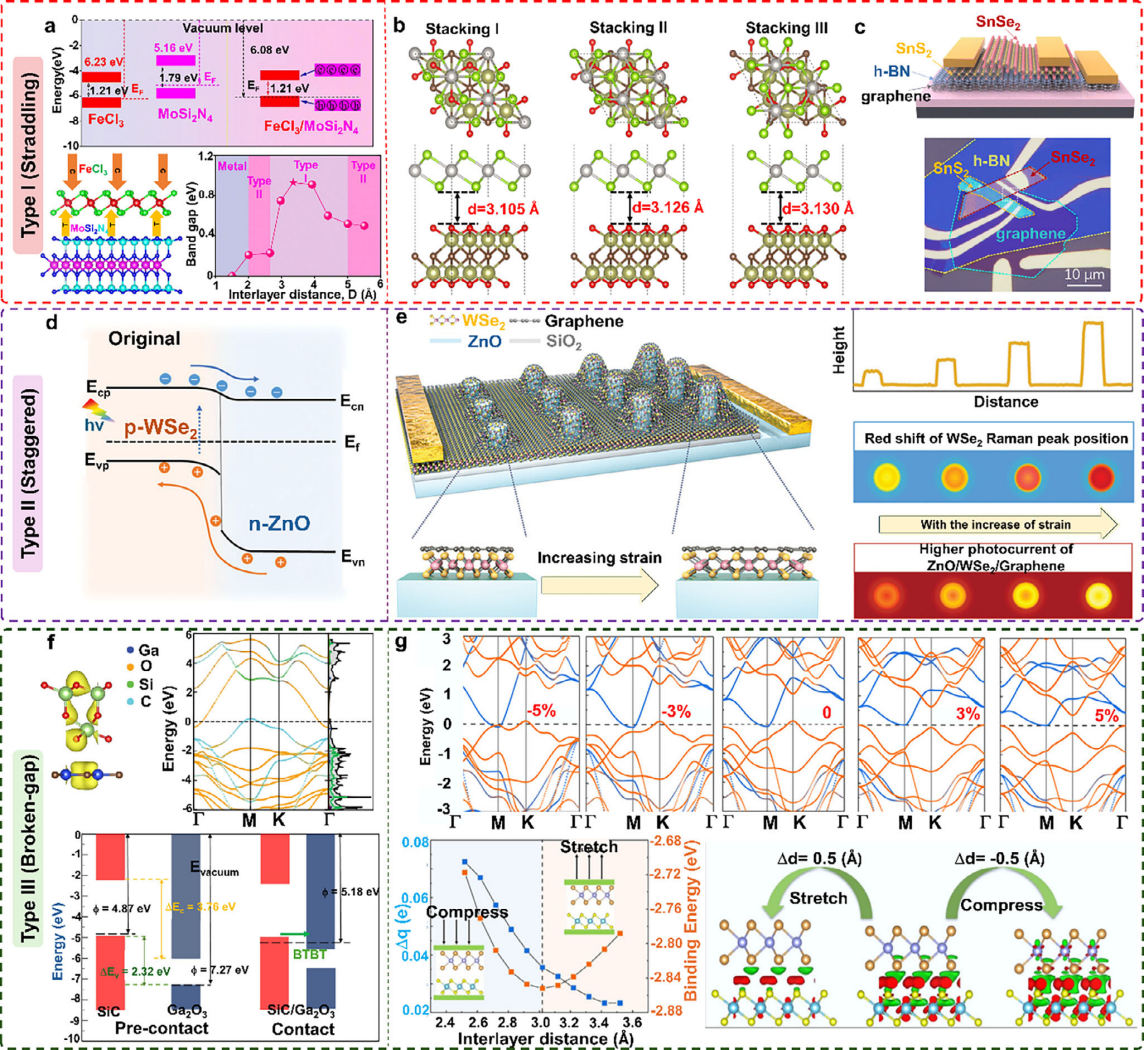
Strain engineered in different types of heterostructures. (a) Band alignment of FeCl_3_/MoSi_2_N_4_ vdWH before and after forming the heterostructure. Reproduced with permission [85]. Copyright 2025, RSC. (b) Schematic model of applied strain and changing interlayer distance, which modulates the bandgap. Top and side views of three different stacking configurations of PtSe_2_/Hf_2_CO_2_ heterostructure. Reproduced with permission [93]. Copyright 2023, RSC. (c) A straddling SnSe_2_/SnS_2_/h‐BN/graphene vdW tunneling heterostructure device. Reproduced with permission [96]. Copyright 2024, RSC. (d) Schematic of the band diagram of ZnO/WSe_2_ heterostructure. (e) Design of ZnO/WSe_2_/Graphene vdW heterostructure with gradient strain modulation. Reproduced with permission [99]. Copyright 2024, Wiley. The higher ZnO nanorods follow the stronger stretching of 2D materials with Raman mapping illustrations, (f) Electron localisation function of Ga_2_O_3_/SiC heterostructure and type III band alignment before and after forming the heterostructure (Φ refers to the work‐function of the material). Reproduced with permission [104]. Copyright 2024, Springer Nature. (g) Band structures of WTe_2_/HfS_2_ vdWH under various biaxial strains. Reproduced with permission [105]. Copyright 2019, ACS. Variation of charge transfer and binding energy versus the interlayer distance under applied strain.

Heterostructures are formed through the combination of two distinct materials, resulting in local heterojunctions at their interfaces [33, 88, 89]. The electronic properties are predominantly influenced by the alignment of band structures at the binding interface, and they are categorised into three types based on band alignment: Type I (straddling gap) (Figure 5a), Type II (staggered gap) (Figure 5d), and Type III (broken gap) (Figure 5f) [90, 93].

The bandgap, electron affinity and work function are the three principal material properties that define heterostructure type, with band offsets governing band alignment in thermal equilibrium via built‐in potentials that induce band bending and discontinuities at valence band maxima or conduction band minima [91].

The difference in electron affinity can be used to determine the discontinuity of the conduction band, as per Anderson's rule. The difference in electron affinity can provide information about the conduction band's discontinuities:

(1) $$\Delta E_{c\;}=\chi_{1}-\chi_{2}$$

Furthermore, the valence band discontinuity can be determined using:

(2) $$\Delta E_{v}=\left(\chi_{1}+\;Eg_{1}\right)-\left(\chi_{2}+\;Eg_{2}\right)$$

where *∆E_c_* and *∆E_v_* are conduction band offset, and valence band offset, respectively. The bandgap and electron affinity for the semiconductors 1 and 2 are represented by Eg_1_, Eg_2,_ and χ_1_, χ_2_, respectively.

The built‐in potential, which is a crucial aspect of the heterojunction's band structure, is established by the interface position of the Fermi levels E_f1_ and E_f2_. As previously discussed, the built‐in potential on both sides of the interface caused band bending to the point where the Fermi levels in two semiconductors finally aligned to form a constant, flat pattern. Consequently, the barrier resulting from the built‐in potential can be provided by:

(3) $$\varphi_{\mathrm{bi}}=E_{f1}-E_{f2}=E_{g}-(\varphi_{1}+\varphi_{2})\;$$

where φ_bi_ is the built‐in potential. E_f1_ and E_f2_ denote the initial Fermi levels of semiconductors 1 and 2, respectively. φ_1_ and φ_2_ correspond to the energy differences between the Fermi level and the respective band edges for each semiconductor.

Type I heterojunctions are characterised by band alignment in which the conduction band minimum and valence band maximum of one material are both located within the bandgap of an adjacent material. This configuration provides quantum well structures that confine charge carriers close together, which makes recombination more efficient [90, 91]. Computational simulations have been the main factor in the recent progress of strain engineering for type I heterostructures, even though experimental work is still in its early stages [90, 91]. For example, first‐principles calculations have been used to study how in‐plane strain and external electric field affect the electronic properties of type I FeCl_3_/MoSi_2_N_4_ heterostructures (Figure 5a) [85]. These studies show that applying compressive strain (ε = −4%) causes a linear decrease in the bandgap, leading the material to change from a semiconductor to a metallic state. In contrast, when tensile strain is applied (1 – 3%), the bandgap significantly increases, going from 0.89 to 0.22 eV. The study also reveals transitions between semiconductor and metallic behavior, as well as between type I and type II heterojunctions [83]. Similar strain‐engineered bandgap modulation has been observed in 2D vertical vdW type I PtSe_2_/Hf_2_CO_2_ heterostructures (Figure 5b), where three distinct stacking configurations offer different advantages for use in LEDs and other optoelectronic devices [93]. Inspired by the strong optoelectronic performance of BN‐TMD heterostructures, forming strained BN‐MS_2_ heterostructures also shows great potential for improving flexible nanodevice performance [60]. Although practical applications of type I‐based electronic devices are still underexplored, promising results have been reported for type I heterostructures integrated on flexible substrates. For instance, 2D vdW type I heterostructures, such as WSe_2_‐MoTe_2_ demonstrate the potential to efficiently and quickly collect light energy, with energy transfer from monolayer WSe_2_ to monolayer MoTe_2_ being almost perfect (∼97%) and extremely quick (∼180 fs) [93]. In another study, a highly efficient photodiode, constructed from type I SnSe_2_/SnS_2_ has also been reported. The device can detect light from ultraviolet to visible wavelengths and has an outstanding responsivity of 37.5 A/W (Figure 5c) [96].

Type II heterojunctions, in contrast, exhibit staggered band alignment, resulting in the conduction and valence bands being offset, thereby spatially separating photogenerated electrons and holes at distinct interfaces [95]. This configuration extends carrier lifetimes by reducing recombination, making type II heterojunctions especially useful for photovoltaic and sensing devices [96]. Gradient strain, when applied to type II ZnO/WSe_2_ vdW heterojunctions (Figure 5d), represents a potent method for tuning optoelectronic properties. As shown in Figure 5e, by adjusting the height of ZnO nanorods, followed by increasing the strain from 1.3 to 4.0%. This built‐in potential at the ZnO/WSe_2_ interface subsequently facilitates the separation of photogenerated carriers [99]. Furthermore, strain‐modulated band engineering has also been explored in Black Phosphorus/MoS_2_ systems, where the band alignment can dynamically shift from type II to type I under strain. Additionally, a semiconductor‐to‐semimetal‐to‐metal transition has been discovered in MoTe_2_/MoS_2_ [100]. These findings underscore the potential of strain engineering as a powerful tool for tuning the electronic behavior of 2D vdW heterostructures and for uncovering the underlying mechanisms governing their functional properties [99, 100, 101].

Type III heterojunctions are characterised by a pronounced band misalignment, wherein the conduction band of one constituent material resides beneath the valence band of its counterpart. This arrangement creates a “broken gap” that promotes band‐to‐band tunneling [103]. This unique transport mechanism is advantageous for specialised electronic and optoelectronic applications. For example, tunneling field‐effect transistor designs based on type III broken‐gap Ga_2_O_3_/SiC vdW heterostructures have demonstrated robust band alignment under both vertical and biaxial strain, indicating strong resilience to mechanical perturbation (Figure 5f) [104]. The transition between type III and type II band alignments is particularly valuable for designing multivalued logic devices. Figure 5g illustrates how the band structures of WTe_2_/HfS_2_ heterostructures change under different biaxial strains, with the K and Γ points shifting under tensile and compressive strain. This causes the band structure to transform from type III to type II alignment under tensile biaxial strain [105]. Low‐dimensional heterostructures show potential for future nanoelectronic applications in a post‐silicon world. Computational studies have explored the structural and electronic properties of type III systems, such as MoTe_2_/ZrS_2_, by looking at different stacking arrangements, layer thicknesses, and the effects of strain and external electric fields [106]. These studies highlight how strain engineering can be used to carefully control band alignment and transport properties in 2D heterostructures (Table 1).

**TABLE 1:** Summary of strain‐induced bandgap modulation in 2D vdW heterostructures.

2D Heterostructure	Strain type	Initial state (0% Strain)	Maximum attainable effect	References
FeCl_3_/MoSi_2_N_4_	Biaxial strain	Type‐I	Substantial modulation of the indirect bandgap and electronic configuration	[85]
PtSe_2_/Hf_2_CO_2_	Biaxial strain	Type‐I	Direct transition in band alignment from Type‐I to Type‐II	[93]
ZnO/WSe_2_/ Graphene	Biaxial strain	Type II	Strain‐induced narrowing of the WSe_2_ bandgap, which optimises energy barriers and exponentially boosts EQE up to 35.3%	[99]
Ga_2_O_3_/SiC	Biaxial strain	Type‐III	Significant enlargement of the tunneling window and enhancement of band‐to‐band tunneling (BTBT)	[104]
WTe_2_/HfS_2_	Biaxial strain	Type‐III	Effective modulation of the broken‐gap characteristics and band electronic features	[105]

#### 2.3.2 Electronic Properties under Strain

**FIGURE 6:**
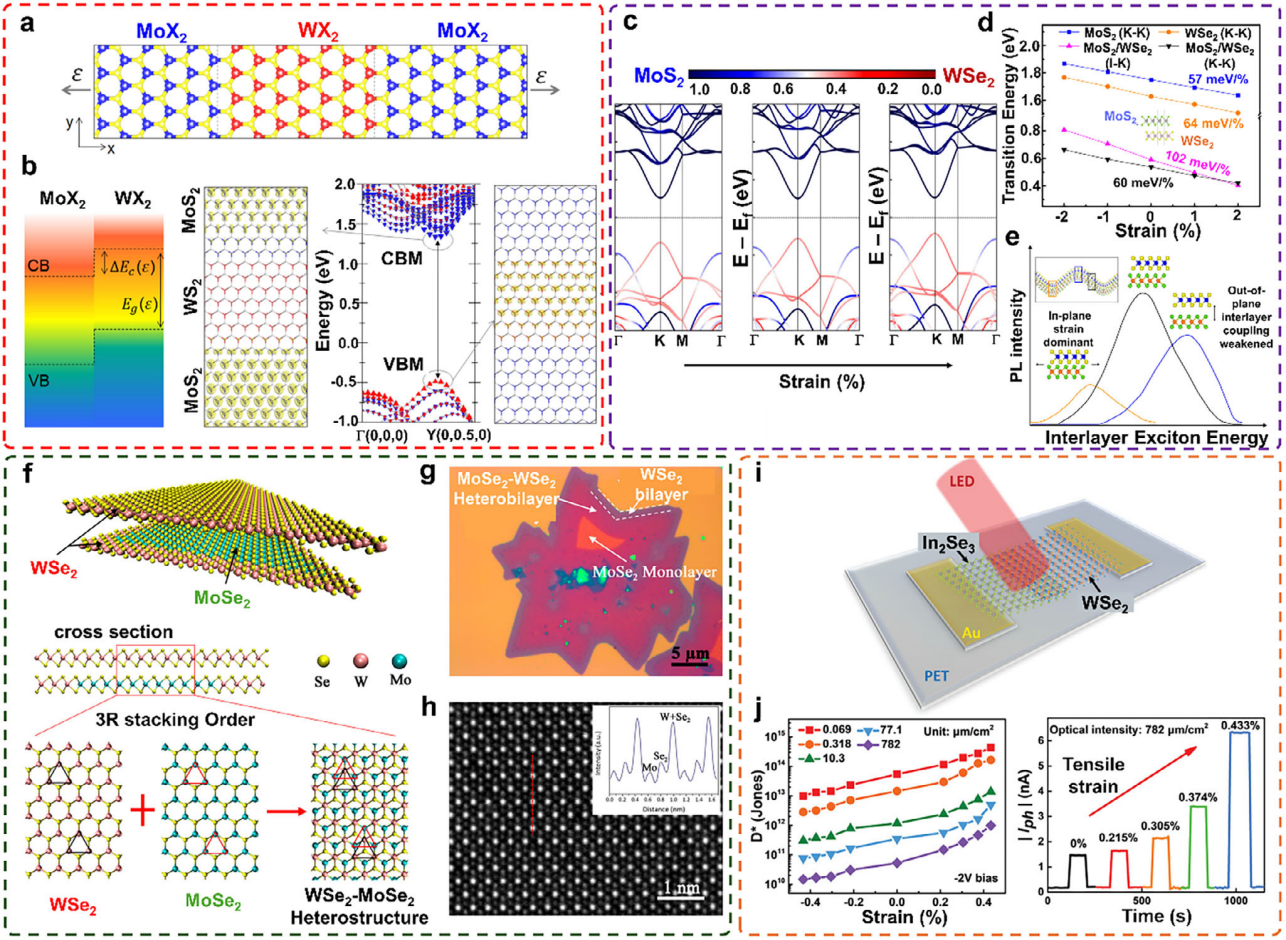
a) Apply strain to a lateral type II MoX_2_/WX_2_ (X = S, Se, Te) heterostructure with a zigzag interface when it experiences an orthogonal uniaxial strain in the armchair direction. Reproduced with permission [114]. Copyright 2017, IOP Publishing. (b) Electronic band structure of lateral unstrained heterostructures with the conduction band minimum and the valence band maximum. Reproduced with permission [114]. Copyright 2017, IOP Publishing. The bidirectional arrow signifies the direct K‐K bandgap. (c) DFT calculation of the modulated band structure of MoS_2_/WSe_2_ heterostructures under different in‐plane strain values, (d) Electronic transition energy of MoS_2_ (K‐K), WSe_2_ (K‐K), MoS_2_/WSe_2_ (Γ‐K), MoS_2_/WSe_2_ (K‐K) as a function of strain, (e) The interlayer exciton PL intensity due to the competition between in‐plane strain and out‐of‐plane interlayer coupling. Reproduced with permission [116]. Copyright 2021, ACS. (f) Schematic of strain‐induced electronic structure change in WSe_2_/MoSe_2_ heterostructure with 3R stacking orientation, (g) The optical image of MoSe_2_‐WSe_2_ heterobilayer, (h) Z‐contrast image of the WSe_2_/MoSe_2_ heterobilayer region. Reproduced with permission [81]. Copyright 2016, ACS. The lowest contrast corresponds to one Mo atom in MoSe_2_, the medium contrast to two Se atoms in WSe_2_, and the highest contrast exhibits the overlap of one W atom in WSe_2_ and two Se atoms in MoS_2_. (i) Schematic illustration of α‐In_2_Se_3_/WSe_2_ photodetector induced by using the piezo‐phototronic effect under red LED illumination. Reproduced with permission, (j) The optical power intensity and photoresponse as a function of strain applied under light illumination. Reproduced with permission [117]. Copyright 2021, Wiley.

The electronic characteristics of 2D heterostructures are notably affected by mechanical strain, thereby facilitating dynamic modulation of their band structures and providing novel control over their electronic properties. As previously discussed, strain engineering in multilayer heterojunctions can significantly modify bandgap energies. Generally, tensile strain tends to diminish bandgaps, potentially inducing semiconductor‐to‐metal transitions under considerable deformation. For example, the MoSSe/WSSe heterostructure exhibits that the bandgap decreases with increasing tensile strain, and at sufficiently high tensile strain, it ultimately undergoes a transition to a metallic state [107]. Additionally, strain has been shown to improve carrier mobility within specific 2D materials. For instance, the hole mobility in 2D B_2_Se_3_ exhibited a substantial increase, from 59.45 to 36 044.72 cm^2^ V^−^^1^ s^−^^1^, when subjected to a 4% strain, a result of diminished hole‐phonon coupling and the flattening of energy bands [108]. This fundamental mechanism is supported by a growing body of recent literature demonstrating extraordinary mobility enhancements in various 2D semiconductor systems. Recent breakthroughs have highlighted how precise structural modulation and interface engineering can effectively suppress scattering mechanisms, thereby achieving record‐high carrier mobilities that push the operational limits of next‐generation field‐effect transistors [109]. Furthermore, advanced strain engineering paradigms continue to be successfully implemented to create highly efficient charge transport channels, significantly elevating macroscopic device performance and stability [109, 110]. Strain is also crucial for adjusting band alignment in heterostructures such as WSe_2_/Black phosphorus (BP), where the transition orientation can induce a transition between type I and type II configurations [111]. The charge transfer pathways in 2D‐integrated heterostructures have shown considerable stability when subjected to mechanical strain. For instance, MoS_2_‐WSe_2_ heterostructures sustain effective interlayer electron transport despite alterations in band offset caused by strain [112]. Investigations into the electronic band structures of zigzag‐intefaced MoX_2_/WX_2_ heterostructures, containing a total of twenty MoX_2_ and WX_2_ units per supercell, have provided foundational insights into how uniaxial strain influences band alignment and optoelectronic behavior (Figure 6a,b) [114]. These studies underscore the robustness of charge transport mechanisms in strained 2D systems and highlight the potential of strain engineering for fine‐tuning interfacial electronic properties.

Strain engineering also enables precise modulation of contact resistance and tunneling probabilities at heterointerfaces, offering substantial performance enhancements in 2D‐integrated heterostructures. In certain heterostructure systems, performance exhibits up to a 100% enhancement in tunneling efficiency under optimised strain conditions, making these materials highly promising for next‐generation flexible electronic and optoelectronic devices [115]. Combined theoretical and experimental approaches, such as density functional theory (DFT), have demonstrated that strain modification of *Γ‐K* interlayer excitons in MoS_2_/WSe_2_ wrinkled heterobilayers yields a deformation potential constant of ∼107 meV/% uniaxial strain, nearly twice that observed for intralayer excitons in individual monolayers (Figure 6c–e) [116]. Further studies have revealed strong electronic coupling between atomic layers in stacked vdW heterostructures, with strain‐induced changes in electronic structure clearly observed (Figure 6f–h) [81]. The bandgaps of stacked MoSe_2_/WSe_2_ heterostructures are lower than the bandgap of individual components. The output performance of the α‐In_2_Se_3_/WSe_2_ heterostructure photodetector is modified under tensile strain, exhibiting an approximately 18‐fold enhancement compared to the strain‐free condition (Figure 6i,j). By integrating piezoelectricity with photoexcitation, the photocurrent (*I*_ph_) may attain 164 nA under 782 µW cm^−2^ illumination and 0.433% tensile strain, demonstrating about a 210‐fold enhancement compared to the I_ph_ under low illumination intensity (0.069 which shows around 210 times improvement in comparison to the I_ph_ under low illumination intensity (0.069 µW cm^−2^) and strain‐free state [117]. A summary of the strain‐induced modifications on the electronic properties of 2D heterostructures is presented in Table 2.

**TABLE 2:** Summary of strain‐induced modifications on the electronic properties of 2D heterostructures.

2D Heterostructure	Strain type	Maximum attainable effect (Electronic properties)	References
MoSe_2_/WSe_2_	Biaxial	Direct‐to‐indirect bandgap transitions and modulated interlayer orbital couplings.	[81]
MoS_2_/WSe_2_	Uniaxial strain	Modulated exciton dynamics, enabling highly tunable interlayer Γ‐K exciton transitions.	[116]
MoX_2_/WX_2_ (X = S, Se, Te)	Uniaxial strain	Tuning spatial carrier confinement and interfacial barriers.	[114]
α‐In_2_Se_3_/WSe_2_	Biaxial strain	Piezo‐potential induced band bending, optimising the interfacial driving force for carrier separation	[117]

#### 2.3.3 Optical Properties under Strain

**FIGURE 7:**
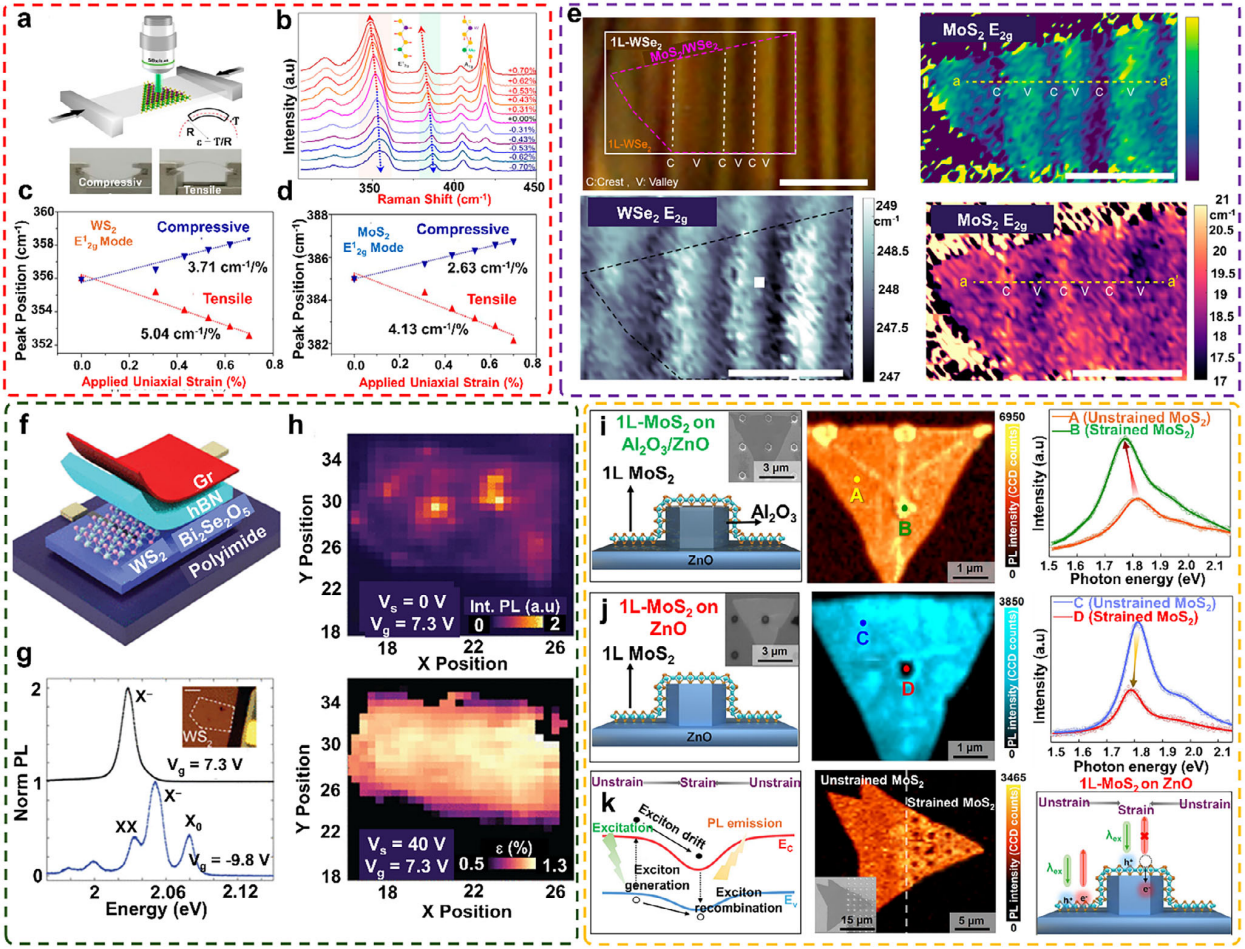
Optical properties of 2D heterostructures under strain. (a) Schematic illustrates the bending setup designed to strain the MoS_2_/WS_2_ heterostructures, (b) The Raman spectra of the heterostructure under strain from 0% to +0.70% (toward red, tensile) and −0.70% (toward blue, compressive), The change in the Raman in‐plane E_12g_ phonon modes of (c) WS_2_ and (d) MoS_2_. Reproduced with permission [119]. Copyright 2017, ACS. (e) The photoluminescence spectra of the vertical wrinkled MoS_2_/WSe_2_ heterobilayer on a prestretched elastomeric substrate_._ Reproduced with permission [116]. Copyright 2021, ACS. (f) Schematic of top‐gated strain‐tuned vdW device WS_2_/h‐BN/graphene is assembled on a flexible polyimide substrate, (g) PL spectra of monolayer WS_2_ device at different top gate voltages (−9.8 and 7.3 V), (h) Spatial mapping of PL intensity over the entire spectral range of devices. Reproduced with permission [120]. Copyright 2025, ACS. (i,j) The schematic, scanning PL maps, and PL spectra of 1L‐MoS_2_ on the 10 nm Al_2_O_3_‐coated ZnO NRAs, MoS_2_ on the ZnO NRAs, respectively, and (k) The proposed mechanism and decreased PL intensity of 1L‐MoS_2_ on ZnO. Reproduced with permission [22]. Copyright 2019, ACS.

The strain effect in 2D‐integrated heterostructures also influences the lattice vibration and crystal orientation, resulting in the change of optical properties such as photoluminescence (PL) and Raman shift [118]. These variations arise from strain‐induced modifications in atomic configurations and energy levels, which directly affect bandgap energies and light emission characteristics. Applying mechanical strain to vdW heterostructures offers a versatile, reversible knob for tailoring their photophysical behavior. A complementary picture emerges in epitaxial MoS_2_/WS_2_ stacks, where tensile strain strengthens WS_2_ emission while compressive strain favours MoS_2_. This result mirrors direct‐to‐indirect gap crossovers in the constituent layers and demonstrates how interlayer coupling redistributes optical weight across the spectrum (Figure 7a–d) [119]. In the study of momentum‐indirect *Γ–K* interlayer excitons of MoS_2_/WSe_2_, the wrinkled heterobilayers with uniaxial strain modulate the transition energy by an unusually large 107 meV per % (Figure 7e) [116]. This phenomenon exhibited twice the sensitivity of intralayer excitons. Thereby indicating the accompanying non‐monotonic photoluminescence intensity reflects a competition between in‐plane band‐gap renormalisation and out‐of‐plane coupling under strain modulation [116]. In the study by John Cenker et al., the weak strain transfer of graphite‐hBN for high‐quality vdW devices was studied. Efficient strain transmission through orthohomic substrates like CrSBr or Bi_2_SeO_5_ allows vdW devices to endure several per cent deformation at cryogenic temperatures without slippage. These advantages facilitate gate‐tunable WS_2_ monolayers whose excitonic peaks exhibit a linear red‐shift while their circular polarisation and oscillator strength evolve simultaneously (Figure 7f–h) [120]. Another study, based on the idea of mixed‐dimensional MoS_2_/ZnO heterostructures, reveals that nanostructured tensile strain (∼0.6%) reduces the interfacial barrier, enhances mobility of charge transfer, and consequently diminishes MoS_2_ photoluminescence by over 50% in comparison to unstrained areas (Figure 7i–k) [22]. These findings collectively demonstrate that strain engineering in 2D heterostructures not only alters excitonic resonances but also redistributes carrier mobility and modulates interfacial dynamics. These phenomena offer significant adaptability in a wide range of wavelengths, bringing a robust avenue for broadband optoelectronic sensing devices.

In addition to standard linear optical characterisations such as Raman and photoluminescence (PL), investigating second‐harmonic generation (SHG) provides a highly sensitive, non‐destructive method for evaluating the optical response of 2D layered materials under strain [121]. Recent empirical studies have successfully utilised SHG to explicitly quantify and manipulate strain‐induced phenomena, rather than relying solely on theoretical symmetry analysis. For instance, polarisation‐resolved SHG has been employed to directly and quantitatively map local strain vectors and folding angles in deformed atomically thin WS_2_, offering spatial resolution and precision that exceed conventional linear spectroscopy [122, 123]. A recent study demonstrated an up to 100‐fold enhancement of SHG intensity in a 64°‐stacked WSe_2_/WS_2_ heterobilayer by the coupling of interlayer interactions with local nanoscale strain [121]. Furthermore, dynamic strain modulation, such as optomechanical tuning in asymmetric Janus MoSSe/MoS_2_ heterostructures, has been proven to actively control wavelength‐dependent SHG anisotropy. These practical demonstrations underscore that SHG is not a basic structural probe, but a critical characterisation tool for advancing the design of strain‐tunable optoelectronic and piezotronic sensor applications [122, 124]. Table 3 summarises the strain‐induced modifications on the optical properties of 2D heterostructures.

**TABLE 3:** Summary of strain‐induced modifications on the optical properties of 2D heterostructures.

2D Heterostructure	Strain type	Maximum attainable effect	References
MoS_2_/WS_2_	Uniaxial strain	Continuous tuning of the WS_2_/MoS_2_ PL ratio, monotonically increasing under tension and decreasing under compression	[119]
Wrinkled MoS_2_/WSe_2_	Periodic strain	Giant spectral tuning of Γ‐K excitons (∼107 meV/%) accompanied by nonmonotonic PL intensity modulation	[116]
WS_2_/Bi_2_SeO_5_	Uniaxial strain	Robust exciton/trion tuning and a significant ∼30% reduction in the Degree of Circular Polarisation (DOCP)	[120]
MoS_2_/ZnO nanorod	Localised biaxial strain	>50% PL quenching at the interface due to a reduced potential barrier and enhanced charge transfer	[22]

#### 2.3.4 Electromechanical Properties under Strain

Two‐dimensional heterostructures have garnered significant attention in advanced materials research owing to their capacity to modulate electromechanical characteristics under applied strain. This section consolidates recent progress in the geometrical effect, disconnection mechanisms, tunneling effects, and crack generation in 2D heterostructures subjected to strain. The findings indicate that strain significantly alters the electrical characteristics, and the displacements can substantially influence the atomic structure and mechanical characteristics [125].

##### 2.3.4.1 Geometrical Effect

**FIGURE 8:**
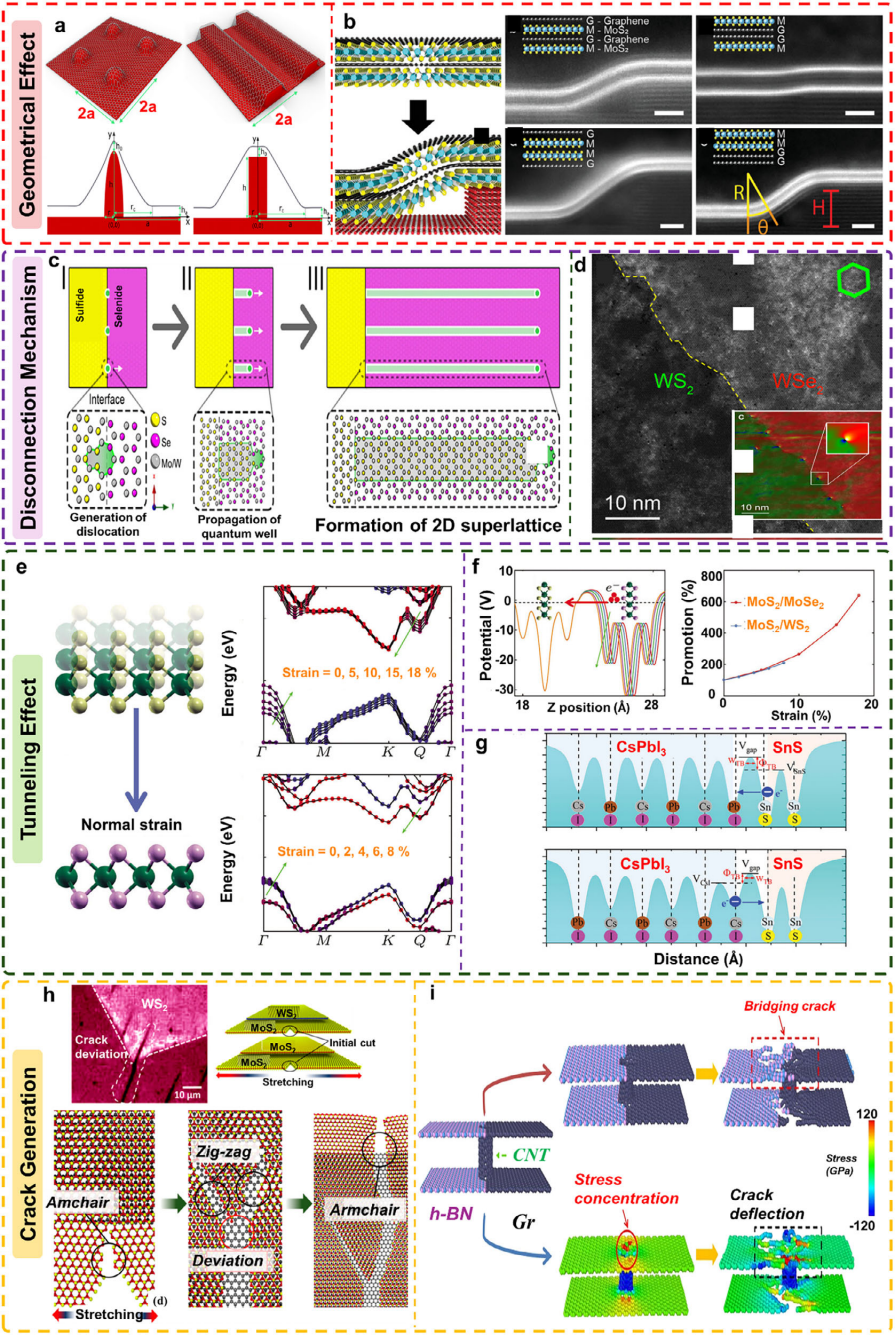
Electromechanical properties of 2D heterostructures under strain. (a) The schematic illustrates two structural configurations of nanopillars and rectangular one‐dimensional substrate grating with their vertical cross sectional view, respectively. Reproduced with permission [126]. Copyright 2024, RSC. (b) The bending of four‐layer 2D heterostructures composed of two graphene (G) and two MoS_2_ (M) layers, Reproduced with permission [127]. Copyright 2021, Wiley. (c) The formation of periodic dislocation arrays of 2D quantum‐well superlattice in the WSe_2_/WS_2_ lateral heterostructure. (d) STEM image of a WSe_2_/WS_2_ lateral interface with the expitaxial interface is highlighted by the yellow dashed line. Reproduced with permission [130]. Copyright 2018, AAAS. (e) The normal strain applied in MoS_2_/MoSe_2_ and the band structures of MoS_2_/MoSe_2_ and MoS_2_/WS_2_ heterostructures under strains. (f) Electrostatic potentials of the MoS_2_/MoSe_2_ heterostructures under different strains and the tunneling probability promotions in the heterostructures. Reproduced with permission [133]. Copyright 2020, IOP Publishing. (g) Average effective potential profile along the z‐direction for the asymmetric strain introduced at PbI_2_/SnS and CsPbI_3_/SnS interfaces, Reproduced with permission [118]. Copyright 2019, Wiley. (h) Optical micrograph of WS_2_/MoS_2_ heterostructure (monolayer) showing deviation of crack at the interface. Reproduced with permission [138]. Copyright 2018, ACS. (i) The crack propagation and strain distribution of G/BN heterostructures in the presence of CNT. Reproduced with permission [139]. Copyright 2024, Elsevier.

Geometrical effects are fundamental processes that govern how strain distribution, how materials respond to strain, and performance in 2D heterostructures subjected to mechanical deformation. The geometry of the substrate is essential for controlling strain localisation and amplification. For instance, the nanopatterned surfaces, such as nanopillar arrays and 1D gratings, produce distinct strain patterns and mechanical responses [26, 28]. When a 2D material undergoes mechanical strain, changes in its length and cross sectional area result in variations in electrical resistance. The precise identification of strain direction and location within the material is facilitated by incorporating specific geometric patterns, such as mesh designs, triangles, or hexagons, into the two‐dimensional substrate. Consequently, these geometric configurations significantly enhance the gauge factor, thereby improving the sensitivity and overall effectiveness of geometrical‐based sensors. The critical aspect ratio, defined as the ratio of height to period, significantly impacts the wetting/dewetting transition and the resulting strain distribution, as illustrated in Figure 8a [126]. Furthermore, the mechanical behavior of heterostructures is significantly influenced by interfacial geometry, particularly in relation to bending stiffness and deformability. The unique geometric configuration, characterised by misaligned interfaces due to interlayer twisting or heterointerface creation, results in a significant decrease in interfacial arrangements (Figure 8b) [127]. Geometric limitations generated by the substrate, such as curvature, wrinkles, and surface corrugations, provide spatially heterogeneous strain fields. Thus, these attributes enable the development of strain‐engineered systems with tunable electrical, optical, and mechanical properties spanning nanometer to micrometre scales [124, 126, 127].

##### 2.3.4.2 Disconnection Mechanism

The electrical disconnection mechanism in 2D‐integrated heterostructures under strain is a crucial phenomenon. This process involves the formation or movement of lattice defects, especially dislocations and disconnections, at heterointerfaces. When stacked heterostructures experience significant strain, interlayer sliding can occur, thereby diminishing the overlap of the flakes and increasing interlayer resistance [130]. If the overlap area gets much smaller, some areas may completely lose electrical contact. This can cause localised circuit disruptions, making the entire device more resistant [131]. This method can make electrical responses that are very sensitive to strain, especially in strain sensors made of 2D materials. The disconnection mechanism has been demonstrated in WSe_2_/WS_2_ lateral heterostructures [130]. At the heterointerface, misfit dislocations with pentagon‐heptagon (5|7) pairs show up because the lattices cannot match up perfectly (around 4%) (Figure 8c,d) [130]. When the strain is enough, these dislocations not only stay at the interface but also “climb” deep into the WSe_2_ layer. This creates ultra‐thin WS_2_ quantum well strips that are around 1.19 nm wide and go deep into the WSe_2_ layer [130]. This is an example of a common strain‐controlled disconnection mechanism in 2D heterostructures. The disconnection process demonstrates how strain engineering can reduce strain and help create new nanostructures with adjustable electrical and optical properties in 2D‐integrated heterostructures.

##### 2.3.4.3 Tunneling Effect

The tunneling effect in 2D heterostructure materials is a phenomenon where electrons pass through the potential barrier between two atomically thin layers due to quantum effects [106, 132]. When strain is applied, especially compressive or tensile strain perpendicular to the plane of the layers, the tunneling barrier and tunneling probability at the heterostructure interface can be strongly modulated by strain [106]. Density functional theory (DFT) calculations have shown that reducing the interlayer spacing in WSe_2_/CrSe_2_ vdW heterostructures results in a reduction of the tunneling barrier height and width, thereby increasing the tunneling probability, which can reach 100% at approximately −20% strain [113]. Similarly, in MoTe_2_/ZrS_2_ heterostructure, compressive strain enhances charge transfer and tunneling current by decreasing the interlayer spacing, which subsequently enhances band‐to‐band tunneling (BTBT) (Figure 8e) [133]. First‐principles calculations on transition‐metal dichalcogenide stacks indicate that a reduction of the vdW gap by up to 33% maintains a direct BTBT path while enhancing the tunneling probability by over forty‐five times and stabilising the interface (Figure 8f) [133]. In the perovskite‐CsPbI_3_/monolayer‐SnS system, biaxial strain reduces the barrier to approximately 1 Å and increases the transmission probability to nearly 12%, effectively doubling the value of the relaxed junction (Figure 8g) [118]. The results obtained validate that strain engineering facilitates precise regulation of tunneling barriers, permitting the tuning of carrier transmission from almost obstructed to nearly totally transparent without the incorporation of chemical defects. This forms the foundation for high‐performance strain‐gated tunneling transistors, quantum memories, and ultrasensitive 2D strain sensors.

##### 2.3.4.4 Crack Generation

The initiation and spread of cracks are a significant mechanical phenomenon affected by strain in 2D‐integrated heterostructures [134, 135]. Crack initiation in these heterostructures is significantly influenced by biaxial strain resulting from mismatched thermal expansion coefficients between layers (e.g., MoS_2_/WS_2_) or by crystalline lattice defects at domain boundaries, including vacancies, dislocations, Stone–Wales defects, and grain boundaries [136, 137]. Under tensile strain, fractures typically advance along less robust crystal planes or areas with diminished van der Waals bonding (Figure 8h) [138]. Studies of MoS_2_ have shown that fractures tend to propagate along the zigzag orientation, a phenomenon attributed to its lower surface energy [125]. During this process, cracks can spread either parallel or perpendicular to the applied strain, leading to the formation of areas with different crystal structures on either side of the crack [125, 139]. Recent studies indicate that regulating crack propagation by the adjustment of twist angles and the reinforcement with carbon nanotubes can markedly improve the mechanical characteristics and stability of graphene/hexagonal boron nitride (G‐BN)/carbon nanotube (CNT) heterostructures (Figure 8i) [139]. Comprehending the principles of crack initiation and propagation in 2D‐integrated heterostructures is crucial for the development of ultrasensitive strain sensors and flexible electronic devices. Table 4 summarises the strain‐induced modifications on the electromechanical properties of 2D heterostructures.

**TABLE 4:** Summary of strain‐induced modification on the electromechanical properties of 2D heterostructures.

Property	2D Heterostructure	Strain technique	Maximum attainable effect	References
Geometrical	MoS_2_/Graphene	Out‐of‐plane bending	Predictable geometric response via precisely tuned bending stiffness	[127]
Disconnection	WS_2_/WSe_2_	Lattice mismatch	Formation of sub‐2‐nm lateral quantum wells	[130]
Tunneling	MoS_2_/MoSe_2_	Out‐of‐plane bending	Giant promotion of direct band‐to‐band tunneling (BTBT) probability	[133]
	CsPbI_3_/SnS	Interface mismatch	Precise modulation of the interfacial tunneling barrier (TB)	[118]
Crack Generation	WS_2_/MoS_2_	Out‐of‐plane bending	Effective crack deflection and delayed fracture	[138]
	Graphene/hBN	Out‐of‐plane bending	Reversible microcrack opening/closing; Ultra‐high sensitivity (Giant GF > 10^3^)	[139]

## 3 Fabrication and Strain Engineering Techniques

The synthesis techniques for 2D heterostructure materials play a pivotal role in the formation of 2D‐integrated heterostructure devices. Factors such as cleanliness, defects, number of layers, thickness, and surface binding energy must also be tightly controlled. Currently, several studies have addressed techniques for creating 2D heterostructures; however, discussions on strain engineering in 2D‐integrated heterostructures remain limited. In this section, we will summarise the common techniques for synthesising heterostructures, followed by a detailed discussion of the methods for inducing strain in heterostructures.

### 3.1 Fabrication Techniques

**FIGURE 9:**
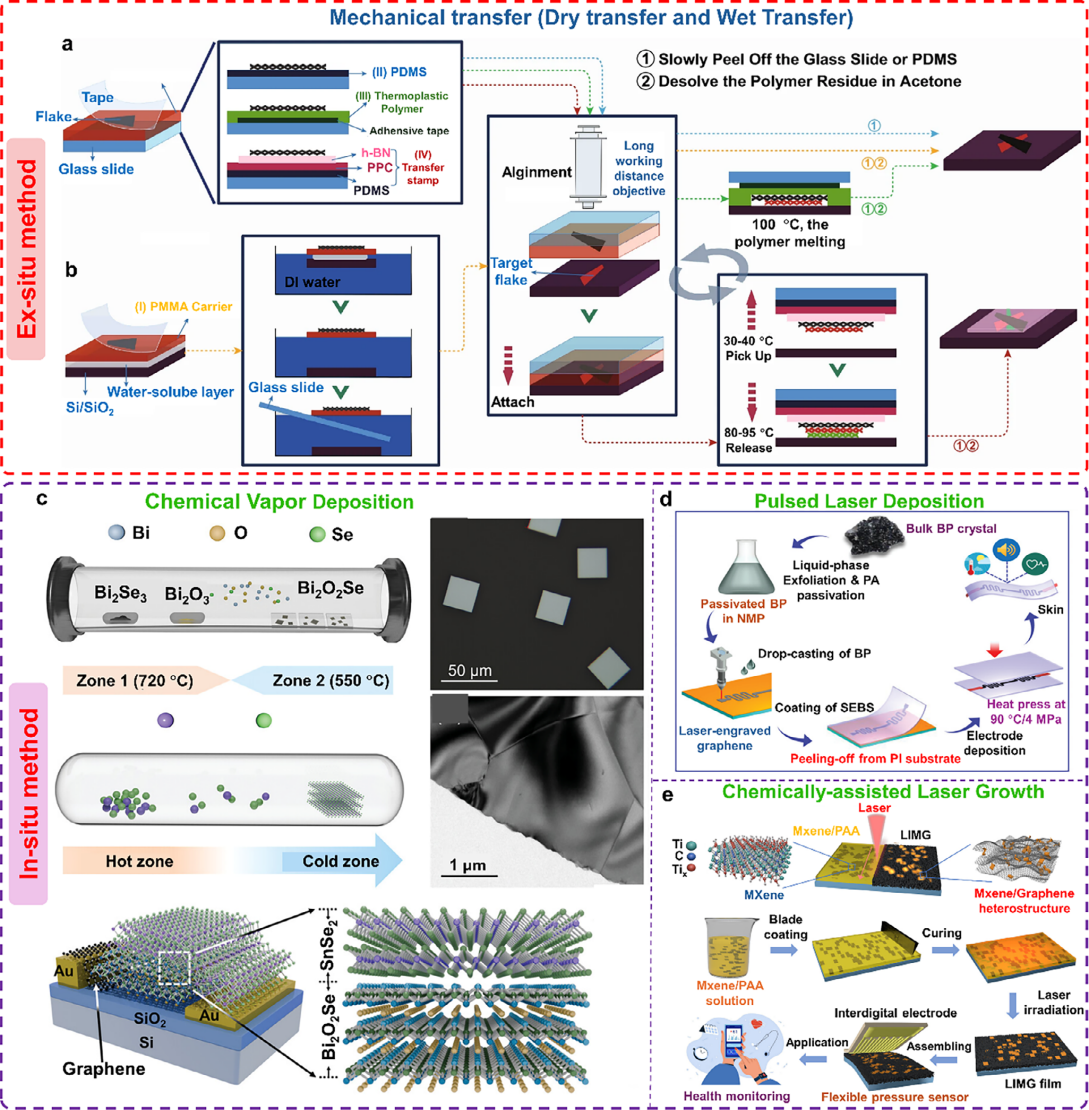
Heterostructure fabrication techniques. Ex situ method. (a,b) The deterministic (dry and wet) transfer methods to form vdW heterostructures. Reproduced with permission [140]. Copyright 2019, Springer Nature. The yellow, blue, green, and red dashed lines correspond to the process of transfer using four types of carriers (I to IV). At the end, the bare heterostructures or multilayer heterostructures will be fabricated. In situ method. (c) Schematic diagram illustrating the chemical vapour deposition method of the Bi_2_O_2_Se/SnSe_2_ heterojunction device. Reproduced with permission [142]. Copyright 2024, Wiley. (d) Schematic diagram of exfoliation of BP and fabrication of BP@LEG based hybrid sensor. Reproduced with permission [148]. Copyright 2021, Wiley. (e) Schematic diagram of laser‐induced MXene/graphene heterostructure (LIMG) film. Reproduced with permission [149]. Copyright 2024, ACS.

To advance the understanding and practical application of 2D materials, a comprehensive review of synthesis techniques for producing high‐quality 2D materials is essential. There are many key challenges, including achieving high crystallinity, maintaining atomic‐level interface cleanliness, and controlling the lateral dimensions of synthesised heterostructures. Currently, there are two primary approaches for fabricating 2D‐integrated heterostructures: ex situ and in situ methods. The ex situ method involves mechanical exfoliation or pre‐fabrication of individual 2D layers, which are subsequently transferred and manually stacked onto target substrates, using either dry or wet transfer techniques (Figure 9a,b) [140]. Although ex situ assembly is straightforward, it often encounters challenges such as limited control over flake dimensions, inconsistency in layer thickness, and susceptibility to contamination. These challenges primarily arise from the reliance on relatively weak vdW interactions during the construction of ultrathin 2D layers [97].

In contrast, the in situ method directly produces ultra‐thin films and heterostructures by advanced deposition techniques such as chemical vapor deposition (CVD) and molecular beam epitaxy (MBE). The CVD technique allows for the scalable growth of uniform 2D layers with controllable thickness and high crystallinity. Recent research on Bi_2_O_2_Se nanosheets formed using dual‐zone temperature has shown that the heterostructures can be modulated by precisely tuning growth conditions, source and substrate temperatures, and carrier gas flows (Figure 9c) [142, 143]. Additionally, alternative in situ methods, such as MBE and other epitaxial techniques, are constrained by several challenges, including complex recipe optimisation, substrate selection and variation, which restrict large‐area high‐quality growth [11]. Furthermore, the requirement for high processing temperatures and ultra‐clean environments contributes to high costs and hinders mass production. Recently, twisted 2D van der Waals (vdW) heterostructures constitute a captivating class of materials, in which strain‐dependent physical properties and functional performance exhibit remarkable tunability and novel phenomena [144]. Beyond traditional mechanical stacking, which often introduces interfacial contamination and wrinkles, chemical vapour deposition (CVD) has emerged as a crucial approach for the direct synthesis of these twisted heterostructures [144]. However, CVD growth typically favours thermodynamically stable 0° or 60° stacking configurations due to substantial energy barriers. Recent studies have successfully overcome these thermodynamic limitations by employing innovative kinetic control strategies to synthesise twisted transition metal dichalcogenides (TMDCs) spanning a complete 0° to 120° range. For instance, researchers have utilised space‐confined reconfiguring nucleation assisted by NaCl, employed tilted substrates to induce heterogeneous precursor concentration gradients, and applied strategic gas‐flow perturbations to drive homo‐site or hetero‐site nucleation [145, 146, 147]. This direct bottom‐up fabrication yields pristine, polymer‐free interfaces that are highly critical for effective twist and strain engineering, as they allow for strong interfacial mechanical coupling, localised lattice reconstructions, and the formation of strong moiré potentials. Despite these great improvements in fabrication, associated synthesis challenges remain. While the overall density and yield of CVD‐grown twisted TMDCs have improved significantly, achieving deterministic, localised control over specific small twist angles remains difficult due to the complex, dynamic, and unpredictable gas‐phase environments inherent to CVD systems.

To overcome these limitations, alternative synthesis techniques have been explored. Pulsed laser deposition (PLD) offers a rapid, maskless, and high‐precision method to grow 2D materials and heterostructures at ambient pressure. For instance, sensitive strain and temperature sensors have been exemplified by laser‐engraved graphene combined with black phosphorus nanoflakes (Figure 9d) [148]. This PLD technique leverages high‐energy laser pulses to photothermally or photochemically process. This enables precise control over film morphology and properties without the need for complex vacuum systems [148]. Another promising approach is chemically assisted laser growth, where chemical additives combined with laser irradiation facilitate localised thermochemical synthesis of heterostructures. For instance, MXene/graphene composites can be synthesised by a one‐step laser thermochemical process (Figure 9e) [147, 149]. This approach leverages laser‐induced heating to facilitate the interaction between nanosheets and polymer matrices. The findings result in forming hierarchical 2D/3D architectures with enhanced functional properties suitable for flexible sensors. Moreover, supercritical carbon dioxide (sc‐CO_2_) has been employed for the large‐scale production of 2D materials such as WS_2_ nanosheets. The sc‐CO_2_‐assisted exfoliation process not only provides high‐efficiency layer separation from bulk crystals but also allows partial conversion to heterostructures such as WS_2_/WO_3_.H_2_O by controlled oxidation, offering a facile and scalable route under mild conditions [150].

In summary, a range of synthesis techniques with distinct advantages, CVD for wafer‐scale uniformity, pulsed laser deposition for rapid and maskless growth, chemically assisted laser processes for hierarchical heterostructures, and liquid‐phase exfoliation for scalable material production, address the fundamental challenges of fabricating 2D‐integrated heterostructures. Their merits advance the production of high‐quality, large‐area, and clean 2D heterostructures critical for future applications in optoelectronics and flexible sensing devices.

### 3.2 Strain Engineering Techniques

This section provides a concise overview of various techniques employed to apply strain to 2D materials, opening up new avenues for applying strain to 2D‐integrated heterostructures. This section is divided into two parts based on the nature of the position distribution of applied strain: (1) homogeneous strain with biaxial and uniaxial applied, and (2) inhomogeneous localised strain.

#### 3.2.1 Homogeneous Strain

Homogeneous strain in 2D materials and 2D‐integrated heterostructures can be categorised into biaxial and uniaxial types, depending on the number of principal strain orientations and the method of force application. Each method possesses distinct advantages in optimising the optical and electrical properties of the material.

##### 3.2.1.1 Homogeneous Biaxial Strain

**FIGURE 10:**
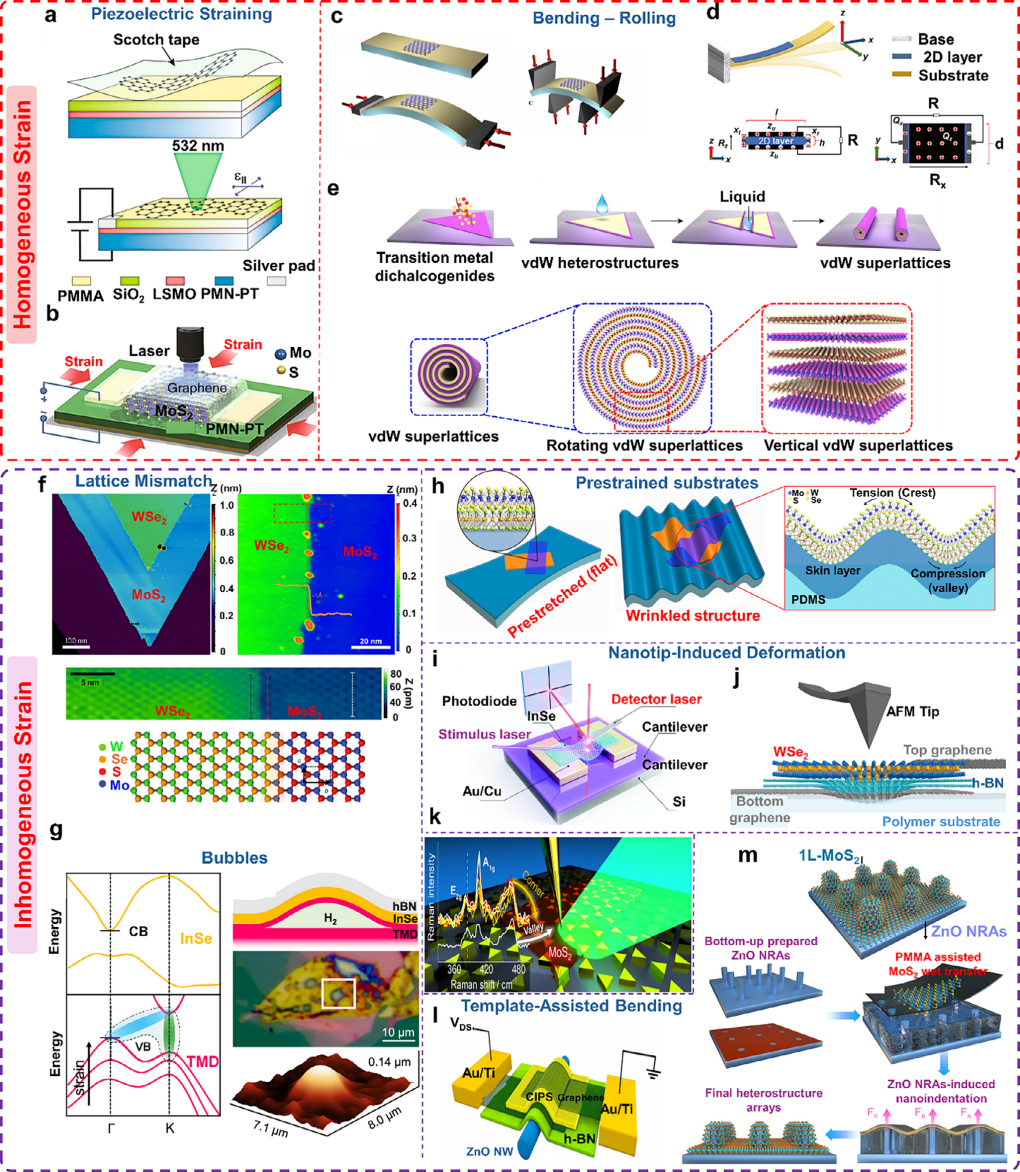
Homogeneous Strain. Piezoelectric straining. (a) In‐plane biaxial strain of Graphene/PMN‐PT substrate. Reproduced with permission [152]. Copyright 2010, ACS. (b) Schematic diagram of MoS_2_ sandwiched between a piezoelectric PMN‐PT substrate and a graphene layer serving as an electrode. Reproduced with permission [153]. Copyright 2013, ACS. Bending technique. (c) two‐point bending. Reproduced with permission [157]. Copyright 2009, APS. (d) Cantilever bending. Reproduced with permission [158]. Copyright 2021, ACS. Rolling technique, (e) Schematic illustration of the fabrication process of roll‐up to form a vertically stacked heterobilayer vdW heterostructure. Reproduced with permission [159]. Copyright 2021, Springer Nature. Inhomogeneous strain. (f) STM images of the lattice mismatch WSe_2_‐MoS_2_ lateral heterostructures. Reproduced with permission [160]. Copyright 2018, Springer Nature. Bubbles. (g) Heterostructure InSe/TMD bubbles. Reproduced with permission [98]. Copyright 2025, ACS. Prestrained Substrates. (h) Fabrication of wrinkled MoS_2_/WSe_2_ heterostructures on a prestretched elastomeric substrate. Reproduced with permission [116]. Copyright 2021, ACS. Nanotip‐Induced Deformation. (i) Schematic illustrates the suspended InSe film between the two gold electrodes. Reproduced with permission [161]. Copyright 2021, RSC. (j) Schematic of the vertical heterostructure consisting of top graphene/WSe_2_/h‐BN/bottom graphene on the polymer substrate with localised strain‐driven form AFM tip. Reproduced with permission [162]. Copyright 2021, AAAS. (k) Localised strain in a MoS_2_/Au heterostructure conducted with Tip‐Enhanced Raman Spectroscopy. Reproduced with permission [163]. Copyright 2017, ACS. Template‐Assisted Bending. (l) Schematic illustrates the 1D(ZnO NWs)/2D(CuInP_2_S_6_) mixed‐dimensional heterostructures device with a curved structure. Reproduced with permission [164]. Copyright 2024, ACS. (m) Fabrication of periodically strain‐engineered 1L‐MoS_2_/ZnO heterostructure arrays. Reproduced with permission [22]. Copyright 2019, ACS.

Biaxial strain refers to the deformation that occurs when a homogeneous distortion is simultaneously applied along two orthogonal in‐plane axes. This results in the uniform expansion or contraction of the material's lattice in both directions. In 2D heterostructures, biaxial strain is predominantly imposed by piezoelectric substrates [151]. Under the application of an electric field, these substrates generate predictable and reversible in‐plane stresses. Consequently, providing an electrically switchable mechanism to apply uniform, reversible strain on vdW stacks without the mechanical complexity of bending stages [152]. A pioneering instance is Fei et al., who applied a tunable biaxial stress to graphene by using Pb(Mg_1/3_Nb_2/3_)O_3_–PbTiO_3_ (PMN‐PT) substrate. By using the electro‐mechanical approach, they can manipulate the biaxial compressive or tensile strain to graphene with 20V applied can correspond to a strain of 0.0028% (Figure 10a) [152]. In another study of Hui et al., who laminated a trilayer MoS_2_/graphene stack onto a PMN‐PT single crystal. The out‐of‐plane polarisation of the substrate caused about 0.2% biaxial compression and changed the direct gap by an unprecedented 300 meV per 1% strain while still allowing light to pass through the graphene electrode (Figure 10b) [153]. In summary, the same “piezo‐backplane” idea is now widely used for heterostructures [152]. Homogeneous train engineering in 2D materials emphasises that piezoelectric substrates offer vector control, rapid switching, and compatibility with cryogenic or in situ spectroscopies, making them the technique of choice for programmable band‐structure and exciton‐physics experiments in 2D heterostructures [153, 154].

##### 3.2.1.2 Homogeneous Uniaxial Strain

Uniaxial strain in two‐dimensional materials is characterised by homogeneous deformation where stress or strain is exerted along a singular major direction, while other directions are either permitted to deform or constrained. Homogeneous uniaxial strain is the most prevalent and extensively utilised technique for strain engineering in 2D‐integrated heterostructures. The procedures necessitate external force and substrate for the application and regulation of the strain process.

##### 3.2.1.3 Bending

A controlled uniaxial force can be exerted on a 2D material via bending in homogeneous strain applications by transferring the 2D material onto a flexible substrate and subsequently bending the substrate [165, 166]. When the flexible substrate is bent, one side is compressed while the opposite is stretched, producing a consistent uniaxial strain across the 2D materials that is oriented perpendicular to the bending axis [167, 168]. The approaches can be categorised into two main types based on apparatus: two‐point or four‐point bending systems (Figure 10c) [157] and cantilever bending systems (Figure 10d) [158, 169].

The uniaxial strain induced on the surface of a flexible substrate during two‐point bending is expressed by the equation [170]:

(4) $$\varepsilon =\frac{t}{2R}$$

where *t* represents the substrate thickness and *R* denotes the radius of curvature (assuming R is significantly larger than t) [157]. For a cantilever of length *L*, clamped at one end and subjected to a point load at the free end, the uniaxial strain on the substrate surface is [167, 169]:

(5) $$\varepsilon =\frac{3t\delta }{2L^{2}}\left(1\;-\;\frac{x}{L}\right)$$

where *x* represents the distance measured from the cantilever's fixed end, while *δ* indicates the amount of deflection at the free end. This equation assumes that δ remains small enough so that the largest slope is significantly less than 1.

Based on the idea, the generated strain in 2D heterostructures brings an effective approach to boosting performance compared to individual 2D materials [169, 170]. Two‐point bending systems are typically applied for large 2D sheets (lateral size ≥ 100 µm), whereas cantilever systems are appropriate for smaller sheets (≤ 10 µm) [157]. These methodologies are extensively employed in research on graphene and TMDCs [169, 171]. The greatest possible uniaxial strain of the 2D sheet varies between 0.5% and 3.8%, contingent upon its strength and the equipment utilised [171, 173].

##### 3.2.1.4 Rolling

Rolling techniques are frequently utilised in graphene nanofabrication, generating uniaxial strain orthogonal to the rolling direction [159]. Due to the remarkable mechanical strength of 2D materials, this method can induce either tensile or compressive strains, contingent upon the pre‐straining. The typical maximum compressive strains attained are approximately −0.3%, surpassing those achieved by most other approaches. Recent research has shown that compressive strains over 5% may be achieved in graphene using a self‐rolled‐up membrane platform, wherein graphene co‐rolls with strained dielectric films, allowing for strain modulation through the curvature of the rolled structure [175, 176]. Bei Zhao et al. employed a direct method to induce strain in high‐order vdW superlattices by rolling up SnS_2_/WSe_2_ heterostructures (Figure 10e) [159]. This approach can be expanded to develop varied 2D/2D vdW superlattices and a broad spectrum of multi‐dimensional vdW structures, including 3D/2D (Al_2_O_3_/SnS_2_/WSe_2_), and 1D/2D (Ag nanowires/WSe_2_), among others.

#### 3.2.2 Inhomogeneous Localised Strain

In addition to uniform in‐plane strains, where deformation is evenly distributed across the entire 2D crystal lattice, localised inhomogeneous deformations can be introduced through targeted strain engineering techniques. In this section, the following methods of generating localised inhomogeneous strain, lattice mismatch, bubble formation, prestrained substrates, nanotip‐induced deformation, and template‐assisted bending will be described in detail.

##### 3.2.2.1 Lattice Mismatch

Epitaxial growth is an advantageous method for acquiring extensive, high‐quality 2D materials and integrated heterostructures. The intrinsic lattice mismatch during this procedure induces strain in the fabricated materials and heterostructure. Wang et al. compared the Raman spectra of strain‐free with two approaches, including mechanically transferred MoS_2_ and CVD‐grown monolayer MoS_2_ on h‐BN and SiO_2_/Si substrates [143, 179]. Their findings indicated that MoS_2_ grown on SiO_2_/Si substrates exhibited 0.45% biaxial tensile strain, while MoS_2_ grown on h‐BN had a Raman spectrum similar to unstrained MoS_2_, indicating much less lattice strain. Similarly, in lateral heterostructures of 2D materials, strain is inevitably introduced during epitaxial growth due to lattice mismatch between the two materials. For instance, Zhang et al. discovered the lattice constants of WSe_2_ and MoS_2_ are 3.282 and 3.160 Å, respectively, resulting in a lattice mismatch of 3.8%.  Moreover, the study also revealed about 1.76% tensile strain and attributed discrepancies between strain and lattice mismatch to a high density of dislocations at the interface (Figure 10f) [160]. A similar result was also reported by Li et al., with maximum tensile and compressive strains in the MoS_2_ region of MoS_2_‐WSe_2_ lateral heterostructures as 1.59 ± 0.25% and 1.1 ± 0.18%, respectively [180]. While these lateral heterostructures are examples of incoherent epitaxy, Xie et al. recently reported coherent superlattices with dislocation‐free, isotropic lattice structure and triangular symmetry [181]. In such coherent superlattices, the fully matched lattice constants mean that WS_2_ and WSe_2_ (with the larger lattice constant) experience tensile and compressive strain, respectively, depending on the supercell dimensions [181]. To minimize the total energy per WSe_2_ unit in a strained WSe_2_ monolayer on SiO_2_, the compressive strain in WSe_2_ relaxes, resulting in the formation of a rippled structure [182, 183, 184].

##### 3.2.2.2 Bubbles

Due to the vdW forces between 2D materials and the substrate, water and hydrocarbon molecules adsorbed on 2D materials aggregate together during the assembly process, resulting in bubble formation at the interface [185]. The bubbles cause surface fluctuations in 2D materials, which induce strain at the interface. The dimensions, morphology, and internal pressure of these bubbles result from the interplay between the vdW forces and the elastic energy necessary for the deformation of 2D material [186]. The strain distribution in nanobubbles demonstrates a universal scaling tendency, with the peak strain generally located at the center of the bubbles and decreasing toward the edges [187, 188]. Grigorieva et al. examined strained bubbles in vdW heterostructures for emitter applications by transferring monolayer MoS_2_ onto different substrates via wet transfer method. This process resulted in the formation of bubbles at the interface, thereby introducing a smooth and gradient strain of approximately 2% into the monolayer MoS_2_ [187]. The optical microscopy (OM) image and AFM topography of the bubbles at the MoS_2_/h‐BN interface demonstrate the characteristic shape and size distribution of these strain‐inducing features [186, 189, 190].

There are two factors that characterise the shape of the bubble: R and h, which stand for the bubble's radius and its maximum height, respectively. The aspect ratio h/R is defined using the following formula:

(6) $$\frac{h}{R}=\frac{\pi \gamma }{5C_{1}Y}$$

where *γ* denotes the adhesion energy of 2D materials to the substrate, *C_1_* represents a numerical coefficient, and *Y* signifies Young's modulus of the 2D materials. Consequently, the aspect ratio h/R of a bubble pertains solely to 2D materials and substrates, being unaffected by the bubble itself. The local strain (ε) of monolayer MoS_2_ surrounding a bubble is directly proportional to the aspect ratio *h/R*, which can be articulated as follows:

(7) $$\varepsilon \;∼\;{\left(\frac{h}{R}\right)}^{2}$$

In 2025, Blundo et al. conducted a study on the selective application of strain to MS_2_ (M = Mo or W) monolayers within InSe/MS_2_ heterostructures. The findings demonstrate that these heterostructures induced a substantial photoluminescence amplification of the highly tunable yet weakly emitting InSe, exceeding two orders of magnitude (Figure 10g) [96]. These optimised qualities confer exceptional electrical and transport characteristics, substantially strengthening the prospects for various optoelectronic applications.

##### 3.2.2.3 Prestrained Substrates

Strain engineering in 2D materials via prestrained flexible elastomeric substrates offers a straightforward and widely applicable technique for inducing inhomogeneous local strains [129, 191, 193]. The working principle involves initially stretching an elastomeric substrate, followed by the deposition of 2D material sheets (exfoliated MoS_2_) onto the prestrained substrate, and then releasing the applied load [193]. A dispersion of wrinkles in the 2D material sample is generated due to localised buckling and debonding, which releases substantial strain energy [129, 191]. In the study of Cho et al., the heterobilayer was prepared on an elastometric substrate (PDMS) by using a heat and strain‐release mechanism, a periodic wrinkled MoS_2_/WSe_2_ heterostructure was formed (Figure 10h) [116].

##### 3.2.2.4 Nanotip‐Induced Deformation

AFM is an important tool for characterising the physical properties of 2D materials [194]. High‐resolution AFM not only enables precise mapping of surface morphology, but also allows for the application of z‐axis pressure to 2D materials through direct tip‐sample contact, thus inducing strain due to local deformation caused by the tip [195]. Preliminary investigations using AFM have demonstrated the ability to induce isotropic compressive and tensile strains in monolayer MoS_2_ by applying forces at different positions of the MoS_2_ flake through the AFM tip [196]. The results revealed that the monolayer MoS_2_ flake became concave when the tip was placed in the central region (corresponding to compressive strain). Moreover, it became convex when the tip was located near the edges (corresponding to tensile strain) [196], and the local strain ε can be expressed as:

(8) $$\varepsilon =\frac{F}{A\;\times \;E}$$

where *F* is the applied force, conducted by the AFM tip, *A* is the tip's cross sectional area, and *E* is the Young's modulus of monolayer MoS_2_.

Recent advancements have increasingly integrated localised strain with various 2D materials, leading to the emergence of the flexo‐photoelectronic effect. This effect arises from the synergistic coupling between flexoelectricity and photoelectricity, thereby leveraging the benefits of strain and light interaction. For instance, isotropic bending of n‐type InSe and p‐type WSe_2_ semiconductors on flexible substrates has demonstrated the ability to localise charge carriers through inhomogeneous strain, offering a promising route toward 2D neuromorphic circuit applications (Figure 10i) [161]. Building on this concept, tunneling emitter devices based on graphene/h‐BN/WSe_2_ heterostructure have utilised AFM to induce localised tensile strain, enabling precise modulation of tunneling characteristics (Figure 10j). These discoveries introduce a novel method utilising electrically driven strain‐induced mechanisms for 2D‐integrated heterostructures [162].

Building upon this foundation, tip‐enhanced Raman spectroscopy (TERS), which combines AFM and Raman measurements, has been extensively used to monitor strain distribution in strained 2D materials [158, 197, 198]. With subnanometer accuracy, the tip metal is excited by the laser to produce localised surface plasmon resonance, a strong electromagnetic field is generated between the tip and the sample surface, greatly improving the intensity of Raman signals and the image resolution [198]. Recently, the detection of spatially localised strain has reached nanoscale resolution in strained monolayer MoS_2_ systems (∼2.3 nm) [199], trilayer MoS_2_ (∼25 nm) [163], and monolayer WSe_2_ (∼15 nm) [200]. In 2D/plasmonic heterostructures, the built‐in strain MoS_2_‐Au nanotriangle heterojunction arises from the conformal bending of the ultrathin MoS_2_ flake over the patterned gold nanotriangles (Figure 10k) [163]. As demonstrated in studies where frequency shifts of the E_2g_ mode corresponding to 1.4% biaxial strain were detected with 25 nm spatial resolution [163]. In this technique, areas of maximal topographic curvature generally correlate with areas of maximum local strain, according to concurrent AFM measurements.

##### 3.2.2.5 Template‐Assisted Bending

In the template‐assisted bending approach, this method induces a highly localised, inhomogeneous uniaxial bending strain by conformally draping a 2D heterostructure over an underlying nanotemplate. In 2024, Wu et al. demonstrated the ZnO nanowires as a flexible bending medium to induce large deformations and significant nonuniform strain in 2D vdW heterostructures CIPS/h‐BN/graphene (Figure 10l) [201]. The strain‐induced piezoelectric effect can be generated because of the weak vdW forces at the interface, which facilitates the significant enhancement of open‐circuit voltage and zero‐biased responsivity (7.62‐fold) compared to a flat heterostructure. The study first demonstrated the successful modulation of photodetection in 2D heterostructures by using controlled curvature media. Another approach also favours the prepatterned substrate before forming heterostructures. Baishan Liu et al. demonstrated a strategy to construct mixed‐dimensional heterostructure arrays with periodically strain‐engineered vdWs interfaces using monolayer MoS_2_ (1L‐MoS_2_)/ZnO (Figure 10m) [22]. The results indicate that inhomogeneous built‐in strain gradient at the heterointerfaces ranging from 0 to 0.6% tensile, also providing a variety of degrees of freedom in tuning vdW interface performance. These approaches can achieve strains up to several per cent and allow for dynamic tuning and reversible mechanical reconfiguration of heterogeneous deformation patterns [191]. A comprehensive comparison of current strain techniques, including their achievable strain, scalability, and specific applications for 2D heterostructures, is presented in Table 5. Consequently, the decision to prioritise either specific synthesis methods or strain engineering techniques depends on a range of factors, including target strain limits, substrate compatibility, and fabrication costs. Therefore, it remains highly challenging to identify a single, universally promising approach for commercial sensor platforms.

**TABLE 5:** Summary of strain‐induced techniques for 2D heterostructures.

Strain technique	2D Heterostructure	Advantages	Disadvantages	Achievable Strain	Scalability	Applications	References
**Piezoelectric Actuators**	MoS_2_/Graphene	Precise, reversible in situ biaxial strain tuning via applied voltage	Expensive substrates; complex electrodes; limited by piezo coefficient	Small (< 0.2% ‐0.5%)	Low	High‐resolution dynamic optoelectronic tuning	[153]
**Cantilever Bending**	2D TMDs/h‐BN on Si substrate	Direct MEMS/NEMS integration; efficient strain transfer	Non‐uniform strain gradient along the beam	Small (depends on curvature)	Moderate	Energy harvesters and electromechanical sensors	[158]
**Rolling**	SnS_2/_WSe_2_	Facile liquid‐induced rolling	Irreversible; static strain fixed by radius	Curvature‐dependent	Moderate	Compact 3D optoelectronics	[159]
**Prestrained Substrates**	MoS_2_/WSe_2_	Induces periodic tensile/compressive strain; ideal for in situ observation	Non‐uniform spatial distribution of strain	Large	Moderate	Investigating interlayer coupling and periodic strain gradients	[116]
**Lattice‐Mismatch**	WSe_2_/MoS_2_	Intrinsic atomic‐level strain	Strains often relax via dislocations	Large ∼2.2% – 3.8%	High (Wafer‐scale CVD)	Monolithic lateral p‐n junctions and integrated optoelectronics	[160]
**Bubbles**	InSe/MS_2_ (M = Mo, W)	Extremely high biaxial strain without tearing	Random microbubble formation; highly non‐uniform strain profile	High (> 4%)	Low	Giant photoluminescence enhancement via band realignment	[98]
**Nanotip‐Induced Deformation**	WSe_2_/hBN/Graphene	Deterministic local strain induction	Requires costly equipment; risk of surface damage	Small (0.16 – 0.21%)	Low (Not scalable)	Creating site‐specific, deterministic single‐photon emitters	[162]
	3L‐MoS_2_/Au	Ultra‐high resolution (< 25 nm) local strain	Geometry‐dependent strain profile; risk of surface damage	High local strain (∼1.4%)	Low	Site‐specific quantum emitters and opto‐mechano‐plasmonic studies	[163]
**Template‐Assisted Bending**	1L‐MoS_2_/ZnO	Periodic, uniform nanoscale strain gradient	Complex nanopillar fabrication (lithography); strain is strictly limited by the pillar geometry	∼0.6% (Biaxial tensile)	High (Wafer‐scale arrays)	High‐density optoelectronic arrays using mixed‐dimensional (2D/1D) systems	[22]
	α‐In_2_Se_3_/β‐InSe on ZnO NWs	Induces giant strain gradients	Spatially non‐uniform (localised along the NW); difficult alignment during flake transfer	High curvature (0.1−1µm^−1^)	Moderate	Enhancing self‐powered photodetection by band alignment tuning	[201]
	CuInP_2_S_6_/ZnO NWs	Induces giant strain gradients	Spatially non‐uniform; difficult alignment during flake transfer	High gradient (up to 4.2 × 10^6^ m^−1^)	Moderate	Enhancing optoelectronics in 2D ferroelectrics	[164]

## 4 Sensing Mechanisms

In this section, we will give a comprehensive review and discussion of strain‐engineered approaches in sensing mechanisms, including working mechanisms and up‐to‐date relevant studies. While currently there are a number of review articles on strain engineering in 2D materials, they lack focused discussion on 2D‐integrated heterostructures, as well as extensive discussion on sensing mechanisms. In addition, we will summarise and mention the advanced coupling effects that are being widely and effectively used in the field of sensing.

### 4.1 Photovoltaic Effect

**FIGURE 11:**
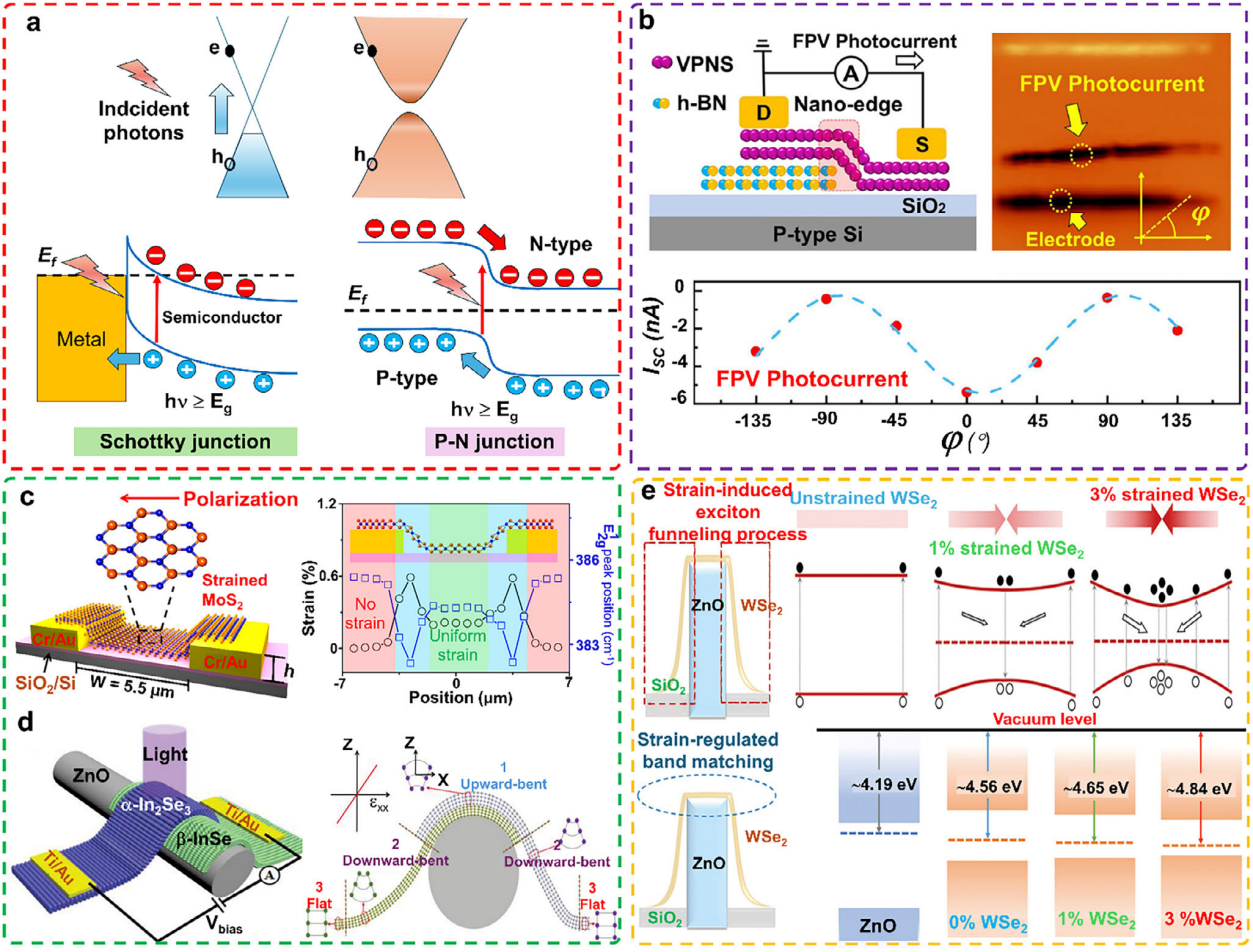
(a) The mechanism of the photovoltaic effect, (b) Schematic illustration the structure of the 2D VPNS/h‐BN device. Reproduced with permission [209]. Copyright 2024, ACS. The short‐circuit photocurrent map of the strained VPNS/h‐BN device with polarisation angle φ = 0° and the dependence of photovoltage current of strained VPNS/h‐BN on the polarisation direction, (c) Schematic diagram of the strained monolayer MoS_2_ device with the strain distributed along the cross section. Reproduced with permission [210]. Copyright 2024, ACS. (d) Schematic illustrates the structure of curved α‐In_2_Se_3_/β‐InSe heterostructures, where strain is generated by modulating the diameter of ZnO nanowires. Reproduced with permission [201]. Copyright 2024, Wiley. The schematic diagram demonstrates different curved states in the heterostructures. (e) The mechanism of the strain‐enhanced optoelectronic performance of ZnO/WSe_2_ heterostructures. The convergence of electron‐hole pairs in WSe_2_ by applied strain. The strain‐modulated band alignment of ZnO/WSe_2_ heterostructure, with the dashed lines demonstrating the Fermi level of ZnO and WSe_2_ at different strain conditions. Reproduced with permission [99]. Copyright 2024, Wiley.

The photovoltaic (PV) effect is the phenomenon whereby materials generate voltage and current when exposed to light [202]. In the field of optical sensing, 2D‐integrated heterostructures play an important role in improving photosensitivity compared to individual 2D materials [203]. The most commonly reported photodetection mechanisms include photoconductive, photogating, photothermal, photo‐bolometric, and photovoltaic effects [202]. These effects have been widely discussed and applied in photodetection based on narrow‐bandgap 2D semiconductor materials [110, 205]. However, the perspective on strain engineering for 2D materials and 2D‐integrated heterostructures in photodetection performance has not yet been thoroughly investigated. In this review section, we will discuss in detail the photovoltaic effect in 2D‐integrated heterostructures, where the strain engineering approach demonstrates evolutions compared to the conventional photovoltaic effect. The fundamental mechanism of the photovoltaic effect can be described in Figure 11a, with conventional photovoltaic devices, where current is generated when an electric field is formed at the p‐n junction, which promotes the separation of electrons and holes [110]. Photodetectors operating based on the photovoltaic effect can be considered photodiodes [206, 207, 208]. More specifically, the formation of vdW heterostructures between p‐type and n‐type semiconductor materials creates a built‐in field p‐n junction, or a Schottky junction is formed between the semiconductor surface and metal (electrodes) [209]. As mentioned in Section 2.3.1, under strain, the band alignment in 2D‐integrated heterostructures can be transformed into different types (I, II, or III), which can affect the transport mechanism of photocharged carriers, leading to adjustments in sensor performance [117, 119].

In recent studies aimed at enhancing bulk photovoltaic effect (BPVE) performance, strain is often applied to BPVE materials to leverage the strain‐induced piezoelectric effect that improves electron‐hole separation process [110, 206, 211]. This BPV effect is achieved through the disruption of inversion symmetry via uniaxial strain in 2D materials. Sun et al. demonstrated systematically strain‐dependent regulation of the photovoltaic effect. The explored strain engineering in phosphorene nanosheets (VPNS) by using h‐BN nanoedge has achieved a BPV coefficient up to 1.3 × 10^−3^ V^−1^ and a polarisation ratio reaching 21.6 (Figure 11b) [209]. Wang et al. investigated the photocurrent increasing multiple times for MoS_2_ material when using in‐plane strain of about 0.2%, achieving a photoresponsivity of 0.1 A/W, much higher than BPVE materials (Figure 11c) [101]. Following up on these ideas, strain engineering in 2D vdW heterostructures was explored by Qi et al. in 2024, using nanowires with different diameters as the bending media in modulating the photodetection of 2D α‐In_2_Se_3_/β‐InSe heterojunctions. These findings provide a new perspective for controlling strain generated in 2D vdW optoelectronic devices (Figure 11d) [201]. Another study inspired by adjusting bending media, the ZnO/WSe_2_/graphene vdW heterostructures with gradient strain were created through dynamic height adjustment of ZnO nanorods (Figure 11e) [99]. According to theoretical calculations, the increasing height of ZnO leads to an increasing gradient strain of WSe_2_, mainly concentrated at the edge. This promotes an increase in the number of photocharged carriers converging at the ZnO/WSe_2_ interface on the top of ZnO nanorods, resulting in adjustments to the photodetection performance of 2D vdW heterostructure devices.

### 4.2 Piezoelectric Effect

The piezoelectric effect is the reversible electromechanical phenomenon whereby certain non‐centrosymmetric crystalline materials generate an electric charge upon the application of mechanical stress (direct piezoelectric effect). Conversely, when subjected to an external electric field (inverse piezoelectric effect), the materials undergo mechanical deformation [212].

The piezoelectric effect in 2D‐integrated heterostructure materials represents a significant advancement in materials research, facilitating accurate regulation of mechanical‐to‐electrical energy conversion at the atomic level [158]. Piezoelectric effect in 2D heterostructures is attributed to the disruption of inversion symmetry in their crystalline architectures, establishing a direct correlation between mechanical deformation and electrical polarisation [213, 214]. In contrast to bulk materials, stacked 2D heterostructures exhibit unique piezoelectric responses attributable to their atomic thickness and adjustable interfacial characteristics. Mechanistically, strain in 2D heterostructure materials affects the electronic band structure, hence affecting the spatial distribution of charge density [38, 215]_._ This generates electric dipole moments, evidenced by quantifiable piezoelectric coefficients, particularly in non‐centrosymmetric 2D materials that accommodate both in‐plane and out‐of‐plane polarisation components [213, 216].

**FIGURE 12:**
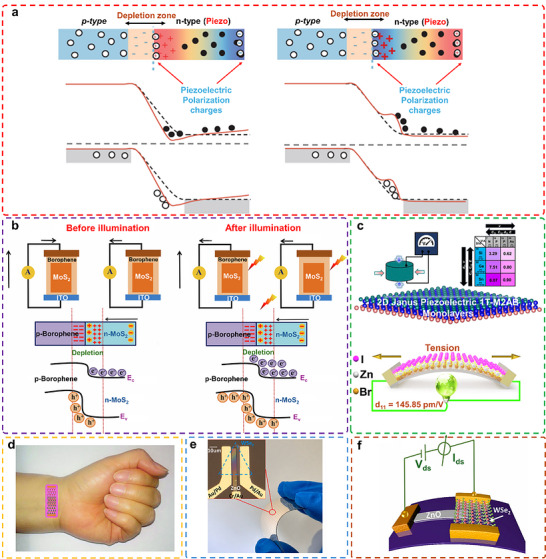
Piezoelectric effect in 2D heterostructure materials. (a) Schematic of the energy band diagram illustrating the effect of piezoelectricity on a p‐n junction. Reproduced with permission [219]. Copyright 2018, Wiley. The bandgap for the n‐type and p‐type is assumed to be about equal. The presence and absence of the piezoelectric effect correspond to the two possible cases. (b) The process of piezoelectric charge generation in Boronphene‐MoS_2_ heterostructures before and after UV‐light illumination. Reproduced with permission [220]. Copyright 2025, Springer Nature. Under light excitation, an increased number of electron‐hole pairs results in improved piezoelectric output performance, allowing Br‐MoS_2_ heterostructures to function as self‐powered UV‐piezo sensors. (c) 2D Janus Piezoelectric 1T‐M2AB Monolayer materials and 2D Janus ZnBrI materials have a large in‐plane piezoelectric coefficient d_11_, up to 145.85 pm/V. Reproduced with permission [222]. Copyright 2024, ACS. (d) A schematic drawing of a portable and wearable piezoelectric adhesive bandage with the WSe_2_/MoS_2_ partial stacked heterostructures on a wrist for monitoring muscle dynamics and arterial repetition. Reproduced with permission [226]. Copyright 2018, RSC. (e) Optical image, and (f) operation schematic of the flexible WSe_2_‐ZnO photodetector under tensile strain. Reproduced with permission [227]. Copyright 2019, Elsevier.

The mechanism underlying the piezoelectric effect in semiconductor heterojunctions is elucidated by the presence of piezoelectric‐induced polarisation charges at the interface of a p‐n junction [217]. The classification of heterojunction types, p‐n junction, consists of two semiconductor regions with an opposite doping type, and is considered a fundamental building block in modern electronics and optoelectronics [218]. Once the strain is induced in the n‐type semiconductor (piezoelectric material), the positive piezopotential induced in the n‐type region close to the junction interface attracts the electrons toward the interface, resulting in the trapping or accumulation of electrons adjacent to the interface. This leads to a dip in the local band profile (Figure 12a) and enhances the electron‐hole recombination, which is a favourable outcome for light‐emitting devices. On the other hand, if the polarity of the induced strain is reversed, the negative piezopotential created in the n‐type region close to the junction interface can repel the electrons away from the interface, resulting in the depletion of electrons adjacent to the interface and thus a shoulder in the local band profile. This may result in the suppression of the electron‐hole recombination rate in the device, which could be detrimental for related optoelectronic applications [216].

High‐performance flexible piezoelectric nanogenerators have been developed using as‐grown borophene‐MoS_2_ nanosheet heterostructures [220]. Remarkably, the heterostructure nanogenerator demonstrates an energy conversion efficiency of 37%, output voltage of 22 V, and current density of 34 µA cm^−2^, outperforming their pristine components by almost ten times [220]. The observed shrinkage of the bandgap upon forming the heterostructure further benefits carrier transport and enhances electromechanical conversion for 2D‐based self‐powered UV sensor devices (Figure 12b) [220]. Furthermore, recent investigations emphasise 2D Janus nanostructures, distinguished by their asymmetric surfaces, as significant advancements in piezoelectric engineering (Figure 12c) [221, 222]. These materials strengthen piezoelectric performance by disrupting structural symmetry. In the study of Wang et al., Janus ZnBrI demonstrates a d_11_ value of 145.85 pm/V, approximately 40 times more than that of 2D 2H‐MoS_2_. This finding highlights the efficacy of asymmetric design in improving electromechanical coupling [221, 223]. Additionally, in another study, the Janus CrOFBr monolayer, which achieves out‐of‐plane piezoelectric strain coefficients d_31_ and d_32_ of 1.21 and 0.63 pm/V, respectively, provides significant insights into the development of 2D piezoelectric materials in the field of nanoelectronics [224]. In the study about the out‐of‐plane piezoelectric effect in α‐In_2_Se_3_/TMD heterostructures, Chen et al. proposed a strategy that shows great piezoelectric enhancement compared with monolayer α‐In_2_Se_3_ [225]. In 2D heterostructure WSe_2_/MoS_2_ atomic layers, wherein a type‐II staggered gap between p‐type WSe_2_ and n‐type MoS_2_ induces considerable electric polarisation. Nanoscale devices have attained output voltages of 0.137 and 0.183 V under tensile pressures of 4 and 8%, respectively (Figure 12d) [226]. Moreover, the gate‐tunability of vdWs heterostructures is widely applied to enhance the performance of various devices. A significant example of manipulating 2D WSe_2_‐1D ZnO vdW interfacial charge broadens applications for next‐generation photodetection and imaging (Figure 12e,f) [225, 227].

### 4.3 Piezoresistive Effect

The piezoresistive effect refers to the alteration of electrical resistivity in a semiconductor or metal when subjected to mechanical stress [228]. The large piezoresistive effects in semiconductors demanded a fundamental theory of physics, where strain in a crystalline solid modifies the lattice constants and reduces the crystal symmetry, leading to significant shifts in the energy band edges [179, 229, 230]. The existing theories were based on the change in energy band structure, including band warping and splitting, where the density of states and lattice symmetry of crystals change under applied strain [231, 232]. The findings result in shifts of bandgap and changes in electron mass under strain that lead to changes in conductivity and carrier mobility [229]. Nowadays, the demand for wearable electronic devices has changed the structure of resistive‐type strain sensors from brittle to a stretchable format, with strain sensors typically composed of active sensing materials combined with flexible and stretchable supporting substrates [230, 231, 232]. Most theoretical models exhibit crystal orientation dependence of band structure, electron energies, and the effective masses of carriers [233]. At the scale of metals or semiconductors, the piezoresistive effect can be observed, while semiconductor materials possess this effect at a much higher level than metals [234]. The resistance (R) of a material is defined as:

(9) $$R=\frac{\rho l}{A}$$

where *ρ* is the electrical resistivity, *l* is the length, and *A* is the cross sectional area of the material [233]. The gauge factor (GF) of a material is demonstrated by:

(10) $$\mathrm{GF}=\frac{\Delta R/R\;}{\varepsilon }$$

where *R* is the original resistance, *ΔR* is the change in resistance, and *ε* is the strain.

The stress (σ) can be inferred from the applied force by Hooke's law for a homogeneous material, as follows:

(11) σ=ε×E

where *E* is the Young's modulus.

**FIGURE 13:**
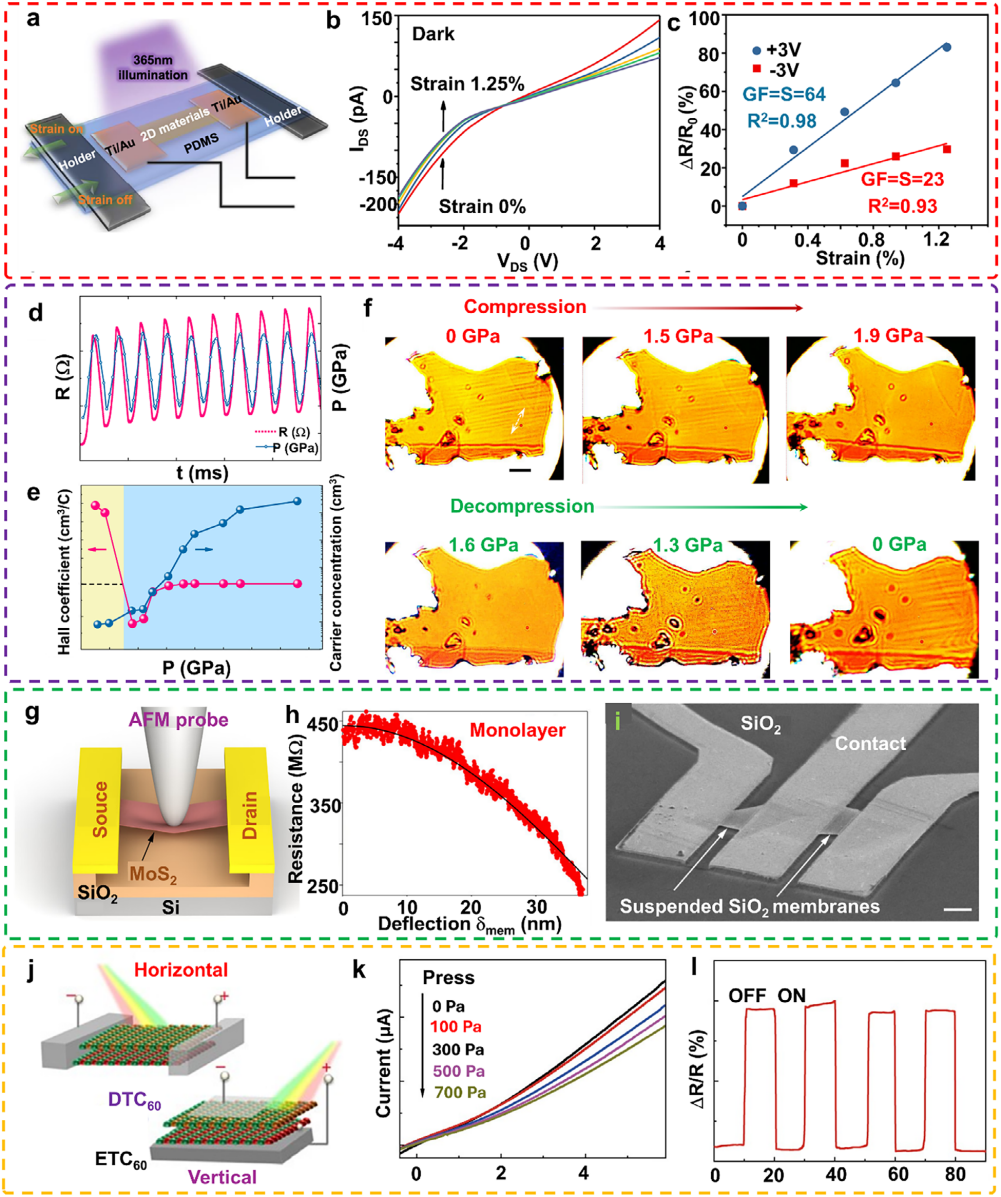
Piezoresistive effect in 2D heterostructure materials. (a) Schematic of the vdW 2D material strain sensor devices. (b) The *I*–*V* curves under various strain conditions were measured in darkness. (c) The variation of ΔR/R_0_ as a function of strain for the sensor measured in darkness. Reproduced with Creative Commons CC BY license [237]. Copyright 2021, Springer Nature. (d) Pressure‐dependent linear dichroism of *β’*‐In_2_Se_3_ with dynamic piezoresistance measurement, (e) Hall coefficient and carrier concentration as a function of pressure measured at room temperature, (f) Micrographs of linear dichroism are taken as pressure increases from 0 GPa to 1.9 GPa, and recovery of the domain contrast upon release of the pressure. Reproduced with Creative Commons CC BY license [238]. Copyright 2023, Springer Nature. (g) The image illustrates strain‐induced bandgap tuning in atomically thin membranes MoS_2_, which exhibit high Young's modulus and fracture strength, (h) The measurement and the corresponding simulation resistive results for a monolayer of MoS_2_, (i) SEM image of MoS_2_ devices with suspended channels and contact electrodes. Scale bar is 1 µm. Reproduced with Creative Commons CC BY‐NC 4.0 [239]. Copyright 2015, ACS. (j) Schematic illustrating the horizontal and vertically stacked ETC_60_/DTC_60_ organic 2D heterostructures for mechanical‐electronic response, (k) Current‐voltage curves with the loading pressure from 0 to 700 Pa, and (l) Resistance changes with the cycle on and off of 100 Pa pressure. Reproduced with Creative Commons CC BY‐NC 4.0 [241]. Copyright 2017, Springer Nature.

The piezoresistive effect in 2D‐integrated vdW heterostructures is a fundamentally distinct phenomenon in contrast to conventional bulk materials. The mechanical strain induces dramatic changes in electrical resistance through a number of different mechanisms. The participation of interlayer coupling, band structure modulation, and interfacial charge transfer dynamics [236]. Van der Waals materials exhibit outstanding gauge factors through photoelectric‐piezoresistive coupling. For instance, Yan et al. exemplified the SnS_2_‐based sensors achieving gauge factor values up to 3933 through the modulation of carrier mobility and concentration under simultaneous strain and illumination (Figure 13a–c) [237].

In the context of 2D materials, β′‐In_2_Se_3_ exemplifies a remarkable case, where compression induces a six‐orders‐of‐magnitude resistivity drop at just 1.2 GPa, yielding a giant piezoresistive gauge factor of −5.33 GPa^−^^1^, occurring not through conventional interlayer sliding but via intralayer atomic motions of middle Se atoms that modulate both band structure and ferroelectric dipoles (Figure 13d–f) [238]. MoS_2_ exhibits negative gauge factors ranging from −225 in bilayers to −50 in few‐layer samples due to strain‐induced bandgap narrowing that enhances thermally activated carrier density (Figure 13g–i) [239]. Currently, piezoresistive phenomena in 2D heterostructures are mostly reliant on inorganic complexes, particularly chalcogenides with robust vertical chemical linkages [240]_._ In 2017, Xu et al. investigated the heterointerfacial coupling characteristics of organic vdW heterostructures, including 2D donors and acceptors. The distinctive interlayer interaction in heterostructures generates supplementary piezoresistive mechanisms: Molecular charge‐transfer in BEDT‐TTF/C_60_ heterostructures has significant piezoresistance coefficients of ‐4.4×10^−^^6^ Pa^−^^1^ via interfacial charge transfer modulation (Figure 13j–m) [241]. The notion of 2D‐integrated heterostructures offers a novel 2D platform for fundamental research on interactions influenced by external inputs for the next generation of optoelectronic devices.

### 4.4 Piezocapacitive Effect

The piezocapacitive effect refers to the reversible change in a device's electrical capacitance when mechanical pressure or strain is applied. These findings are controlled by variations in electrode separation, dielectric thickness, or permittivity under deformation [242, 243].

**FIGURE 14:**
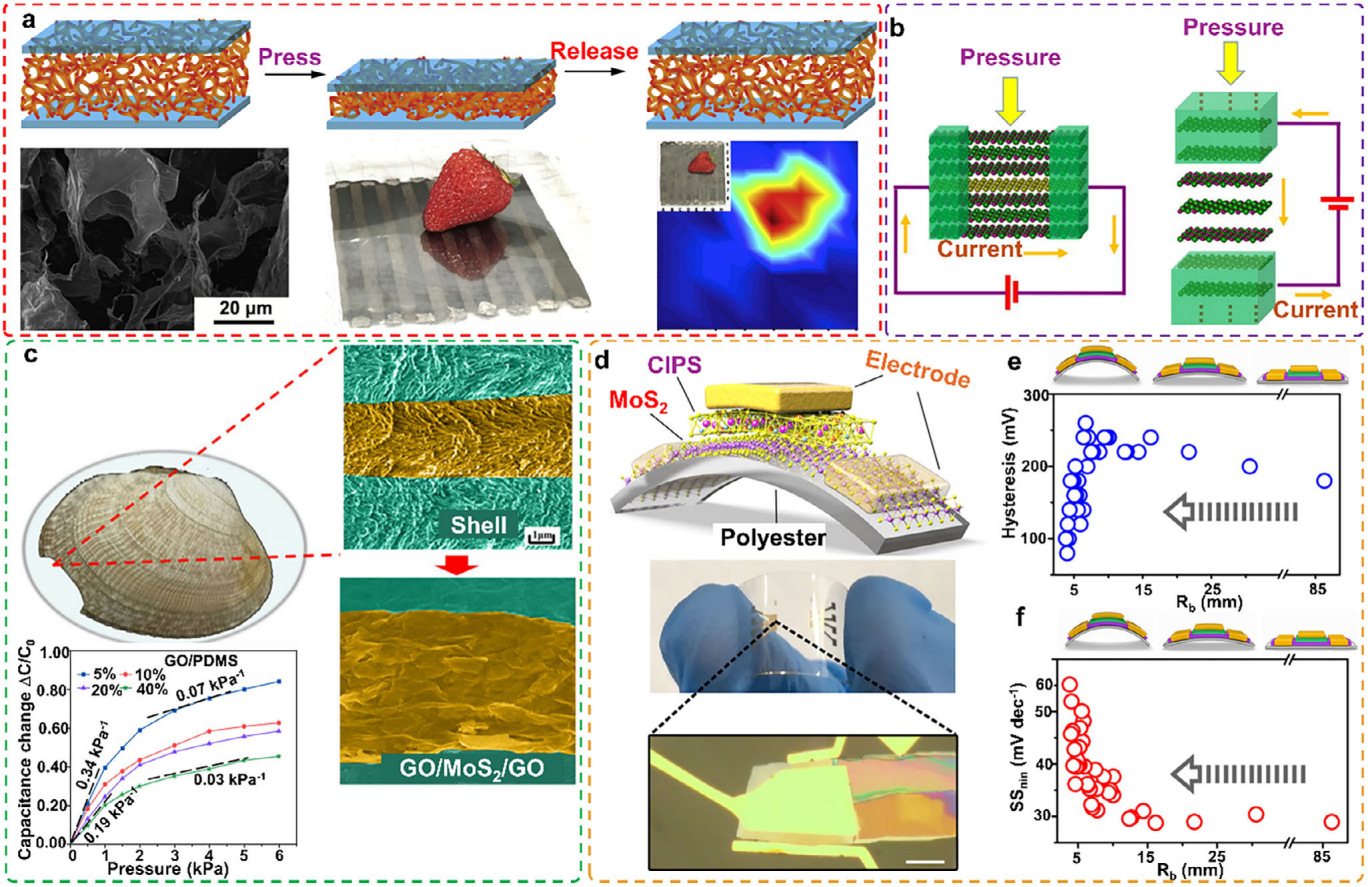
Piezocapacitive effect in 2D heterostructure. (a) Elasticity of the GO foam pressure sensor under the loading–unloading cycle. Reproduced with permission [1, 242]. Copyright 2017, Elsevier. (b) Schematic illustrating the heterostructure of in‐plane pressure sensor and tunneling pressure: h‐BN/graphene/h‐BN heterostructures and graphene/h‐BN/graphene. Reproduced with permission [248]. Copyright 2011, AIP Publishing. (c) The GO/MoS_2_/GO heterostructures pressure sensor is inspired by the multilayer structures of shellfish in nature. Reproduced with Creative Commons Attribution License (CC BY) [249]. Copyright 2024, Frontier Media S.A. (d) Van de Waals negative capacitance‐FET on flexible substrate, (e) Structural illustration and digital image of CIPS/MoS_2_ vdW NC‐FET on a flexible polyester substrate, and (f) The electrical characteristics of a flexible vdW NC‐FET with different bending states. Reproduced with Creative Commons Attribution License (CC BY) [250]. Copyright 2019, Springer Nature.

In 2D materials, these same advantages can be leveraged for use as electrodes or dielectric layers in capacitive sensors. Neglecting fringe effects, the basic model for such sensors is a parallel‐plate capacitor, where the capacitance C is directly proportional to the electrode area A, the dielectric constant, and is inversely proportional to the electrode separation distance d. When an external force is applied, the resulting mechanical deformation translates into a measurable electrical signal [242, 244]_._ Sensor sensitivity, a critical parameter for evaluating detection accuracy, is generally defined as:

(12) $$S=\frac{\Delta C}{C_{0}\times \Delta P}$$

where ΔC  =  C  −  C_0_ represents the change in capacitance. Higher sensitivity corresponds to larger output per unit pressure, which is typically achieved by maximising ΔC and minimising baseline capacitance C_0_. Importantly, the Young's modulus *E* of the material directly impacts sensitivity. According to dimensional analysis, sensor sensitivity scales with normalised pressure P/E: materials with lower *E* yield greater sensitivity under low pressure but limited detection range due to earlier deformation saturation. Capacitive variation detection is widely applied in pressure sensing and has been extensively studied in 3D structure materials and thin films with high mechanical durability, such as conductive fibres and PDMS [245, 246, 247]. In 2017, Wan et al. proposed ultra‐sensitive GO‐based capacitive pressure sensors with graphene as an electrode [244]. The GO foam exhibits both excellent elastic properties, serving as an efficient, low‐cost fabrication and large‐scale production (Figure 14a) [244].

In 2D‐integrated heterostructures, the piezocapacitive effect arises from mechanical strain‐induced changes in both geometric spacing and dielectric properties. Heterostructure‐based pressure sensors using monolayer graphene sandwiched by hexagonal boron nitride (h‐BN), or vice versa (multilayer h‐BN between multilayer graphene), demonstrate modulation of resistance through changes in interlayer spacing under external pressure [248, 251]. This effect is in‐plane current or tunneling current, ultimately modulating the electronic bandgap and thereby controlling transport channels near the Fermi level (Figure 14b) [131, 248]. In a parallel‐plate configuration, applied strain ε increases the interlayer distance d, reducing capacitance, while also modulating the relative permitivity ε_r_, via bond deformation and interfacial dipole alterations. For instance, a graphene oxide/MoS_2_/graphene oxide (G/M/G) vdW heterostructures sensor exhibited a 75–102% increase in sensitivity compared to graphene oxide alone across 0–50% strain, showing the enhanced performance enabled by vdW stacking (Figure 14c) [249]. This improvement results from the interplay of geometric deformation, tunable dielectric properties, and interfacial charge redistribution. In flexible electronic devices, the vdW ferroelectric‐MoS_2_ negative capacitance FET represents a strategy to meet the ultralow‐power operation (Figure 14d–f) [250]. The stability of the device performance under static tensile strain with various bending curvature radius, noting the prospect in the emerging wearable computing applications [250].

### 4.5 Advanced Piezo‐Coupling Effects

To enhance the performance of ultrasensitive sensors, a multi‐effect coupling approach has been developed. By using the unique properties of piezoelectric, piezoresistive, and piezocapacitive materials, the integration of strain engineering with 2D heterostructures has transformed sensing technologies. This is achieved by enabling four distinct, synergistic mechanisms: piezotronics, piezo‐phototronics, piezo‐ionic, and mechano‐photonic effects. As a result, this approach offers unprecedented sensitivity and multifunctionality for next‐generation sensing platforms.

**FIGURE 15:**
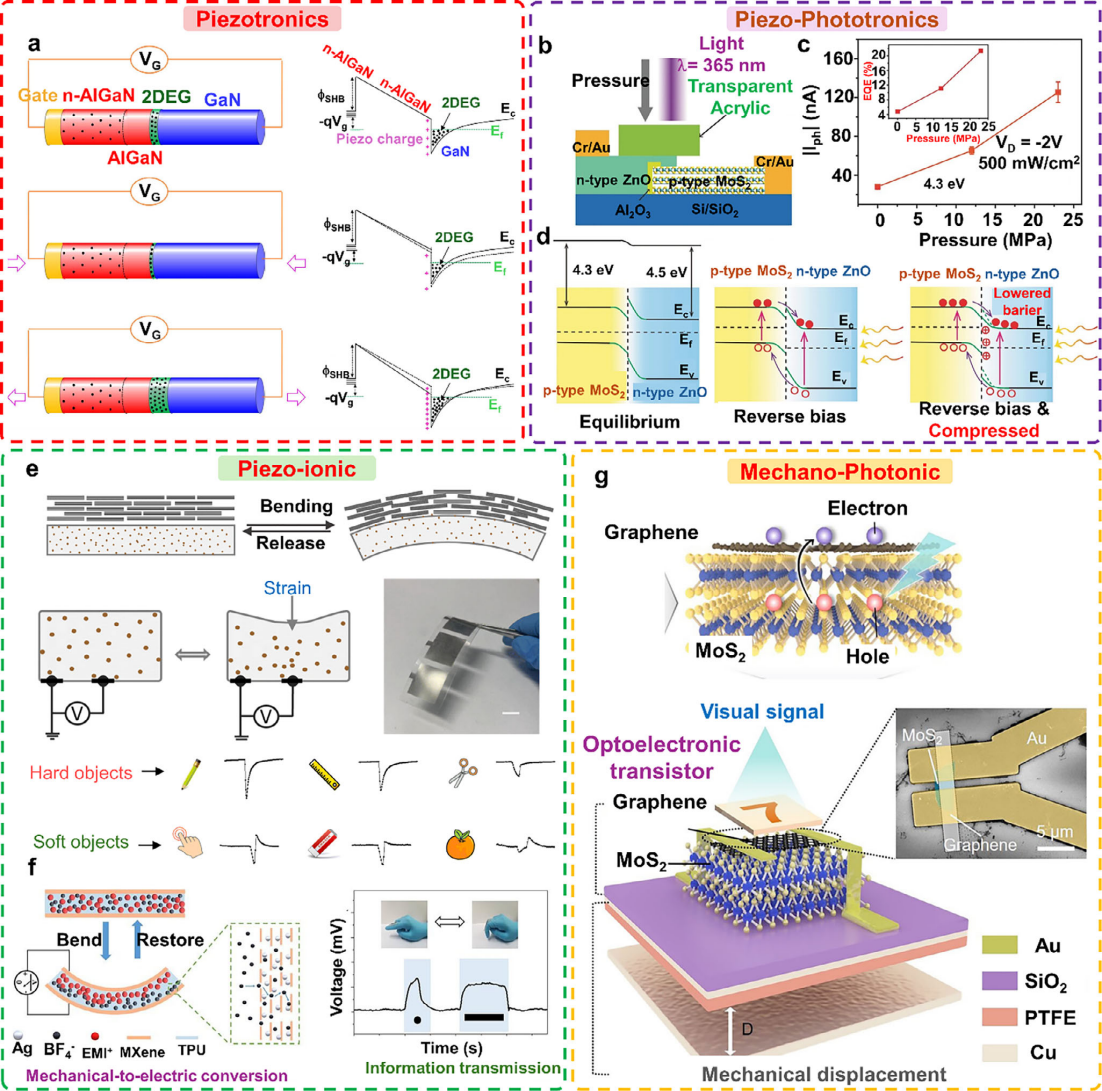
Advanced piezo‐coupling effects in 2D heterostructures. (a) The schematic diagram of AlGaN/GaN heterostructure and its potential profile of the conduction band under no strain, compressed strain, and stretched strain. Reproduced with permission [256]. Copyright 2022, Elsevier. In compressed strain, piezoelectric charges in the interface between AlGaN and GaN decrease, leading to the interfacial electrical field becoming weak, and thus the concentration of electrons in GaN reduces. Under stretched strain, piezoelectric charges increase, giving rise to an enhancement of the interfacial electrical field and high electron concentration, (b) Schematic illustrates the p‐MoS_2_/n‐ZnO heterostructure photodiode, (c) The increase of *I*_ph_ as a function of pressure applied, under drain bias of −2 V and power density of 500 mV, (d) Band diagrams demonstrating the photogenerated carriers and the photocurrent enhancement due to the piezophototronic effect. The green lines in the band structure denote the corresponding depletion region. Reproduced with permission [257]. Copyright 2016, Wiley. (e) Schematic illustrates the vdW Graphene/Nafion structure for self‐powered piezo‐ionic sensor applications. Reproduced with permission [258]. Copyright 20123, RSC. (f) The sensing mechanism of the piezo‐ionic sensors based on ion migration under bending deformation by using MXene/Ag heterostructure electrodes. Reproduced with permission [259]. Copyright 2024, Elsevier. (g) Schematic diagram of the mechano‐photonic artificial synapse based on Graphene/MoS_2_ heterostructure. Reproduced with Creative Commons CC BY license [260]. Copyright 2021, AAAS.

As mentioned in the previous Section 4.2, piezoelectricity in 2D‐inegrated heterostructures arises when mechanical strain in noncentrosymmetric monolayers (MoS_2_, WSe_2_, In_2_Se_3_). This generates a static internal piezopotential without directly steering free carriers, serving as a passive source of charge for sensors and energy harvesters [220, 226, 254]. In contrast, piezotronics harnesses this strain‐induced piezopotential as a mechanical gate to actively modulate carrier injection at Schottky or p–n junctions within 2D heterostructures. Yu et al. demonstrated a highly sensitive strain sensor based on a piezotronic tunneling junction (Ag/HfO_2_/n‐ZnO). The resulting piezoelectric polarisation creates localised electric fields that effectively lower Schottky barrier heights, dramatically enhancing conductivity with gauge factors exceeding 4.8 × 10^5^ at a strain of 0.10% [255]. Another study of Huang et al. investigated the piezotronic effect on a 2D AlGaN/GaN heterostructure (Figure 15a) [256].  The results demonstrate that strain is a more precise way to control 2D heterostructures in comparison with gate voltage. Moreover, the piezotronic effect can sufficiently raise the density of states of the quantum ground state by decreasing the energy level, and thus can be used to enhance Rashba spin‐orbit coupling in wide‐gap piezoelectric semiconductors [256].

The piezo‐phototronic effect extends this principle by coupling among piezoelectricity, photonic excitation, and semiconductor, where strain‐modulated piezopotential influences photogenerated carrier generation, separation, and transport [218]. This interesting effect could effectively increase the light emission of LEDs or the photocurrent of photodetectors. In the study of Xue et al., the enhanced photoresponse of p‐type MoS_2_/n‐type ZnO heterostructure photodiode by using the piezophototronic effect, as demonstrated in Figure 15b–d [258]. Under applied pressure, the positive piezoelectric charges yielded at the bottom surface of ZnO film will lower the barrier at the interface and make the conductive and valence bands of ZnO film go downward, which is equivalent to enlarging the depletion zone, as shown in the right‐hand diagrams. This will contribute to the generation and separation of photogenerated carriers in the p‐n heterojunction, as well as tuning the photocurrent by the piezophototronic effect [257].

Converting physical forces into electrical impulses is essential in self‐powered strain sensors. In contrast to piezoelectric materials, piezo‐ionic materials exhibit significant promise in self‐powered strain sensors due to their passive properties and directional identification capabilities resulting from ion movement under pressure. Li et al. demonstrated that the vdW graphene coating and Nafion attached by intermolecular forces maintain conformality and low interface impedance under strain (Figure 15e) [258]. In 2024, Li et al. investigated an MXene/Ag heterostructure‐based piezoionic sensor for wearable sensing applications (Figure 15f) [259]. This piezoionic sensor, with a bending deformation of 0.7% strain, exhibited a substantial voltage output of 11.1 mV and exceptional stability with signal retention of 95% over 13,000 seconds of cyclic bending. These findings introduce an innovative approach for creating self‐powered piezoionic sensors and their prospective uses in human detection, medical rehabilitation, and information transfer. In this review, another sensing method referenced is the mechano‐photonic system. While mechano‐photonic shares similarities with piezophototronics in its utilisation of mechanical stimuli and optical effects. They further encompass a wider spectrum of effects, integrating mechanical and photonic components without relying on piezoelectric effects. Consequently, Yu et al. constructed 2D graphene/MoS_2_ heterostructures by integrating triboelectric nanogenerators (TENGs) with 2D optoelectronic devices (Figure 15g) [260]. Mechanical displacement induces a triboelectric potential that concurrently regulates electronic attributes via charge transfer modulation and optical responses through persistent photoconductivity. This synergistic interaction allows heterostructures such as graphene/MoS_2_ to attain mechanical displacement‐assisted optoelectronic synaptic plasticity, enabling the device to operate as both a mechanical sensor and an optical memory component [260]. These linked mechanisms collectively facilitate the creation of multifunctional sensors adept at concurrent mechanical, electrical, and optical signal processing, thereby advancing applications in flexible electronics, biomedical implants, and interactive human‐machine interfaces [261]. To better understand the trade‐offs inherent in different sensor technologies, a comprehensive comparison of prominent sensing mechanisms, highlighting their fundamental operating principles, advantages, and limitation, is summarized in Table 6.

**TABLE 6:** Comparative analysis of prominent sensing mechanisms.

Mechanism	Physical origin	Key advantage	Primary limitation/Trade‐off	References
**Photovoltaic**	Photon absorption generates electron‐hole pairs, separated by a built‐in electric field	Self‐powered energy harvesting; highly mature technology	Performance is strictly constrained by illumination intensity and material bandgap	[262]
**Piezoelectric**	Strain‐induced generation of internal dipole moments (polarisation charges)	Self‐powered; exceptional response to high‐frequency dynamic stimuli.	Incapable of measuring static (constant) strain; high internal impedance	[263]
**Piezoresistive**	Strain‐induced modulation of bulk band structure and carrier mobility	High linearity and simple readout circuit	Moderate sensitivity; continuous static power consumption	[264]
**Piezocapacitive**	Strain‐induced variation in electrode spacing or dielectric permittivity	Ultra‐low static power consumption	Highly susceptible to unintended capacitance and external noise	[264]
**Piezotronic**	Piezo‐polarisation charges modulate the interface energy barrier (e.g., Schottky junction, p‐n junction)	Ultra‐high sensitivity due to exponential current modulation	Highly susceptible to interface trap states and temperature fluctuations	[265]
**Piezo‐phototronic**	Interface barrier modulation tunes the separation/transport of photogenerated carriers	Drastically enhances optoelectronic responsivity and efficiency	Requires pristine junction quality; increases system complexity	[265]

## 5 Sensing Applications

The critical effect of strain in realising this ultra‐sensitivity lies in its ability to modulate the electrical properties of 2D vdW heterostructures [171]. For instance, even subtle mechanical deformations can induce giant modulations in the electronic band structure and interlayer coupling, leading to highly efficient strain‐to‐electrical signal transduction [238]. Currently, sensors based on 2D heterostructures are highly promising for advanced ultrasensitive applications. While traditional metal foil and bulk silicon strain gauges typically exhibit GF of approximately 2 and 100 [264], respectively, 2D vdW heterostructures can be classified as ultrasensitive when their GFs exceed 10^3^ to 10^6^ [267]. Consequently, this extreme sensitivity allows the sensors to detect ultra‐low pressures (< 500 Pa) [236] while maintaining ultrafast response times [268] and an ultra‐low photodetection limit [269]. Building upon the significant merits of individual 2D materials, the formation of 2D‐integrated heterostructures provides a novel approach to further enhance their outstanding electronic, photonic, mechanical, and thermal properties. When combined with flexible substrates, these heterostructures have been widely utilised in ultrasensitive photodetectors, image sensors, artificial synapses, tactile sensors, pressure sensors, and structural health monitoring systems. This section focuses on the critical role of strain‐engineered 2D‐integrated heterostructures in enabling these advanced sensing applications, highlighting how strain modulation unlocks unprecedented sensitivity and functionality.

### 5.1 Photodetectors

**FIGURE 16:**
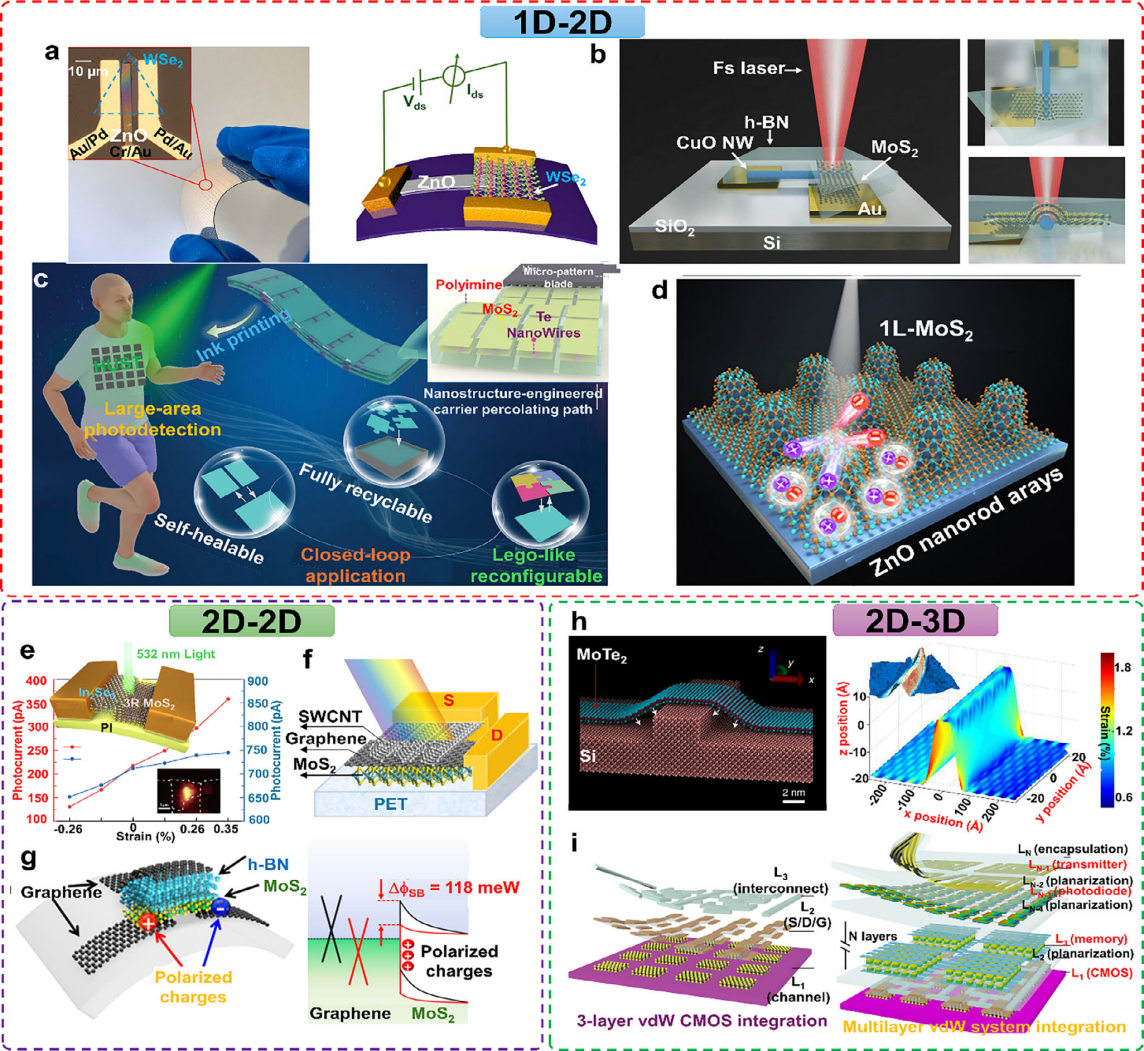
Strain‐engineered Photodetectors in 2D integration heterostructures materials. (a) Optical image of the flexible p‐n WSe_2_‐ZnO photodetector. Reproduced with permission [227]. Copyright 2019, Elsevier. (b) Schematic diagram of h‐BN encapsulated CuO/MoS_2_ 1D‐2D van de Waals heterostructure photodetector. Reproduced with permission [270]. Copyright 2023, RSC. (c) Schematic diagram of the self‐healable, recyclable, Lego‐like reconfigurable, and screen‐printable 1D (Te NWs)/2D (MoS_2_ nanosheets)/polyimine photodetector. Reproduced with permission [271]. Copyright 2024, Wiley. (d) Periodically strain‐engineered vdW of 2D (1L‐MoS_2_)/1D (ZnO) heterostructure arrays for photodetectors. Reproduced with permission [22]. Copyright 2019, ACS. (e) Schematic of strain‐modulated photoelectric responses of flexible α‐In_2_Se_3_/3R MoS_2_ heterostructures photodetector. Reproduced with Creative Commons CC BY license [272]. Copyright 2021, Springer Nature. (f) Schematic illustration of the flexible photodetector based on the double heterojunction of SWCNT/graphene/MoS_2_. Reproduced with Creative Commons CC BY license [273]. Copyright 2023, Springer Nature. (g) Schematic diagram of graphene/MoS_2_ heterostructure devices and energy band diagram of heterostructure with and without mechanical strain. Reproduced with permission [267]. Copyright 2019, ACS. (h) Strain‐engineered MoTe_2_‐Si photodetector with strain map variation. Reproduced with permission [274]. Copyright 2019, Springer Nature. i) Schematic of the building blocks for vdW assembly of 3D integration devices. Reproduced with permission [15]. Copyright 2019, Nature.

Strain and interface engineering in mixed‐dimensional 1D–2D van der Waals heterostructures has emerged as a versatile strategy to tailor interfacial band alignment, accelerate charge separation [99]. These merits dramatically boost photodetector metrics, responsivity, detectivity, and speed, while also imparting new functionalities such as self‐healing and reconfigurability. Strain‐gated WSe_2_‐ZnO photodiodes exploit the piezotronic effect of ZnO to dynamically “tune” the interfacial energy landscape under mechanical deformation (Figure 16a) [227]. Tensile strain on the ZnO nanobelt generates positive piezopolarization charges at its (001) surface, which attract electrons into the adjacent p‐type WSe_2_ and steepen its band slope at the heterojunction. This strain‐induced electrostatic gating enhances self‐powered responsivity from ∼60 pA to ∼320 pA (0–0.4% strain) and R up to 394 mA W^−^^1^ under 1 V bias, without compromising response speed. Compressive strain reverses the effect, reducing responsivity and demonstrating reversible mechanical control over carrier dynamics at the 1D‐2D interface [227]. In static strain fields, femtosecond laser contact engineering offers a post‐fabrication route to optimise 1D‐2D interfaces. The h‐BN‐encapsulated MoS_2_/CuO nanowire heterojunctions, ultrafast laser pulses wrap the MoS_2_ sheet conformally around the nanowire and ablate interfacial contaminants without degrading the h‐BN cap (Figure 16b) [270]. This dual effect increases contact area and lowers interfacial trap density, boosting the rectification ratio, reducing ideality factor (n ≈ 1.4), and yielding photodiode responsivities as high as 2.5 × 10^3^ A W^−^^1^ under forward bias or 2500 A W^−^^1^ and detectivities of 6.5 × 10^11^ Jones under reverse bias, with millisecond‐scale rise and fall times. The h‐BN encapsulation also preserves device performance under humidity and aging, solving environmental stability challenges [270].

Also building on this concept, Te nanowire/monolayer MoS_2_/polyimine heterostructures employ flow‐aligned 1D‐2D networks as “fast lanes” for carrier percolation within a dynamic covalent matrix (Figure 16c) [271]. The Te nanowires prevent MoS_2_ stacking, bridging nanosheets to form long‐range conductive paths. These ordered bridges yield carrier mobilities up to 6 times higher than random composites. This enables photodetector responsivity of 11.7 mA W^−^^1^ and detectivity of 1.1 × 10^10^ Jones, with sub‐second response times that persist after 50 000 bending cycles. Critically, the polyimine matrix endows the device with room‐temperature self‐healing (restoring >94% of mechanical strength and photocurrent after multiple cuts) and full closed‐loop recyclability and reconfiguration, which attributes are unattainable in rigid heterostructures [271]. In monolayer MoS_2_/ZnO nanorod arrays, periodic ZnO nanorods act as nanoscale indenters during transfer, imposing a spatially modulated biaxial tensile strain (up to ∼0.6%) on the MoS_2_ overlayer (Figure 16d) [22]. Confocal Raman mapping reveals localised E_2g_ shifts that pinpoint strained regions, where built‐in tensile strain reduces the energy barrier for electron transfer into ZnO. Band alignment analysis via UPS and Kelvin probe measurements confirms that tensile strain upshifts the MoS_2_ Fermi level by ∼80 meV, thereby lowering the interfacial conduction‐band barrier and accelerating photocarrier injection into ZnO [22].

Strain engineering in mixed‐dimensional 2D‐2D heterostructure photodetectors exploits the generation of piezoelectric or polarisation charges at atomically sharp interfaces to modulate local energy band alignments and dramatically amplify photoresponse [275]. For instance, In_2_Se_3_3R‐MoS_2_ p–n junctions on flexible substrates exhibit self‐powered responsivities up to 2.9 × 10^3^ A W^−^^1^ and detectivities of 6.2 × 10^10^ Jones under ‐0.26% compression by using strain‐driven piezocharges in In_2_Se_3_ to deepen the built‐in field and improve carrier separation (Figure 16e) [272]. Moreover, SWCNT/Gr/MoS_2_ detectors with Gd_3_Fe_5_O_12_ interlayers combine the graphene's mobility, MoS_2_’s visible absorption, and SWCNT's NIR absorption to reach room‐temperature responsivities of 109 A W^−^^1^ and detectivities of 4.5 × 10^12^ Jones at 1080 nm, while thin barriers suppress dark currents and preserve mechanical flexibility (Figure 16f) [273]. Likewise, graphene/MoS_2_ FETs achieve ultrahigh gauge factors (∼5.75 × 10^5^) by exploiting strain‐induced ion‐charge polarisation in MoS_2_ that shifts graphene's Fermi level near its Dirac point, modulating the Schottky barrier by ∼118 meV and yielding up to 978‐fold current changes at only 0.17% strain (Figure 16g) [267].

In mixed 2D‐3D integration devices, by wrapping multilayer MoTe_2_ (2D) around non‐planarized silicon waveguides or microring resonators (3D), localised tensile strains of ∼3% reduce the MoTe_2_ bandgap from ∼1.04 to ∼0.80 eV, enabling efficient absorption and photocarrier generation at 1550 nm (Figure 16h) [274]. This bond‐free integration yields low dark currents (∼13 nA), high responsivities up to 0.5 A W^−^^1^, and noise‐equivalent powers as low as 90 pW Hz^−0.5^, while retaining GHz‐scale speed through carrier‐transit‐limited operation [274]. The mixed‐dimensional heterostructures combine the mechanical flexibility and defect‐free interfaces of 2D materials with the mature fabrication and optical confinement of 3D photonic circuits, opening a pathway to compact, high‐performance on‐chip photodetectors for integrated optoelectronics (Figure 16i) [15]. Collectively, these studies establish that nanoscale strain, whether patterned by substrate features, embedded in dynamic matrices, delivered by ultrafast lasers, or imposed through bending. This will serve as a powerful “gate” to modulate band offsets, accelerate interfacial charge transfer, and impart multifunctionality in mixed‐dimensional photodetectors. This paradigm paves the way for next‐generation flexible, high‐density, and adaptive optoelectronic systems that seamlessly integrate performance, durability, and reconfigurability.

### 5.2 Image Sensors

**FIGURE 17:**
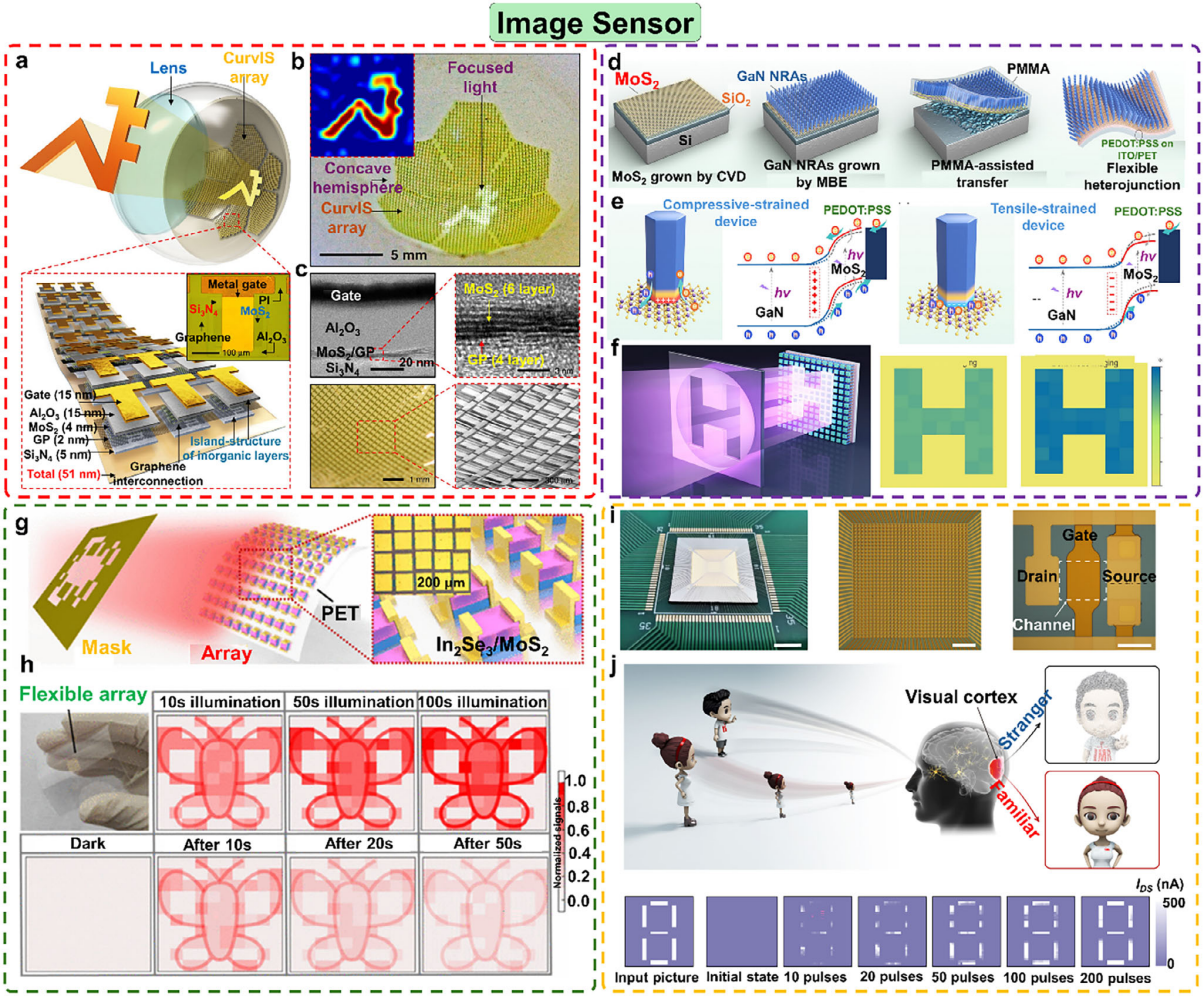
Flexible Image Sensor Applications. (a) Human eye‐inspired soft optoelectronic device using MoS_2_‐graphene heterostructure curved image sensor array, with the inset showing the image of a single phototransistor, (b) Optical camera image of the array, (c) TEM and SEM image of the array. Reproduced with Creative Commons CC BY license [276]. Copyright 2017, Springer Nature. (d) Schematic illustrates the fabrication process of vertical 1D GaN Nanorods/2D MoS_2_/PEDOT:PSS self‐powered flexible image sensors, (e) Energy band diagrams of GaN/MoS_2_/PEDOT:PSS heterojunction under UV illumination at compressive and tensile strain, (f) The schematic of the image sensing analysis with clear imaging output “H” under flat‐mode and bent‐mode. Reproduced with Creative Commons CC BY license [277]. Copyright 2025, Springer Nature. (g) Flexible In_2_Se_3_/MoS_2_ heterostructure devices for an artificial vision system in the near‐infrared range with imaging functions of the In_2_Se_3_/MoS_2_ device array, with the inset showing the single device, (h) Illustrations of the imaging function of the “butterfly” at different times of illumination and dark. Reproduced with permission [278]. Copyright 2022, ACS. (i) Flexible ultrasensitive optoelectronic CNT/CsPbBr_3_‐QD heterostructure sensor array for neuromorphic vision systems, and (j) Schematic of the impression on human visual systems when strange and familiar faces are observed, and 8 patterns are measured by training with 10, 20, 50, 100, 200 pulses under light illumination. Reproduced with Creative Commons CC BY license [279]. Copyright 2021, Springer Nature.

Strain engineering has emerged as a powerful tool to optimise the performance of flexible image sensors based on 2D‐integrated heterostructures, with applications ranging from wearable artificial vision to implantable retinal prostheses [260]. By integrating atomically thin semiconductors, such as MoS_2_, In_2_Se_3_, graphene, GaN nanorods, and colloidal perovskite quantum dots, researchers have created photodetector arrays whose photoresponse, responsivity, and detectivity can be dynamically tuned by applying small uniaxial or biaxial strains [205, 271, 274]. For example, ultrathin MoS_2_/graphene phototransistor arrays conformally laminated into hemispherical “curved image sensor” domes achieve IR‐blind, high‐density imaging without brittle fractures, leveraging 23% fracture‐strain of MoS_2_ and the PI/Si_3_N_4_ strain‐isolation design to maintain mechanical integrity (Figure 17a–c) [276]. Another study research on strain‐tunable GaN nanorod/MoS_2_/PEDOT:PSS photodiodes attains a self‐powered responsivity enhancement from 1.47 to 2.47 A W^−^^1^ under ‐0.78% compressive strain, with sub‐50 µs response times, facilitating flexible UV detection and signature imaging (Figure 17d–f) [277]. In the study of Hu et al., flexible In_2_Se_3_/MoS_2_ heterojunction synaptic devices on PET substrates exhibit up to a 27.6% increase in synaptic weight change (ΔEPSC) under 0.54% tensile strain, improving near‐infrared (1060 nm) image sensing, learning, and storage functions in a 10 × 10 device array (Figure 17g,h) [278]. Additionally, carbon nanotube–perovskite quantum dot heterostructure sensor arrays embedded in stretchable substrates display ultrahigh detectivity (2 × 10^16^ Jones) and plasticity, enabling reinforcement‐learning vision systems that robustly distinguish weak light pulses (Figure 17i,j) [279]. On these platforms, strain not only modulates band alignments and improves built‐in fields with carrier separation, but it also fine‐tunes photogating effects, making them more responsive, faster, and better at storing memory. By harnessing strain‐engineering in 2D heterostructures, next‐generation flexible image sensors promise ultralow‐power, mechanically robust interfaces for wearable health monitors, soft robotics vision, and bio‐integrated artificial vision systems.

### 5.3 Artificial Synapses

In 2D heterostructures, strain engineering has emerged as a powerful strategy for improving artificial synaptic functions, providing unparalleled control over neuromorphic device functionality. Utilising the distinct mechanical flexibility and strain‐sensitive electronic properties of 2D materials, strain effects in 2D synaptic devices significantly contribute to attain advanced neuromorphic functions.

**FIGURE 18:**
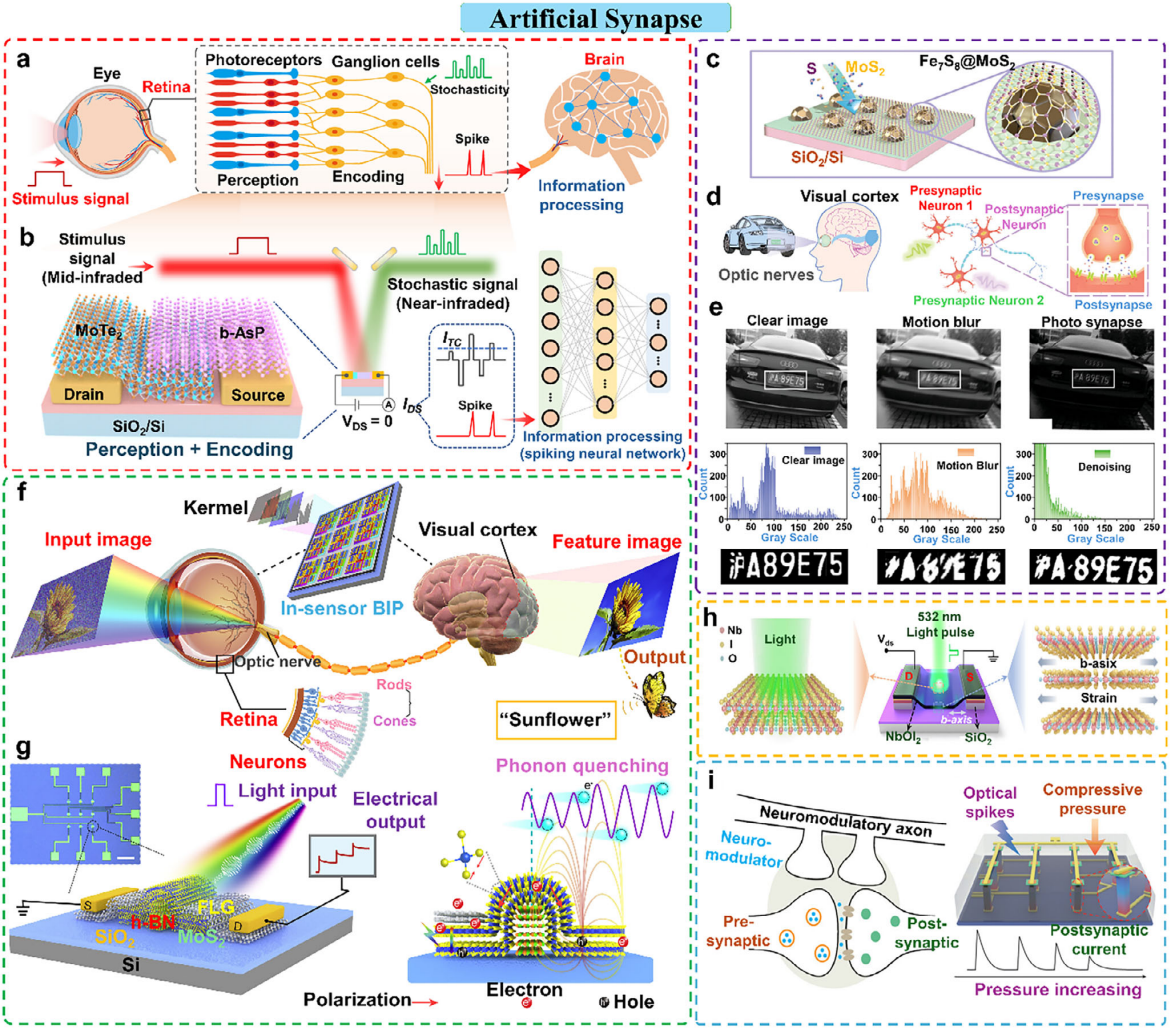
Artificial Synaptic in 2D heterostructure materials. (a) The visualisation of perception, encoding, and processing of stimulus signals from external objects in the human visual system. (b) The proposed 2D b‐AsP/MoTe_2_ vdWs heterostructure device mimics the retina functionalities. Reproduced with Creative Commons CC BY license [280]. Copyright 2023, Springer Nature. (c) Schematic illustration for the synthesis of Fe_7_S_8_@MoS_2_ heterostructures with the strain engineered from the curved geometry, (d) The illustration of the human visual system and biological synapses, (e) Clear vehicle image, under motion, and after preprocessing. Reproduced with permission [62]. Copyright 2024, Wiley. (f) Retina‐inspired in‐sensor of MoS_2_ for recognition of images, (g) The visualisation process of recognising a sunflower after the flower image is sensed by photoreceptors with inhomogeneous strain is applied. Reproduced with permission [281]. Copyright 2023, Elsevier. (h) The 2D NbOI_2_ Piezo‐Ferro‐Opto‐Electronic Artificial Synapse for multifunctional neuromorphic devices. Reproduced with permission [282]. Copyright 2025, Wiley. (i) Strain‐modulated ZnO/Al_2_O_3_/CdS heterostructures optoelectronic synapse array. Reproduced with Creative Commons CC BY license [283]. Copyright 2023, Wiley.

In a complementary vein, mid‐infrared (MIR) Black Phosphorus‐Arsenic (b‐AsP)/MoTe_2_ heterostructures exploit sub‐micrometre strain and built‐in fields to achieve zero‐bias photothermoelectric detection and stochastic near‐infrared sampling, converting MIR intensity directly into stochastic spike trains suitable for spiking neural network classification with >96% accuracy (Figure 18a,b) [280]. Similarly, the Fe_7_S_8_@MoS_2_ dome‐like heterostructure exploits strain‐induced changes in the MoS_2_ shell to create effective charge trapping centres, enabling remarkable retention times of up to 800 seconds under single optical pulses (Figure 18c–e) [62]. In another study, by patterning a three‐dimensional strain‐stabilised (3DSS) architecture into MoS_2_/graphene opto‐synapses, local strain gradients induce permanent flexoelectric polarisation that both boosts broadband photodetection from ultraviolet through visible. This method produces tunable long‐term plasticity under pulsed illumination, which leads to energy‐efficient synaptic potentation and paired‐pulse facilitation at the femtojoule scale (Figure 18f,g) [281]. In the latest study of Wang et al., the synergistic integration of ferroelectricity, piezoelectricity, and optoelectronic properties is demonstrated by the NbOI_2_‐based 2D PFOE (Piezo‐Ferro‐Opto‐Electronic) artificial synapse. The result exemplifies how tensile strain enhances spontaneous polarisation and significantly improves paired‐pulse facilitation from 116 to 180% (Figure 18h) [282]. In a different study, the ZnO/Al_2_O_3_/CdS heterostructure demonstrates how synaptic plasticity can be greatly increased by piezo‐phototronic effects under mechanical strain. More precisely, the heterostructures can boost memory retention capacity by up to 30% and decrease the training periods for facial recognition by 36% (Figure 18i) [283].

Together, these works demonstrate that deliberate strain‐engineering in 2D heterostructures can not only enhance photosensitivity and carrier mobility via flexo‐ or piezotronic effects but also provide versatile, wavelength‐agnostic platforms for in‐sensor preprocessing, encoding, and synaptic emulation in artificial vision and computing architectures [285, 286].

### 5.4 Temperature‐Strain Sensor

**FIGURE 19:**
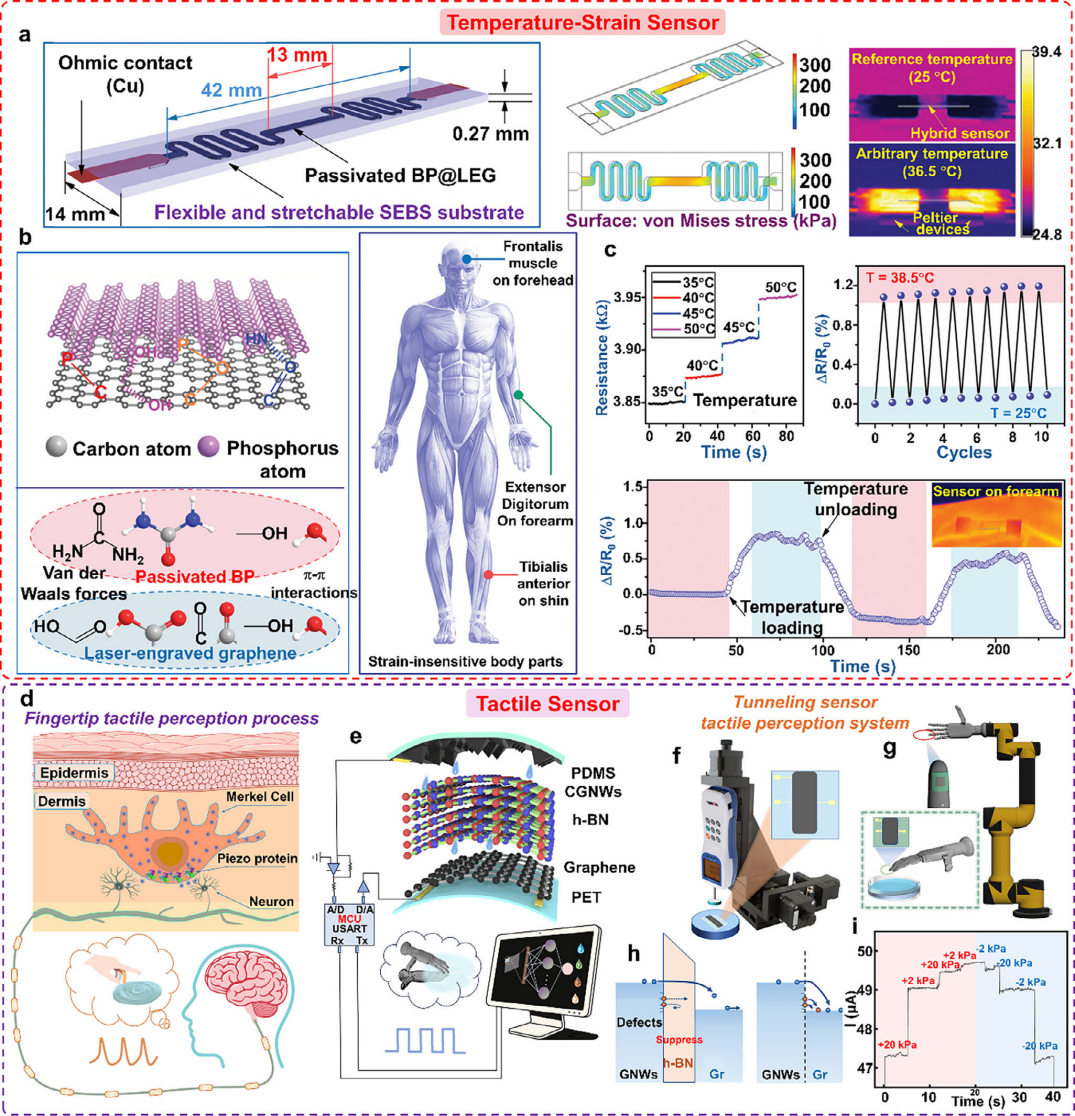
Temperature‐Strain Sensor. (a) Schematics illustration and FEA simulation of Black Phosphorus@Laser‐Engraved Graphene Heterostructure Sensor, (b) Illustration of 3D chemical cross‐linking of BP@LEG hybrid with BP and LEG are closely combined through the lattice dynamics of Van de Waals forces (P−O−C, C═O, C═(O)N−H) and stable *π*–*π* chemical interaction, (c) The application of strain‐insensitive body parts for real‐time temperature measurement. Response of the hybrid sensor in the physiological temperature range. Infrared thermal images during temperature characterisation. Reproduced with permission [148]. Copyright 2021, Wiley. Tactile sensor. (d) 2D vertical tunneling heterostructure for human fingertip tactile sensing system, (e) Structure and sensing process of tactile sensor, (f) Schematic of the sensor test environment, (g) Schematic of the sensor for liquids testing, (h) Comparison of energy band diagrams between the Graphene Nanowalls (GNWs)/h‐BN and GNWs/Gr, and (i) IT response to sequential loading and unloading pressures of 20 kPa, 2 kPa, 20 Pa, and 2 Pa. Reproduced with permission [287]. Copyright 2025, ACS.

The development of multifunctional sensors based on 2D heterostructures has greatly improved electronic skin (e‐skin) and temperature sensing applications. Figure 19a–c illustrate the black phosphorus@laser‐engraved graphene (BP@LEG) heterostructure devices with exceptional dual‐modal sensing capabilities of temperature‐strain sensors [148]. The results demonstrate a high thermal coefficient of resistance (TCR) of 0.1736% °C^−^^1^ and remarkable strain sensitivity, with a gauge factor of 2765 at a strain level exceeding 19.2%. In the heterostructures, while laser‐engraved graphene offers better mechanical flexibility and strain‐responsive crack propagation mechanisms, BP has tunable electronic properties and improves electron‐phonon interactions for temperature sensing [148].

### 5.5 Tactile Sensor

In a similar tendency, tactile sensors based on conformal graphene nanowalls‐hexagonal boron nitride‐graphene (CGNWs‐hBN‐Gr) vdW heterostructures demonstrate exceptional performance thanks to a bioinspired design that mimics Merkel cell mechanoreceptors (Figure 19d–i) [287]. The hBN tunneling layer acts as a noise suppressor, lowering the noise power spectral density to 2.22 × 10^−24^ A^2^/Hz while enabling ultrahigh sensitivity of 1.99 × 10^6^ kPa^−^^1^ and a signal‐to‐noise ratio of 68.76 dB. The 2D heterostructure‐based sensors incorporate quantum tunneling effects, vdW interlayer coupling, and strain‐engineered band structure modulation to achieve exceptional sensing capabilities. These results surpass traditional sensors, making them ideal for applications requiring simultaneous detection with minimal crosstalk and outstanding resolution down to human tactile perception thresholds.

### 5.6 Pressure Sensor

**FIGURE 20:**
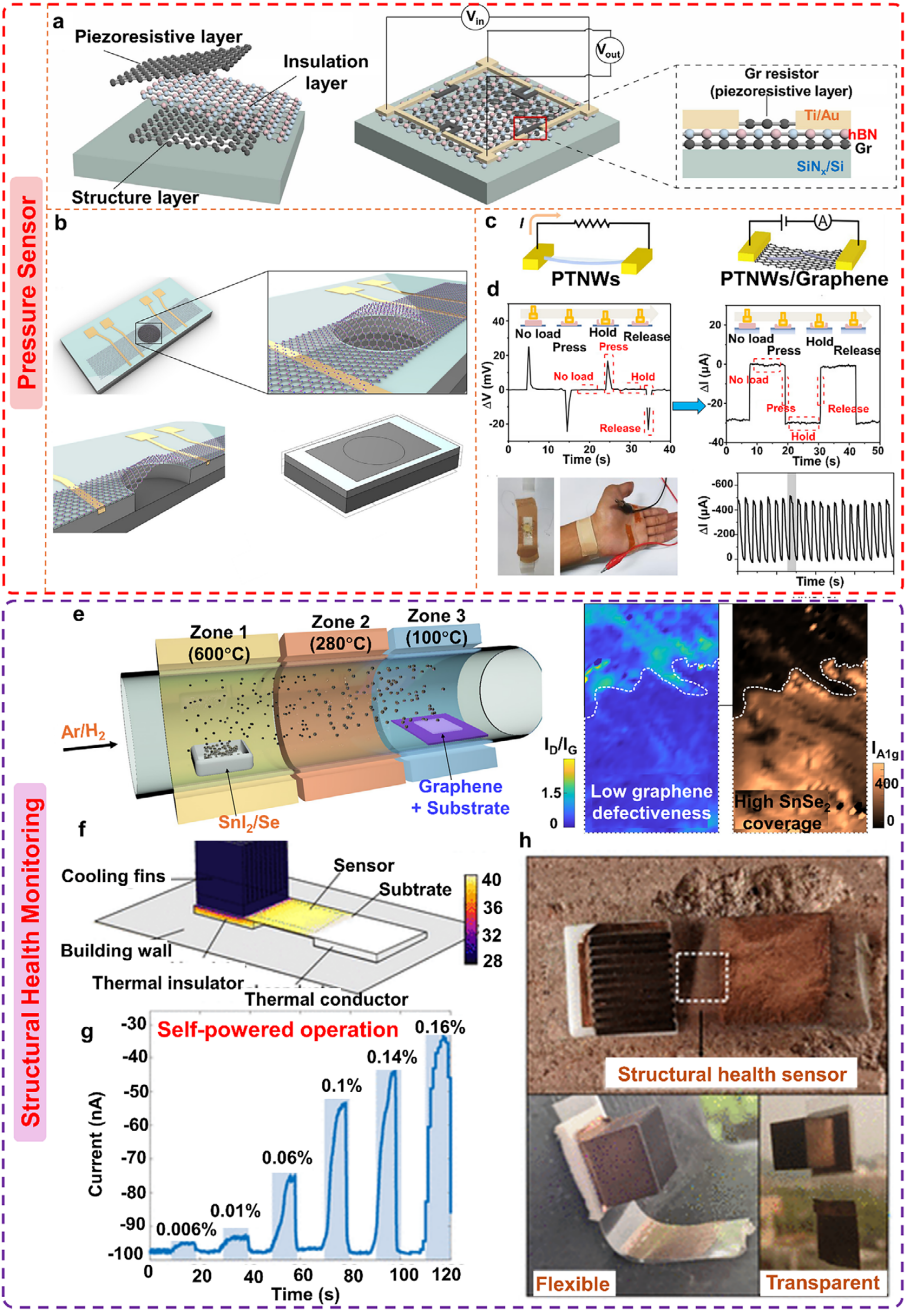
Pressure sensor. (a) Schematic of the suspended Graphene/h‐BN/Graphene‐resistor 2D heterostructure‐based sensors. The 2D heterostructure device has a bottom graphene layer as the structure layer, h‐BN as the insulation layer, and a top graphene layer as the piezoresistive layer. Reproduced with permission [236]. Copyright 2024, Elsevier. (b) Schematic of NEMs pressure sensors based on graphene‐based heterostructures (Gr/h‐BN, Gr/MoS_2_, Gr/MoSe_2_). Reproduced with permission [290]. Copyright 2024, ACS. (c) Flexible piezoelectric‐induced pressure sensors on PbTiO_3_ NWs/Gr heterostructures, (d) Pressure responses of a pure PNTW and a Gr‐based pressure sensor with a photograph of the measurement for wrist pulses. Reproduced with permission [291]. Copyright 2017, ACS. Structural health monitoring sensor. (e) A novel self‐powered strain sensor SnSe_2_/Gr heterostructure for structural health monitoring with fabrication process of Gr‐assisted growth of SnSe_2_ in three‐zone CVD techniques, (f) Finite element simulation of heat distribution under representative ambient conditions, (g) The photocurrent of the self‐powered device operated under different applied strain values, and (h) Photographs of the device working in different SHM environments. Reproduced with permission [293]. Copyright 2021, ACS.

2D‐integrated heterostructures have become a groundbreaking configuration for applications requiring ultrasensitive pressure sensing. These advantages provide unparalleled performance through strain‐engineered mechanisms that exceed traditional silicon‐based sensors. The recent combination of graphene with hexagonal boron nitride (h‐BN) has shown exceptional promise. Sandwich‐like Graphene/h‐BN/Graphene heterostructures have sensitivities that are up to five times higher than those of regular sensors (Figure 20a) [236]. These heterostructures exploit several sensing mechanisms simultaneously, including piezoresistive effects that alter the carrier mobility and scattering mechanisms that modulate electrical transport characteristics. Recent findings indicate that tunneling‐based pressure sensors utilising G/h‐BN/G heterostructures can achieve remarkable sensitivities of 8.31 × 10^−^^3^ kPa^−^^1^. In which the applied pressure modifies the tunneling barrier height and enhances carrier transport [132, 288, 289]. When compared with individual 2D materials, these heterostructures have much better mechanical properties. For example, MoS_2_/graphene heterostructures have shown Young's modulus improvements by factors of up to five due to interlayer interactions (Figure 20b) [290]. Additionally, piezoelectric‐induced pressure sensors based on nanowire/graphene heterostructures have demonstrated the capability for static pressure measurements with sensitivities up to 9.4×10^−^^3^ kPa^−^^1^ and response times as fast as 5–7 ms. This finding was achieved through synergistic mechanisms between strain‐induced polarisation charges and graphene carrier scattering (Figure 20c,d) [291]. Under high‐pressure conditions, these materials exhibit unique quantum sensing capabilities, with boron vacancy defects in h‐BN demonstrating pressure sensitivities of 0.32 MPa/Hz and enabling in situ pressure mapping with spatial resolutions at the nanoscale [292].

### 5.7 Structural Health Monitoring Sensor

Structural health monitoring (SHM) represents a revolutionary application of 2D‐integrated heterostructures that combine advanced materials science with next‐generation sensing technology. In Figure 20e–h, the SnSe_2_/graphene heterostructure exemplifies how atomic‐scale engineering can create multifunctional sensors capable of both self‐powered operation and unprecedented strain sensitivity [293]. This heterostructure exploits the strong interfacial interactions between tin diselenide and graphene to produce thermoelectric and mechanoelectric properties that exceed the capabilities of either individual component. The system operates through a novel 2D‐crack‐assisted strain‐sensing mechanism, where spatially varying interactions at the SnSe_2_/graphene interface create strain localisation that results in controlled crack formation under minimal strain. This crack‐based sensing achieves an extraordinary gauge factor of 450, significantly outperforming other 2D materials and enabling the detection of structural deformations as small as 0.02% strain.

Beyond conventional physiological and structural monitoring, the rapid evolution of strain‐engineered 2D materials has catalysed the development of highly sensitive and durable strain sensors. This can maintain high performance across diverse and demanding applications [294, 295]. Furthermore, the exploration of these sensors in extreme environments deserves explicit attention, as it highlights their versatile sensing capabilities. Unlike traditional silicon‐based or metallic sensors, which often experience severe performance degradation or mechanical failure under severe conditions, advanced 2D material‐based strain sensors leverage strong in‐plane covalent bonding and flexible interlayer vdW interactions to withstand immense physical and thermal stresses [296, 297]. As a result, these strain‐engineered devices are demonstrating reliable operability in practical harsh scenarios, including extreme temperature variations, high‐humidity or underwater operations, severe mechanical fatigue, and chemically corrosive environments [298, 299]. This remarkable environmental resilience not only underscores the fundamental structural superiority of 2D materials but also expands the practical applications of next‐generation strain sensors in demanding industrial and healthcare sectors. To benchmark the current progress in the field, Tables 7 and 8 systematically compare the sensing performance and mechanical stability of prominent 2D material‐based flexible photodetectors and strain sensors, respectively.

**TABLE 7:** Performance comparison of flexible photodetectors based on 2D‐integrated heterostructures.

2D Heterostructure material	Bias (V)	Wavelength (nm)	Responsivity (A/W)	Detectivity (Jones)	Strain‐induced enhancement	References
Graphene/ZnO	−1	325	84.94	—	Photocurrent increases 17% under −0.349% strain	[300]
MoS_2_/CuO	2	Vis – NIR	42 × 10^−3^	—	Responsivity increases 69.7% under 2% strain	[301]
WSe_2_/ZnO	1	532	0.394	∼2.0 × 10^10^	Photocurrent increases 6.3 times under 0.87% tensile strain	[227]
MoTe_2_/Graphene	1	1064	60	1.5 × 10^11^	Maintain excellent responsivity after 5000 bending cycles	[302]
CsPbBr_3_/Ti_3_C_2_Tx	10	450	0.045	6.4 × 10^8^	Maintain excellent responsivity and fast response time after thousands of bending cycles	[303]
PbI_2_/Gr	2	480	45	—	Maintain excellent responsivity after 100 bending cycles	[304]
α‐In_2_Se_3_/β‐InSe	—	365	19.9×10^−3^	—	Responsivity increases 7.62 times (at 635 nm) and 12.3 times (at 780 nm)	[201]
h‐BN/CuO/MoS_2_	5	525	2500	6.5 × 10^11^	Responsivity increases by 2 orders of magnitude	[270]
MoTe_2_/Si	−2	1550	0.5	—	Reduces bandgap from 1.04 eV to 0.8 eV under 35 tensile strain	[274]
1D GaN Nanorods/2D MoS_2_/PEDOT:PSS	—	532	2.47	2.6 × 10^11^	Responsivity increase 68.2% under ‐0.78% compressive strain and detectivity increases 2 times	[277]
α‐In_2_Se_3_/3R‐MoS_2_	—	Vis – NIR	2.9 × 10^3^	6.2 × 10^10^	Responsivity increases 88% and detectivity rises 46% at ‐0.26% compressive strain	[272]
SWCNT/Graphene/MoS_2_	—	450 – 1080	47.375	4.504 × 10^12^	Responsivity increases nearly 30 times and three orders of magnitude higher in detectivity	[273]
Te NWs/MoS_2_	—	532	11.68 × 10^−3^	1.145 × 10^10^	Responsivity increases 20.1 times and 6.9 times in detectivity	[271]

**TABLE 8:** Performance comparison of strain sensors based on 2D materials and 2D‐integrated Heterostructures.

2D material/2D heterostructure material	Bias (V)	Gauge factor (GF)	Strain‐induced enhancement	References
MoS_2_ (FET)^a^	20	40	Gate‐tunable sensitivity increases by >1 order of magnitude	[179]
MoS_2_/porous graphene	—	24.1	The relative resistance increases 80 times under strain	[305]
MoS_2_/Graphene (FET)*	−0.15	575294	Current surges 978 times under 0.17% tensile strain. Ultrahigh Gauge Factor	[267]
SnSe_2_/Graphene	—	450	Gauge factor increases 400 times under 0.4 – 0.5% tensile strain	[293]
Black Phosphorus/Graphene	—	2765	Gauge factor increases ∼34 times with high thermal index of 8106 K and ultralow strain resolution of 0.023%	[148]

^a^ FET: Field‐effect transistor.

## 6 Conclusion and Future Perspectives

This review provides a comprehensive overview of the recent advances in strain engineering of 2D‐integrated heterostructures aimed at ultrasensitive sensing applications. Beginning with a general definition of 2D heterostructures, we introduce the concept of 2D integration as a versatile approach that leverages dimensional diversity to enhance material performance. The underlying physical mechanisms governing the formation of integrated heterostructures are examined, alongside their distinctive functional properties. Two principal categories of techniques for fabricating and applying strain in 2D‐integrated heterostructures are systematically summarised. Furthermore, the detailed sensing mechanisms, which play a key and foundational role in the application of 2D‐integrated heterostructures, are thoroughly discussed. Finally, various main applications are explored, with primary examples in photodetectors, image sensors, artificial synapses, pressure sensors, structural health monitoring sensors, tactile sensors, and temperature‐strain sensors. While significant progress has established a strong foundation for future research, challenges still exist in 2D‐integrated heterostructures. The challenges and future research directions can be outlined as follows:

### 6.1 Scalability and Manufacturing Bottlenecks

Scalable large‐area growth of 2D materials remains a critical bottleneck for their integration into practical devices. Current fabrication of 2D heterostructures predominantly relies on mechanical transfer methods [306], which have enabled device dimensions up to the micrometre scale [307]. However, the direct synthesis of 2D‐integrated heterostructures comprising multiple materials still faces stringent process control requirements and remains highly challenging [308, 309, 310]. The transition of these architectures from laboratory experiments to industrial platforms requires bridging the gap to wafer‐scale manufacturing. Although recent progress utilising atomic layer deposition (ALD), spin coating, adhesive bonding layers, and commercial wafer bonding equipment has facilitated large area integration [80, 311, 312], achieving true wafer‐scale fabrication and Complementary Metal‐Oxide‐Semiconductor (CMOS) compatibility necessitates large‐area growth. In this context, mixed‐dimensional heterostructures that integrate 2D materials with mature 3D semiconductors offer the most pragmatic pathway by leveraging established Silicon infrastructure [313]. Despite these promising scalable approaches, achieving high repeatability and yield remains a major bottleneck due to the inherent spatial non‐uniformity and random grain boundaries of polycrystalline CVD films. These limitations result in device‐to‐device variations in sensor performance [314, 315]. Furthermore, device packaging introduces a unique paradox: while robust encapsulation is strictly essential to protect fragile vdW interfaces from environmental degradation, these mechanically rigid layers often act as strain‐absorbing barriers that considerably attenuate external mechanical stimuli [316, 317].

### 6.2 Device Reliability, Mechanical Failure, and Cross‐Sensitivity

Although the initial sensing metrics of 2D‐integrated heterostructures are often remarkable, long‐term reliability under cyclic mechanical loading. It still remains a critical unresolved scientific challenge. Continuous strain cycles frequently induce strain fatigue, severe hysteresis, and mechanical failure, such as interlayer sliding, delamination, or tearing at the weakly bonded vdW interfaces [131]. Furthermore, in the pursuit of multifunctional sensors (e.g., simultaneous monitoring of strain, pressure, and temperature using piezoresistive or thermosensitive mechanisms), cross‐sensitivity emerges as a formidable obstacle, severely complicating the accurate decoupling of distinct external stimuli [318]. To effectively improve the translation of these devices, future research must prioritise novel heterostructure designs. The promising directions include interfacial engineering via defect‐anchoring or localised covalent bonding to prevent vdW slip while preserving the intrinsic features of the 2D layers. Additionally, the creation of 3D corrugated or buckled architectures could efficiently manage substantial macroscopic deformations while maintaining the local microscopic strain within the safe elastic limits of the 2D materials [319]. To address cross‐sensitivity, future designs should explore the integration of stimuli‐specific decoupling algorithms or physically isolated reference layers within the mixed‐dimensional heterostructure stack.

### 6.3 Flexible and Wearable Technology

As highlighted earlier, large‐area sensing platforms are essential to validate the practical utility of 2D‐integrated heterostructures. Nevertheless, challenges persist in achieving scalability and flexibility for wearable applications. Although the primary goal for wearables is typically mechanical compliance, dependence on entirely flexible, all‐polymeric substrates often leads to severe thermal drift and hysteresis, which compromise long‐term sensor reliability [264]. Nowadays, the most commercially viable strategy to navigate these collective challenges is the hybrid integration of 2D materials with traditional Micro‐Electromechanical Systems (MEMS). Instead of constructing the entire device from unstable polymers, integrating ultrathin 2D piezoresistive or piezo‐optoelectronic active layers onto standardised, highly crystalline Silicon MEMS structures (such as suspended diaphragms or micro‐cantilevers) offers an optimal solution for the core sensing elements [320]. This hybrid approach successfully synergises the excellent mechanical elasticity and repeatability of bulk Silicon with the exceptional localised strain‐sensitivity of 2D heterostructures, marking a critical step forward in their technological readiness level for advanced, reliable wearable sensing applications [320, 321, 322].

### 6.4 Advanced In Situ Strain Characterization

Currently, the evaluation of strain‐engineered heterostructures predominantly relies on static, ex situ characterisation methods, which are insufficient for capturing the dynamic evolution of materials under stress. To fundamentally understand and mitigate phenomena such as hysteresis and mechanical failure, transitioning toward dynamic in situ strain characterisation is critical [323]. Future research should heavily invest in techniques such as in situ Raman spectroscopy, in situ X‐ray diffraction (XRD), and in situ Transmission Electron Microscopy (TEM) coupled with nano‐electromechanical testing stages [27]. These advanced methodologies will allow researchers to observe real‐time dynamic strain transfer, monitor interfacial sliding, and trace microcrack propagation at the atomic level during device operation [324]. Elucidating these fundamental failure mechanisms is a prerequisite for optimising structural integrity and reliability in ultrasensitive sensors [136].

### 6.5 AI‐Assisted Strain Optimization and Inverse Design

The rapid advancement of artificial intelligence (AI) is accelerating the development of fundamental hardware elements toward greater miniaturisation and integrated functionality [325]. Recent investigations have employed deep learning models, specifically artificial neural networks, to swiftly determine chemical distributions and interface configurations within heterostructures [326, 327]. These outstanding investigations enable the design of next‐generation architectures with superior sensing capabilities [328]. In strain engineering, graph‐based deep learning techniques are employed to forecast deformation patterns and strain magnitudes across a variety of material systems, including fibre‐reinforced composites, multilayer structures, and lattice metamaterials [326]. Consequently, the integration of AI with existing methodologies for precise localisation of strain fields is pivotal for furthering the comprehension and optimisation of 2D‐integrated heterostructures [329]. However, to realistically accelerate the translation of strain‐engineered 2D heterostructures into commercial ultrasensitive sensors, the future paradigm must shift from mere prediction toward AI‐assisted strain optimisation and inverse design. Instead of relying on resource‐intensive, trial‐and‐error experimental approaches, machine learning algorithms should be leveraged to explore vast combinatorial spaces of mixed‐dimensional materials, twist angles, and thickness ratios [327]. This AI‐driven inverse design will enable the rapid identification of optimal heterostructure configurations that maximise piezoresistive or piezo‐optoelectronic sensitivity while simultaneously minimising cross‐sensitivity and strain fatigue. Ultimately, integrating AI not just as an analytical tool, but as a core design engine, is a highly concrete and promising direction for future research [330].

## Conflicts of Interest

The authors declare no conflicts of interest.

## Data Availability

The data that support the findings of this study are available from the corresponding author upon reasonable request.
